# The hen’s egg test for micronucleus induction (HET-MN): validation data set

**DOI:** 10.1093/mutage/geab016

**Published:** 2021-06-03

**Authors:** Kerstin Reisinger, Dagmar Fieblinger, Andreas Heppenheimer, Jürgen Kreutz, Manfred Liebsch, Andreas Luch, Katrin Maul, Albrecht Poth, Pamela Strauch, Eva Dony, Markus Schulz, Thorsten Wolf, Ralph Pirow

**Affiliations:** 4 Henkel AG & Co KGaA, Duesseldorf, Germany; 1 Department of Chemical and Product Safety, German Federal Institute for Risk Assessment (BfR), Berlin, Germany; 2 ICCR-Roßdorf GmbH (formerly Harlan CCR GmbH), Rossdorf, Germany; 3 University of Osnabrueck, Osnabrueck, Germany

## Abstract

The classical *in vitro* genotoxicity test battery is known to be sensitive for indicating genotoxicity. However, a high rate of ‘misleading positives’ was reported when three assays were combined as required by several legislations. Despite the recent optimisations of the standard *in vitro* tests, two gaps could hardly be addressed with assays based on 2D monolayer cell cultures: the route of exposure and a relevant intrinsic metabolic capacity to transform pro-mutagens into reactive metabolites. Following these considerations, fertilised chicken eggs have been introduced into genotoxicity testing and were combined with a classical read-out parameter, the micronucleus frequency in circulating erythrocytes, to develop the hen’s egg test for micronucleus induction (HET-MN). As a major advantage, the test mirrors the systemic availability of compounds after oral exposure by reflecting certain steps of Absorption, Distribution, Metabolism, Excretion (ADME) without being considered as an animal experiment. The assay is supposed to add to a toolbox of assays to follow up on positive findings from initial testing with classical *in vitro* assays. We here report on a validation exercise, in which >30 chemicals were tested double-blinded in three laboratories. The specificity and sensitivity of the HET-MN were calculated to be 98 and 84%, respectively, corresponding to an overall accuracy of 91%. A detailed protocol, which includes a picture atlas detailing the cell and micronuclei analysis, is published in parallel (Maul *et al*. Validation of the hen’s egg test for micronucleus induction (HET-MN): detailed protocol including scoring atlas, historical control data and statistical analysis).

## Introduction

The *in vitro* micronucleus assay (MNvit) (1) is an essential part of genotoxicity test batteries recommended by regulatory agencies in the field of, e.g., cosmetics (2), industrial chemicals (3) or plant protection products (4). It allows the detection of both chromosomal breakage and interference with chromosomal segregation during interphase by easily scoring micronuclei (MNs) in different cell types, making it a scientifically valid alternative to the *in vitro* chromosomal aberration test (5). A retrospective validation confirmed its good sensitivity (5). However, when the assay is combined with other genotoxicity assays in an *in vitro* test battery as requested by different legislations (3), the overall outcome had a rather low specificity (6). Subsequent improvements in the experimental protocol were recently implemented in a revised Organization for Economic Co-operation and Development (OECD) Testing Guideline (1). However, two aspects can hardly be addressed with assays that are based on two-dimensional cell cultures: an efficient metabolic capacity to identify pro-mutagens, as acknowledged in the OECD Test Guideline (TG) for the MNvit (OECD TG 487) (1), and the route of exposure, an aspect referred to in current OECD TGs on *in vivo* genotoxicity testing (7, 8).

To overcome these limitations, complex three-dimensional test systems were introduced into genotoxicity testing and combined with established read-out parameters (9). These assays are intended to complement the existing *in vitro* genotoxicity toolbox by broadening the spectrum of assays for following up on positive results from initial testing with classical methods.

The hen’s egg test for micronucleus induction (HET-MN) represents one of those examples as it combines the analysis of MN frequencies in circulating erythrocytes with standardised and fertilised chicken eggs, which are routinely used for vaccine production (10, 11). As a unique characteristic, the HET-MN is able to mirror certain steps of Absorption, Distribution, Metabolism, Excretion (ADME): At day 8 of egg development, the test compound is applied through a little hole in the eggshell at the blunt end (where the air cell is located) onto the inner shell membrane. During the following 3 days, the compound passes this membrane and is taken up by the highly vascularised chorioallantoic membrane (CAM) prior to the distribution via the blood vessel system. The metabolism of the compound is ensured by respective enzymes in the yolk sac membrane and the developing liver. Finally, the compound and/or its metabolites are actively excreted into the allantois, a bladder equivalent accessible to sampling. In summary, the HET-MN allows for toxicokinetic and toxicodynamic investigations, thereby closing a major gap in *in vitro* genotoxicity testing.

There is ample evidence that the xenobiotic metabolism is well established in the developing chicken egg, see, e.g., (10). In consequence, liver S9 mix, which needs to be added as an external source of metabolising enzymes to two-dimensional cell cultures, is not required to correctly identify pro-mutagens with the HET-MN (10–14). Recent studies (K. Reisinger, in preparation) provided evidence that the intrinsic metabolic capacity between days 8 and 11 of egg development is located in the developing liver and the yolk sac membrane. During this time, the yolk sac membranes also serve as focal point for erythropoiesis. Thus, test compounds are metabolised in close vicinity to the repository of cells, which are used to analyse the chemical’s genotoxic potential. Therefore, a pre-systemic metabolic elimination of a test compound, which is described for some orally administered drugs by the intestinal and hepatic first-past effect, is not expected.

The period between days 8 and 11 of egg development, when the HET-MN is performed, is a highly proliferative state, during which both the blood volume and the number of erythrocytes per blood volume increase exponentially (15, 16). Erythrocytes bearing MNs accumulate in the blood as the spleen is yet not functional to eliminate damaged cells (17), while the background MN frequency is low in the standardised chicken eggs used (18), which are genetically defined by their local suppliers.

As mentioned above, eggs are only used in an early developmental stage, in which no brain activities could be detected (19–21). This premature state is reflected by legislations around the globe, which do not consider the assay as animal experiment (22–26). Thus, the assay can be used to meet legislations, which demand or support *in vitro* methods for regulatory decision making.

Taken together, the HET-MN provides a complex study type exhibiting a liver-like xenobiotic metabolism. Together with the intrinsic characteristics of chicken eggs as summarised above, the HET-MN combines the advantages of an *in vitro* approach with the ability to mirror the systemic availability of chemicals, which is otherwise associated with *in vivo* experiments, while the assay is in line with animal protection regulations and ethical aspects.

The HET-MN protocol as used in the present study is the result of a thorough method development (11–14) after which the assay was transferred to and further optimised together with a second laboratory (10). Until 2012, up to 21 compounds were tested in two laboratories and were all predicted correctly (see Discussion for details; Table 4). Subsequently, three laboratories entered into a cooperation to further investigate the performance of the HET-MN in a validation exercise (after a transfer phase), the results of which are reported here. The validation study included the investigation of more than 30 chemicals being tested double-blinded as well as the evaluation of two prediction models (PMs). Finally, the validation data were used to calculate the predictivity of the assay.

## Materials and methods

### Selection and allocation of coded chemicals

The test chemicals were selected (independent from the study authors) by experts of the genotoxicity group of Cosmetics Europe. The substances were grouped into three categories based on literature data: true negative (TN) and true positive (TP) chemicals, with concordant *in vitro* and *in vivo* genotoxicity and/or carcinogenicity data (Supplementary Table S1, available at *Mutagenesis* Online), as well as ‘misleading positives’ (MP) with positive *in vitro* findings that, however, were not confirmed in *in vivo* studies. The chemicals were purchased from Merck (Darmstadt, Germany) in purities of ≥95%. The validation involved four phases. In phases I–III, chemicals were coded and shipped by staff members at the German Federal Institute for Risk Assessment (BfR) not involved in testing. In phase IV, BioTeSys (Esslingen, Germany) continued the distribution of the coded chemicals. The chemicals were investigated in all three participating laboratories under blinded conditions, with substance codes differing among facilities. Investigators were provided with a limited set of hazard information. In addition, sealed envelopes with the codes and the entire hazard profile were available to safety officers of the three facilities for emergency cases. The envelopes remained sealed and were sent back to the BfR after the experimental phase to prove that the substance identities were not disclosed before unblinding.

### Chemicals

In order to keep a high level of standardisation, the same batches of each of the following chemicals were shared among laboratories: cyclophosphamide monohydrate (CP; CAS no. 6055-19-2, Merck) and isopropyl myristate (IPM; CAS no. 110-27-0; >98% purity; FisherScientific, Schwerte, Germany). The other solvents [ethanol, dimethylsulfoxide (DMSO); >99.7% purity] as well as auxiliary chemicals (disodium citrate, sodium chloride, sodium hydroxide, xylol) were obtained from local suppliers. Giemsa solution (azur eosine/methylene blue solution) and May-Gruenwald solution (eosine/methylene blue solution) were obtained from Merck.

### Chicken eggs

White Leghorn chicken eggs (*Gallus gallus domesticus*) of a defined health status, i.e., specific-pathogen-free (SPF) eggs, were obtained from Valo Biomedia GmbH (www.valobiomedia.com) within 1 day after egg deposition. Care was taken during transport to avoid major temperature variations. After storage at 4–8°C for a maximum of 4 days, eggs were cultivated in the incubator at 37.5 ± 0.5 °C and a humidity of approximately 70% (40–80%) in horizontal position and automatically rotated to simulate natural incubation conditions.

### HET-MN protocol

The validation followed the HET-MN protocol that has recently been published (27) as well as submitted for publication (18), including study design and criteria used for the evaluation of results. The protocol is therefore only briefly summarised here, whereas the study design and the evaluation criteria are described in more detail to support the understanding of the validation results.

After checking for viability and egg weight, intact and appropriately developed eggs were exposed on day 8 of egg development to the test chemicals. In rare cases, chemicals were applied on day 9 of egg development (see Section Dosing regimen). In general, more than six eggs were allocated to dose or control groups at the beginning of experiments to ensure that a sufficiently high number of viable eggs was available at the end of experiments for MN analysis. In case of unknown or high toxicity up to 18 eggs were allocated to respective dose groups, in case of known and low toxicity 8–10 eggs were used (for details, refer to ref. (18)). Chemicals were freshly prepared and applied *via* a small hole in the eggshell at the blunt end (where the air cell is located) onto the inner shell membrane. Blood samples were always taken on day 11 of egg development. Immediately prior to blood sampling, the viability of eggs was checked by candling them under a cold light lamp and only viable eggs were subjected to sampling. Furthermore, the viability within treatment and control groups was determined, i.e., the number of viable eggs of a treatment/control group at the end of an experiment was compared with the number of viable eggs at the beginning of experiments and given as percentage. For sampling, eggs were opened widely around the small hole used for application. Subsequently, the only appearing big blood vessel was identified, and a loop was pulled out and positioned across a plastic strip, which laid on the rim of the opened eggshell. A sample of 3 to 5 µl blood was taken and spread onto a glass slide. Three slides were prepared per egg (one for analysis, two as back-up) and air-dried. Afterwards, slides were stained with a modified Pappenheim staining. Before analysis under a bright field microscope using a 100× magnification, slides were randomised and coded to prevent operator bias during evaluation.

For analysis, 1000 polychromatic erythrocytes (PCE) and normochromatic erythrocytes (NCE) per egg in total were investigated for the presence of MNs. Other cellular effects such as binucleated cells were only recorded.

### Study design

The HET-MN followed the standard design of *in vitro* genotoxicity studies comprising a solubility study, a recommended pre-test, a dose range-finding experiment and, for validation purposes, at least two valid main experiments, while for regulatory testing laboratories may finalise testing after one valid and positive experiment (1).

#### Solubility study

Based on results of development and optimisation phases of the HET-MN protocol, four solvents have been recommended. With first priority deionised water (aqua DI, 300 µL standard volume to be applied on egg membranes, maximum 1500 µL) and IPM (50 µL) were used. In case of low solubility, ethanol (10%, 100 µL) as well as 1% and 10% DMSO (300 µL and 100 µL, respectively) were used to identify the solvent in which the maximum concentration of the test chemical could be applied. The maximum dose was limited to 100 mg per egg (acceptable weight range: 65 ± 4 g), which corresponds to the top dose in the mammalian *in vivo* MN test, i.e., 2000 mg/kg body weight/day (7).

#### Pre-test

This short-time test was used to narrow down the dose range for the subsequent dose range-finding experiment, especially for well soluble compounds. For this purpose, a limited number of eggs, e.g. two per dose group, was exposed to a limited number of doses, e.g. the highest soluble dose and several dilutions, for 0.5 h up to 48 h. The viability of dose groups was recorded and used to design the subsequent experiment.

#### Dose range-finding experiment

The dose range-finding experiment was designed to define the maximum dose for main experiments, which could be limited by the solubility, if it is less than 100 mg/egg, or by the chemical’s general toxicity (for details on toxicity please refer to Section Evaluation of data). In case the dose range-finding experiment met all validity criteria (see Section Evaluation of data), it was accepted as main experiment. Eggs were exposed in line with the schedule of main experiments. Egg viability was the read-out of first priority; most of the laboratories also prepared slides to investigate the MN frequency.

#### Main experiment

Main experiments comprised a solvent control (SC), a positive control (PC), and at least three doses of the test chemical. As the SC groups showed the same low background in DNA damage compared with untreated eggs, a negative control group was omitted. Cyclophosphamide (CP; 0.05 mg CP/egg in aqua DI) was used as PC, in a concentration to induce a moderate increase in MN rate without causing remarkable general toxicity. In phase I, 7,12-dimethyl-benz[*a*]anthracene was used instead of CP as PC in few experiments, which all fulfilled the respective validity criteria. Each control or dose group comprised six viable eggs at the end of experiments to be subjected to the analysis of MN frequency. For validation purposes, at least two main experiments were performed to obtain information on the intra-laboratory reproducibility. For routine testing, a study can already be terminated after the first experiment in case a clear positive call is obtained, i.e, all criteria for a positive call would have been fulfilled as delineated in Section Evaluation of data. Generally, when a second main experiment is performed, the dose spacing is modified, usually by using a tighter spacing, depending on the outcome of the first main experiment.

### Evaluation of data

#### Processing of data

Data on two endpoints were obtained with HET-MN experiments: egg viability and MN rate. (a) The viability in a dose or control group was determined as the percentage of viable eggs at the end of experiments on day 11 in comparison to the number of eggs allocated to the group on day 8. (b) To calculate MN frequency, 1000 cells (PCE and NCE only) per egg in each of six eggs per control/dose group were inspected for the presence of MN. The occurrence of MN in other cells, e.g. primitive erythrocytes (18), was only recorded but not included in the calculation. MN counts were subjected to a Freeman–Tukey (FT) square-root transformation (18, 28) before the group means were calculated.

#### Validity criteria

Before statistical evaluation, experimental data were examined regarding their validity. (i) The experiment needed to follow the pre-defined design, i.e., SC, PC, and at least three dose groups, with six eggs per group and 1000 cells scored per egg. (ii) The viability of control groups and three dose groups at day 11 had to be ≥40%. (iii) For the FT-transformed MN rate, the mean of the concurrent SC (m_SCexp_) had to be equal to or lower than the mean of the historical SC (m_hSC_) plus two times of standard deviation (sd_hSC_) (m_SCexp_ ≤ m_hSC_ + 2∙sd_hSC_). (iv) The mean MN frequency of the concurrent PC (m_PCexp_) had to be equal to or higher than the mean of the historical PC (m_hPC_) minus two times the standard deviation (sd_hPC_) (m_PCexp_ ≥ m_hPC_ – 2∙sd_hPC_). (v) The bioavailability of the test substance was either demonstrated by a dose-dependent decrease in viability of dose groups or an increase in MN frequency. The appearance of alert parameters (e.g. binucleated cells) could also serve as indication of chemical exposure but was not sufficient to fulfil the validity criteria. In case none of these parameters would prove the bioavailability of a test compound, its distribution within the egg has to be shown with analytical measurements of samples taken from blood, allantois or other compartments of the egg (proof of exposure). During the validation with more than 30 coded test compounds, these additional analyses were outside the scope of the exercise.

#### Statistical evaluation

Data of valid experiments were analysed by two PMs. The first one (PM1) checked for the exceedance of a pre-defined threshold, i.e., the mean of the historical SC (m_hSC_) plus four times the standard deviation (sd_hSC_). The Jonckheere–Terpstra (JT) test was used in addition to check for a dose-dependent monotonic increase below the strict threshold using a significance level (*P*) of 0.025. The outcome of PM1 was positive if the threshold was exceeded and/or if the JT test indicated a statistically significant increase. PM2 used the one-sided Umbrella–Williams (UW) test (29), which detects additional shapes of dose-response curves as it compares single as well as pooled-dose groups against the SC (*P* < 0.05) (18).

#### Consideration of biological relevance

In addition to statistical significance, the biological relevance of effects was analysed in line with OECD TG 487 (1). By expert judgement (EJ), it was checked (i) whether the observed MN frequency exceeded the historical control (HC) range (mean of historical SC plus two times the standard deviation) in case data were below the PM1 threshold. Furthermore, (ii) the reproducibility of positive findings was evaluated.

If one experiment showed a statistically significant, dose-dependent increase in MN frequency (thus demonstrating a reproducible effect across the treatment groups), which exceeded the PM1 threshold, this experiment would be sufficient to call the entire study as positive (even if the second experiment was negative). The positive call for the entire study would also apply in case of a statistically significant increase in one dose only (with exceedance of the PM1 threshold) if reproduced in a second experiment. In case none of the criteria applied, and the bioavailability of the test compound was proven, the study was considered negative. If only one (but not both) of the criteria (i) and (ii) were fulfilled, the study was considered equivocal, i.e., further investigation would have been needed to conclude in a positive or negative call.

Note that after the validation exercise, the performance of both PMs was analysed and the threshold of PM1 and the UW test of PM2 were combined to the final PM (18). None of the calls presented in this publication would change when applying this final PM. For transparency reason, in the graphs presented in the Results and Discussion section, the outcomes of PM1 and PM2 are delineated.

## Results and discussion

Three laboratories (Labs A, B, and C) participated in the validation of the HET-MN. The validation exercise was preceded by a transfer phase, in which the HET-MN protocol was implemented in Labs A and B by investigating CP and 7,12-dimethyl-benz(*a*)anthracene; Lab C was not involved in this phase as it already participated in the preceding optimisation phase of the method (10). Subsequently, three chemicals, already tested before with the HET-MN, were shared blinded to all three laboratories to expand the HC databases in Labs A and B (data not shown). In addition, the transfer phase was used to verify the implementation of standards linked to validation exercises (30, 31) such as the shipping of coded chemicals as well as proper dose-range findings and to conclude the studies with coded chemicals.

### Coded testing

The subsequent validation exercise was structured into four phases following a lean design (31). In phase I, each chemical was investigated by all three laboratories to obtain information on within- and between-laboratory reproducibility. In phases II and III, each chemical was tested in two laboratories, whereas in phase IV, each chemical was analysed in one laboratory only to expand the number of chemicals investigated with the HET-MN. In total, 34 chemicals were tested double-blinded in a total of 123 main experiments, the results of which are shown in Supplementary Figures S1–S31, available at *Mutagenesis* Online. Table 1 summarises the final calls of studies, which comprise individual experiments performed with the same chemical. Due to the large number of experiments, only those studies are portrayed in more detail, whose results deviated from *in vivo* genotoxicity or carcinogenicity data (see Supplementary Table S1, available at *Mutagenesis* Online). The description of results starts with 2-aminoanthracene to delineate both the study design and the evaluation criteria.

**Table 1.** Overview of the validation outcome

Figure no.	Chemical	CAS no.	Category	Phase	Results		
					Lab A	Lab B	Lab C
S1	2-Aminoanthracene	613-13-8	TP	IV		pos	
S2	2-Acetylaminofluorene	53-96-3	TP	III	pos		pos
S3	(2-Chloroethyl)trimethyl-ammonium chloride	999-81-5	TN	IV		neg	
S3	2-Ethyl-1,3-hexandiol	94-96-2	MP	IV			neg
S4	2,4-Diaminotoluene	95-80-7	TP	II	pos	pos	
S5	2,4-Dichlorophenol	120-83-2	MP	I	neg	neg	neg
S6	4-Nitroquinoline N-oxide	56-57-5	TP	IV		pos	
S6	4-Vinyl-1-cyclohexene diepoxide	106-87-6	TP	IV			pos
S7	5-Fluorouracil	51-21-8	TP	IV		pos	
S8	8-Hydroxyquinoline	148-24-3	TP (i.p.)	III		pos	pos
S9	Aniline	62-53-3	TP	IV			pos
S10	Benzo[*a*]pyrene	50-32-8	TP	II	neg		neg
S11	Cadmium sulphate	10124-36-4	TP	I	pos	neg	neg
S12	Curcumin	458-37-7	MP	III		nv	nv
S13	Cyclohexanone	108-94-1	TN	II		neg	neg
S14	Diclofenac	15307-79-6	TN	IV		neg	
S15	Dihydroxybenzene	108-46-3	MP	I	neg	neg	neg
S16	Ethionamide	536-33-4	MP	IV		neg	
S17	Ethyl methanesulfonate	62-50-0	TP	IV			pos
S18	Etoposide	33419-42-0	TP	III	pos	pos	
S19	Eugenol	97-53-0	MP	II	neg		equiv
S20	Griseofulvin	126-07-8	TP	I	nv	pos	equiv
S21	Mannitol	69-65-8	TN	I	neg	neg	neg
S22	Mannitol-2^a^	69-65-8	TN	II	neg	neg	
S23	*n*-Butylchloride	109-69-3	TN	IV			neg
S24	Phenanthrene	85-01-8	TN	I	nv	nv	nv
S25	Phthalic anhydride	85-44-9	MP	IV			neg
S26	*p*-Nitrophenol	100-02-7	MP	III	neg		neg
S27	Potassium bromate	7758-01-2	TP	IV		pos	
S28	Potassium dichromate	7778-50-9	TP	II/III	neg	pos	neg
S29	Propyl gallate	121-79-9	MP	III	neg		neg
S30	Resorcinol^b^	108-46-3	MP	IV			neg
S30	Taxol	33069-62-4	TP	IV			pos
S31	*tert*-Butylhydroquinone	1948-33-0	MP	IV		neg	

Chemicals were each tested in a blind-coded manner in two to three laboratories in phases I–III and in one laboratory only in phase IV. Study outcome: equiv, equivocal; neg, negative study (i.e. no increase in MN frequency); nv, not valid; pos, positive study; i.p., intraperitoneal. Classification of chemicals into MP, TN and TP is based on historical *in vitro* and *in vivo* genotoxicity or carcinogenicity data as provided in Supplementary Table S1, available at *Mutagenesis* Online.

^a^Excluded from predictivity calculation as it was mistakenly re-tested.

^b^Excluded from predictivity calculation as it was mistakenly tested in the belief of being different from dihydroxybenzence.

**Fig. 1.**
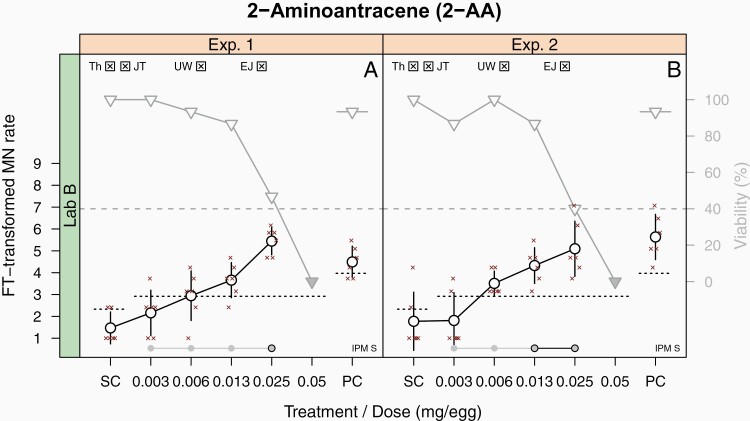
Representative HET-MN study results—2-aminoanthracene. The lab-specific data of two experiments are shown. The FT-transformed MN rate (circles; left axis) and the egg viability (triangles; right axis) are given in relation to the different treatments. Filled triangles indicate viabilities < 40%. MN data are given as mean ± standard deviation and as raw data (small cross symbols). Dotted horizontal lines refer to the MN rate and indicate the upper acceptance limit for the SC and the lower acceptance limit for the PC. MN data were tested for an increase above the threshold (Th) and for a linear trend using the JT test (PM1). MN data were also analysed using the UW procedure (PM2). Finally, the result of the expert judgement (EJ) is indicated. For each test, a positive outcome is indicated by a crossed check box at the top of graphs. Filled circles above the *x*-axis (individual or linked) indicate single- or pooled-dose groups for which the UW test indicated a statistically significant increase; circles with a black outline circles indicate single or pooled dose groups with the smallest significant *p*-value. The used solvent (IPM) is indicated in the low right, and the adjacent label ‘S’ indicates the single-dose regimen.

2-Aminoanthracene (TP; Figure 1, Supplementary Figure S1, available at *Mutagenesis* Online) was investigated up to doses producing signs of strong toxicity as documented by the decline in viability below the cut-off of 40%. Validity criteria (Section Evaluation of data) were all met: (i) the pre-defined experimental design was used, (ii) control groups and a minimum of three dose groups showed a sufficiently high viability of ≥40%, (iii) acceptance criteria for SC and PC (see dotted lines in Figure 1) were met, and (iv) the bioavailability of the chemical was demonstrated by the decrease in viability and the increase in MN frequency (one of these signs would have been sufficient). The evaluation of data with PM1 showed an increase in MN frequency exceeding the pre-defined threshold (see dotted lines in Figure 1). This threshold was calculated as the mean of the historical SC plus four times the standard deviation. Note that the criterium, which is often used as upper bound of the HC range, i.e., the mean of the historical SC plus two times the standard deviation, is used here as validity criterium for the concurrent SC. Therefore, the exceedance of the PM1 threshold is considered a clear indication for a genotoxic effect. In addition, the trend test for a monotonic increase, i.e., JT test, was positive as well. A statistically significant increase was also signalised by the UW test of PM2. In addition to the statistical evaluation, the laboratory evaluated the biological relevance of the observed effects in an EJ (Section Evaluation of data, in accordance to OECD TG 487 (1)); all relevant criteria were met, so that the statistically based test outcome could be confirmed. A second main experiment was performed to obtain information on the within-laboratory reproducibility (WLR) during the validation exercise, which resulted in the same positive call.

Application of **2-acetylaminofluorene** (2-AAF; TP; Supplementary Figure S2, available at *Mutagenesis* Online) induced a dose-dependent increase in MN frequency in the first experiment of Lab A; the increase plateaued slightly below the PM1 threshold and declined at the highest dose. This umbrella-shaped dose-response curve was picked up by the UW test of PM2 but not by the JT test of PM1. A different dose spacing in the second main experiment led to both an above-threshold increase in MN frequency of two dose groups and a dose-dependent decrease in viability below the 40% cut-off. The lab therefore classified the substance as positive in line with published *in vivo* data (Supplementary Table S1, available at *Mutagenesis* Online). Lab C considered IPM instead of DMSO (which was used by Lab A) as the most suitable solvent but applied lower doses. To maximise the applied dose, eggs were not only treated on day 8 but received the same dose also on days 9 and 10, followed by the usual sampling on day 11. This so-called ‘repeated-dose regimen’ (13) induced a dose-dependent increase in MN frequency above the PM1 threshold in experiment 1. This outcome was reproduced in experiment 3 being performed to follow up the disconcordant result in the second experiment. In summary, 2-AAF was correctly classified as positive by Lab C. Details on the ‘repeated-dose-regime’ are given in Section Dosing regimen.

**(2-Chloroethyl)trimethyl-ammonium chloride** (TN; Supplementary Figure S3, available at *Mutagenesis* Online) was tested by Lab B up to doses producing signs of strong toxicity with a decrease in viability below the cut-off of 40%, thereby proving the bioavailability of the chemical. As the MN frequency in the dose groups was similar or even below SC values, both experiments and in consequence the study was considered negative in line with published *in vivo* data (Supplementary Table S1, available at *Mutagenesis* Online).

**2-Ethyl-1,3-hexandiol** (MP; Supplementary Figure S3, available at *Mutagenesis* Online) was tested by Lab C up to doses producing signs of strong toxicity. In the second experiment, the MN rate at the second mid-dose was flagged by both PMs. As this effect was not reproducible, neither in the third main experiment using a tight dose range nor in the dose-range-finding experiment, the study was considered negative in line with historical *in vivo* data (Supplementary Table S1, available at *Mutagenesis* Online).

Already in the dose-range finding experiment (not shown), Lab A observed strong toxicity when using **2,4-diaminotoluene** (TP; Supplementary Figure S4, available at *Mutagenesis* Online) in low concentrations, i.e., 0.5–1.5 mg/egg, as well as at 20 mg/egg. The chemical was therefore only tested up to 15 mg/egg in the main experiments, reproducing the strong toxicity in the low dose groups. The treatment caused a dose-dependent increase in MN frequency above the PM1 threshold. Lab B applied higher doses only and observed strong toxicities at 60 mg/egg and above. The testing of higher doses revealed a plateau-shaped dose–response curve with a clearly increased MN rate above the PM1 threshold. Both labs correctly classified the substance as positive.

The 2,4-diaminotoluene study of Lab B is one example in which egg viability was lower compared with data obtained in the two other laboratories, a phenomenon seen in phases I and II (Supplementary Figure S34, available at *Mutagenesis* Online), i.e., in the studies with mannitol, griseofulvin and phenanthrene. The cause could not be identified, but this variation disappeared in the further course of the validation, i.e. the viability values in Lab B approached those of the other laboratories. PC groups were also affected by a temporarily reduced viability of ~60%, which returned to 100% in later phases. Importantly, these viability issues did not invalidate the experiments and the outcome of respective studies were concordant with published *in vivo* data.

All three laboratories tested 2,4-dichlorophenol (MP; Supplementary Figure S5, available at *Mutagenesis* Online) in phase I up to doses producing signs of strong toxicity: Labs A and C in the pre-test and dose-range-finding experiments only; Lab B also in the first main experiment, while the main experiments mainly involved sub-toxic doses. There was no increase in MN frequency, neither in the main experiments (Supplementary Figure S5A–G, available at *Mutagenesis* Online) nor in the dose-range-finding experiments. As the bioavailability was shown, especially when considering pre-tests or dose-range finders, the three studies were considered valid and negative in line with historical *in vivo* data (Supplementary Table S1, available at *Mutagenesis* Online).

**4-Nitroquinoline 1-oxide** (TP; Supplementary Figure S6, available at *Mutagenesis* Online) induced an increase in MN frequency above the PM1 threshold already at a very low dose of 0.03 mg/egg in Lab B. The clear positive call, which was confirmed in the second main experiment, is consistent with published *in vivo* data (Supplementary Table S1, available at *Mutagenesis* Online).

Lab C applied 2 mg/egg of **4-vinyl-1-cyclohexene diepoxide** (TP; Supplementary Figure S6, available at *Mutagenesis* Online) as top dose in the main experiments, after the dose-range-finding experiment had revealed a clear decrease in viability at higher doses. The dose-dependent increase in MN frequency in both main experiments was sufficient to prove the bioavailability of the chemical and to classify the substance as positive in line with the chemical’s classification as ‘true positive’ (Supplementary Table S1, available at *Mutagenesis* Online).

**5-Fluorouracil** (TP; Supplementary Figure S7, available at *Mutagenesis* Online) was tested up to doses producing signs of strong toxicity (viability below the 40% cut-off) by Lab B, which observed in the first main experiment a steep decrease in viability at the highest dose and a clear induction of the MN rate at the second highest dose. After the dose spacing was adapted in the second main experiment, a moderate dose-dependent decrease in viability occurred which was accompanied by an above-threshold increase in the MN rate in all dose groups, confirming the positive call of the first experiment in line with *in vivo* data (Supplementary Table S1, available at *Mutagenesis* Online).

**8-Hydroxyquinoline** (8HQ; TP; Supplementary Figure S8, available at *Mutagenesis* Online) was tested up to strong toxicity in two laboratories which both observed a dose-dependent increase in MN frequency, exceeding the PM1 cut-off at the very same dose (0.4 mg/egg) in each of the four experiments (Supplementary Figure S8A–D, available at *Mutagenesis* Online). Although the MN frequency in the SC of both experiments of Lab B slightly exceeded the acceptance threshold, the experiments were considered valid based on the following considerations. The SC validity cut-off is defined by the mean of the historical SC plus two times the standard deviation, which means that 95% of the HC data fall within the acceptance range if the distribution of data is approximately normal (and the historical database is sufficiently large to reliably derive the SC validity cut-off from the standard normal instead of the *t*-distribution). An exceedance of the acceptance threshold by any concurrent SC could thus occur on average in 1 of 20 repetitions simply by chance in a test system being ‘under control’. As the SC values in the experiments of Lab B were only slightly above the cut-off, and since 8HQ induced a clear increase in MN frequency, both control values and the experiments were considered valid.

The studies in both laboratories were in line with *in vitro* genotoxicity data for 8HQ (32, 33). *In vivo* genotoxicity and carcinogenicity studies with oral administration showed disconcordant results (Supplementary Table S1, available at *Mutagenesis* Online). However, when using a single intraperitoneal (i.p.) injection and analysing PCE/NCEs in the bone marrow of CD1 mice, a clear increase in MN frequency was seen (34). In addition, several rodent lifetime studies have been published in which 8HQ was applied i.p., via the vagina, or as bladder implant (35). In all these studies, the treated animals developed tumours at the site of application or in other organs at a rate exceeding that in the SC group. In line with these application regimens, the HET-MN requires an application of chemicals onto the inner shell membrane, which can easily be permeated, allowing the chemical to penetrate the CAM, which is pervaded by fenestrated blood vessels, facilitating the systemic uptake. In consequence, we consider the administration procedure in the HET-MN studies to be more closely related to an i.v. administration rather than to application via the oral route. Thus, the two positive HET-MN studies for 8HQ were considered consistent with published *in vivo* data.

**Aniline** (TP; Supplementary Figure S9, available at *Mutagenesis* Online) was tested up to strong toxicity in Lab C. The concentration range was narrowed in the second main experiment to further investigate the range around the dose that caused a slight exceedance of the PM1 cut-off in the first experiment. In the second one, the MN frequency at all dose groups was above the PM1 threshold. Thus, the study was in line with a variety of *in vivo* MN tests in mice and rats showing positive responses in the bone marrow or peripheral blood after oral or i.p. administration (36–41), supporting the GHS classification as ‘suspected of causing genetic effects’ (GHS Muta 2, H341) (42).

The exposure of eggs to very low doses of **benzo[*a*]pyrene** (BaP; TP; Supplementary Figure S10, available at *Mutagenesis* Online), 0.03 and 0.04 mg/egg, caused a strong decrease in viability below 40% in Labs A and C. In Lab A, the MN rate in both main experiments was similar to the values of the SC. Such a negative outcome was also seen in Lab C in the first experiment. In the second one, the slight increase in MN rate in lowest dose group was picked up by PM2. As this response was not dose dependent and given the fact that the complete absence of detectable MN in the SC supported reaching the statistical significance in PM2, the effect was not considered biologically relevant. In consequence, the studies were disconcordant to historical *in vivo* data (Supplementary Table S1, available at *Mutagenesis* Online). After the validation exercise, follow-up experiments were performed with a modified application scheme, which enabled an exposure of eggs with higher doses without inducing strong toxicity; these conditions were favourable to reveal the expected increase in MN frequency (for details, see Section Dosing regimen).

Similarly, studies with **cadmium sulphate** (TP; Supplementary Figure S11, available at *Mutagenesis* Online) showed a strong toxicity (viability below the 40% cut-off) at low doses starting with 0.03 mg/egg in Lab A. This was accompanied by a dose-dependent increase in MN rate, which was picked up by both PMs and thus confirmed *in vivo* genotoxicity and carcinogenicity data (Supplementary Table S1, available at *Mutagenesis* Online). The two other laboratories reproduced the dose-dependent effect on egg viability starting at 0.04 mg/egg. In addition, a dose-dependent sub-threshold increase in MN frequency could be observed in both laboratories in the first experiments, which was flagged by the JT trend test of PM1 while one dose group of each experiment was outside the HC but below the PM1 threshold. As these effects were only slight and not reproducible in the second experiments, the studies were considered negative overall.

It should be noted that Lab C tested cadmium chloride in a repeated-dose regimen to maximise the overall dose by three applications. After the validation exercise, the laboratory re-tested the chemical in a single-dose regimen, which revealed a clear increase in MN frequency (for details, see Section Dosing regimen).

**Curcumin** (MP; Supplementary Figure S12, available at *Mutagenesis* Online) showed a limited solubility in all recommended solvents. IPM was eventually selected by Lab C as the most suitable one to produce a homogenous suspension at ≥0.075 mg/egg. According to standards established for determining the maximum concentration for poorly soluble test chemicals (OECD TG 487, MNvit), curcumin was tested up to the first precipitating dose, i.e., 0.1 mg/egg without any impact on MN frequency or egg viability. As the precipitations on the egg membrane did not interfere with the test system’s integrity and therefore not with the experimental outcome, Lab B applied suspensions to the highest manageable dose which could be applied on eggs (20 mg/egg). Again, MN frequency was equal to or below the SC values while viability remained high. Consequently, both studies could not be regarded as valid since the bioavailability of the chemical was not proven. As analytical methods to prove the test chemical’s distribution within the biological test system were not foreseen for the validation exercise, it was decided to present and discuss the study results without including them in the calculation of predictivity.

**Cyclohexanone** (TN; Supplementary Figure S13, available at *Mutagenesis* Online) was tested up to strong toxicity in Labs B and C, resulting in similar top doses. Due to the absence of relevant increases in MN frequency in all main experiments, both laboratories classified the substance as negative in line with *in vivo* data (Supplementary Table S1, available at *Mutagenesis* Online).

**Diclofenac** (TN; Supplementary Figure S14, available at *Mutagenesis* Online) was tested by Lab C with doses spanning from low to strong toxicity. As none of the experiments showed genotoxic effects, the study was considered negative in concordance with historical *in vivo* data (Supplementary Table S1, available at *Mutagenesis* Online).

**Dihydroxybenzene** (MP; Supplementary Figure S15, available at *Mutagenesis* Online) was tested in phase I by the three laboratories and was provided again to Lab C in phase IV as **resorcinol** in the assumption of being a different substance (MP; Supplementary Figure S30, available at *Mutagenesis* Online). In all four studies, the chemical was applied in aqua DI up to doses inducing strong toxicity in the main experiments with the exception of Lab C, which tested up to strong cytotoxicity in the dose-range finding experiment (data not shown). None of the studies showed an increase in MN frequency. In consequence, this outcome did not confirm the positive findings of classical *in vitro* genotoxicity assays but was in line with the negative outcome of *in vivo* studies, which are considered of higher relevance (Supplementary Table S1, available at *Mutagenesis* Online).

**Ethionamide** (MP; Supplementary Figure S16, available at *Mutagenesis* Online) was investigated by Lab B up to strong toxicity in the first main experiment. As this experiment and the second one involving a modified dose range did not show a significant increase in MN frequency, the study was considered negative in line with historical *in vivo* data (Supplementary Table S1, available at *Mutagenesis* Online).

**Ethyl methanesulfonate** (TP; Supplementary Figure S17, available at *Mutagenesis* Online) induced a clear increase in MN rate in both main experiments provided by Lab C, which was in line with published *in vivo* data (Supplementary Table S1, available at *Mutagenesis* Online).

**Etoposide** (TP; Supplementary Figure S18, available at *Mutagenesis* Online) was tested in Lab A in very low concentrations of 0.013–0.2 mg/egg, which caused a dose-dependent decrease in viability in parallel to a dose-dependent increase in MN frequency, clearly exceeding the PM1 threshold already at the lowest dose. In contrast to Lab A, which used aqua DI as solvent, Lab B chose IPM, thereby being able to apply slightly higher doses of 0.63–5 mg/egg, which also induced a clear increase in MN rate. In consequence, both studies were considered positive being concordant with historical *in vivo* studies (Supplementary Table S1, available at *Mutagenesis* Online).

**Eugenol** (MP; Supplementary Figure S19, available at *Mutagenesis* Online) was tested in Lab A up to 1 mg/egg showing a dose-dependent decrease in viability below the 40% threshold in both experiments without indication of genotoxic effects. Lab C observed strong toxicity with similar dose groups. While no genotoxic effects were observed in the first experiment, an increase in MN frequency with the highest dose in the second experiment was detected, which was picked up by both PMs and was considered relevant in the EJ. However, as this single event was not reproducible, the entire study was considered equivocal because some but not all criteria for a positive call were met while bioavailability was proven (i.e., reproducibility was missing).

**Griseofulvin** (TP; Supplementary Figure S20, available at *Mutagenesis* Online) is a hardly soluble anti-fungal drug applied in nail enamels. Several studies are available, e.g. (43–45), which tried to improve the chemical’s solubility for a more effective medication. In the current study, all three labs chose aqua DI, the only solvent in which a homogeneous suspension could be prepared, in comparison to the even poorer solubility in the other ones. Lab B observed a dose-dependent decrease in viability, which was confirmed in the second main experiment. Also, the second experiment revealed a dose-dependent increase in MN frequency, which exceeded the PM1 threshold at the highest dose (50 mg/egg) and which was statistically flagged by both PMs, so that the criteria for two valid studies and a positive call were fulfilled. (Please refer to the paragraph on 2,4-diaminotoluene for the different egg viability profile in Lab B in that validation phase).

In contrast, Lab C did not observe effects on viability in both experiments, while in the second one, a dose-dependent increase in MN frequency was revealed, proving the chemical’s bioavailability. The MN rate in the highest dose, being slightly below the PM1 threshold, was picked up by both the JT test and the UW test. As this dose group remained the only one, which indicated DNA damage, the study was considered equivocal as some but not all criteria for a positive call were fulfilled, i.e, reproducibility of effects could not be shown.

In Lab A, several slight effects were detected. In experiment 1, the viability decreased to 86% which is within the normal range of SC and PC (Supplemental Figure S34, available at *Mutagenesis* Online). In both experiments, the MN rate of one dose group was slightly outside the HC, but clearly below the PM1 threshold. These slight effects were not considered sufficient by the laboratory to prove the chemical’s bioavailability. In consequence, the study was considered not valid, and in line with the process used for curcumin and phenanthrene, the griseofulvin study of Lab A was not included in the predictivity calculation.

**Mannitol** (TN; Supplementary Figure S21, available at *Mutagenesis* Online) was investigated in aqua DI in all three laboratories. Labs A and C tested up to the maximum dose of 100 mg/egg and observed a decrease in viability, which was sufficient to prove the chemical’s bioavailability. In contrast, Lab B observed already at 15 mg/egg (first experiment) a decrease in viability reaching the threshold defining strong toxicity. As none of the laboratories observed genotoxic effects, the studies were considered negative in line with published *in vivo* data (Supplementary Table S1, available at *Mutagenesis* Online).

Mannitol was mistakenly re-tested in phase II (Supplementary Figure S22, available at *Mutagenesis* Online) by two laboratories. Lab A reproduced the findings. By using IPM instead of aqua DI, Lab B chose a different solvent resulting in a lower top dose; nevertheless, both bioavailability and the absence of genotoxic effects were confirmed, resulting in the fifth negative and thus correct call. Note that only the phase-I studies of mannitol were considered for predictivity calculation.

***n*-Butyl chloride** (TN; Supplementary Figure S23, available at *Mutagenesis* Online) was tested in Lab C at doses groups causing responses from low to strong toxicity (viability < 40%). As the viability declined steeply (without any changes in MN rate) in the first experiment at the highest dose, the dose range was modified in the second experiment, which proved the absence of genotoxic effects. The negative call was in line with historical *in vivo* data (Supplementary Table S1, available at *Mutagenesis* Online).

**Phenanthrene** (TN; Supplementary Figure S24, available at *Mutagenesis* Online) was tested in all laboratories up to the maximum solubility of 7 mg/egg, while 11 mg/egg was identified as the maximum applicable suspension. Labs A and C did not observe any relevant impact on viability, even when Lab C used the repeated-dose regimen to facilitate the application of 30 mg/egg in total, i.e., three times 10 mg/egg on days 8, 9 and 10. The reduction of viability in Lab B was considered less relevant because the viability seemed to be generally impacted in these studies as also the viability in the PC of both experiments was close to 60%, i.e., a treatment condition which normally does not affect viability. No indications for genotoxic effects were observed. Similar to curcumin, phenanthrene could not be appropriately investigated as the bioavailability of the test compound could not be demonstrated, neither by an increase in MN frequency nor by a decrease in viability. As analytical methods to prove its distribution in the test system were not planned to be used in this validation exercise, is was decided to show and discuss the studies but to not include the results in the calculation of the predictivity.

**Phthalic anhydride** (MP; Supplementary Figure S25, available at *Mutagenesis* Online) was tested up to strong toxicity in Lab C without evidence for genotoxicity in the first main experiment. In the second experiment, the MN frequency increased close to the PM1 threshold at 7 mg/egg, an effect accompanied by strong toxicity (MN data not shown in the graph due to the viability of <40%). The following experiment conducted with a narrowed dose range confirmed the absence of genotoxic effects also in the two highest doses which were accompanied by strong toxicity. The study was concluded negative, concordant to published *in vivo* data (Supplementary Table S1, available at *Mutagenesis* Online).

**p-Nitrophenol** (MP; Supplementary Figure S26, available at *Mutagenesis* Online) was investigated by Labs A and C up to strong toxicity, proving the bioavailability of the test chemical. Whereas Lab C did not observe indications for genotoxicity, Lab A detected a slight increase in MN frequency in a mid-dose (without dose dependency) in the first main experiment, which was flagged by PM2. As this effect was not reproduced in any of the dose groups tested in the second main experiment, also this study was considered negative in line with historical *in vivo* data (Supplementary Table S1, available at *Mutagenesis* Online).

**Potassium bromate** (TP; Supplementary Figure S27, available at *Mutagenesis* Online) produced a dose-dependent increase in MN frequency above the PM1 threshold in the first main experiment in Lab B. The positive call was confirmed in the second main experiment, so that the study was considered positive in line with published *in vivo* experiments (Supplementary Table S1, available at *Mutagenesis* Online).

**Potassium dichromate** (TP; Supplementary Figure S28, available at *Mutagenesis* Online) was tested by Lab B up to strong toxicity. The three valid dose groups (0.01, 0.05 and 0.1 mg/egg) in the first main experiment induced a sub-threshold increase in MN frequency, which was flagged by the JF test and the UW test. The dose range between 0.1 and 0.5 mg/egg, which showed a steep toxicity curve, was further investigated in the second main experiment. This revealed a dose-dependent increase in MN above the threshold being flagged by both PMs. The study was therefore considered positive. Lab C tested potassium dichromate in the same solvent but did observe a strong toxicity at 0.18 mg/egg without any signs of DNA damage. In the second main experiment, a sub-threshold increase in MN frequency in the lowest dose group was flagged by PM2. As the MN frequency of the other dose groups in both main experiments were similar to those of the SC, the study was considered negative. Because of the divergent results between Labs B and C, the chemical was shared additionally to Lab A in the following phase III. Lab A reproduced the findings of Lab C by demonstrating strong toxicity at and above 0.2 mg/egg without indications of DNA damage. After the validation, further testing was performed with a modified dosing regimen delineated in Section Dosing regimen.

The top dose of **propyl gallate** (MP; Supplementary Figure S29, available at *Mutagenesis* Online) was determined in Lab A by strong toxicity without indications for genotoxicity, leading to a negative call. Lab C also observed a clear decrease in viability, proving the bioavailability of the compound, while the absence of genotoxicity led to the second negative call of the chemical in line with published *in vivo* genotoxicity data (Supplementary Table S1, available at *Mutagenesis* Online).

The maximum dose of **taxol** (TP; Supplementary Figure S30, available at *Mutagenesis* Online) applied by Lab C, i.e., 0.016 mg/egg, was determined by the chemical’s limited solubility. Despite the low doses applied, increases in MN frequency clearly above the PM1 threshold were detected in both main experiments, supporting the positive call in line with published data of the aneugen (Supplementary Table S1, available at *Mutagenesis* Online).

**Tertiary-butylhydroquinone** (TP; Supplementary Figure S31, available at *Mutagenesis* Online) was investigated up to strong toxicity. In the first experiment, the highest dose induced an increase in MN frequency above the PM1 threshold, which was flagged by both PMs. However, as none of the dose groups in the following two experiments were flagged by the PMs, the study was considered negative.

### Assessment of intra- and inter-laboratory reproducibility

In order to assess the intra- and inter-laboratory reproducibility, all data generated within the validation effort under blinded conditions (Supplementary Figures S1–S31, available at *Mutagenesis* Online) were tabulated (Table 1, Supplementary Tables S2, available at *Mutagenesis* Online).

The reproducibility of the HET-MN assay within a laboratory over time was assessed by comparing the concordance of experiments performed in duplicate or triplicate in the same laboratory. Among the 48 studies performed across all three laboratories, 101 experiments could be identified and counted towards assessing the concordance of classification (Table 2A, Supplementary Table S2, available at *Mutagenesis* Online). The overall within-laboratory reproducibility for the validation exercise was 92% (Table 2A), with values between 88 and 94% for the individual laboratories that participated in the validation.

**Table 2.** (A) Reproducibility within one laboratory over time (within-laboratory concordance), (B) reproducibility between laboratories (between-laboratory concordance) and (C) predictivity

(A) Category	Discordant	Concordant	Total	%
Lab A	1	11	12	88
Lab B	1	16	17	94
Lab C	2.3	16.7	19	88
All labs	4	44	48	92
(B) Discordant	Concordant	Total	%	
1.8	12.2	14	87	
(C) Category	Lab A	Lab B	Lab C	Overall
Sensitivity (%)	67	89	67	84
Specificity (%)	100	100	95	98
Accuracy (%)	83	94	82	91

Reproducibility between laboratories was calculated based on the final overall call within laboratories for each chemical obtained when tested in three or two laboratories during phases I–III. Of these 15 chemicals, 87% obtained concordant calls (see Table 2B, Supplementary Table S2, available at *Mutagenesis* Online).

Both the intra- and inter-laboratory reproducibility were found to be in a similar range to other *in vitro* genotoxicity assays when testing was done in a coded fashion and was therefore considered acceptable, i.e., the intra-laboratory reproducibility of the *in vitro* MN was reported to vary between 83 and 100% (5).

### Predictive capacity of the HET-MN

The predictive capacity of the HET-MN was calculated using the data from 29 chemicals from all phases of the validation exercise (Table 1). Where the call for a chemical unequivocally agreed with the expected classification, it was assigned a value of 1.0 when applied to the calculation. If it unequivocally disagreed with the expected classification, that chemical was assigned a value of 0, while equivocal calls counted as 0.5. Discordant calls for one chemical among laboratories went in according to their weight, e.g., if a chemical was tested in three labs and two found the expected results and one gave an unexpected result it would be assigned a value of 0.66. Applying these principles revealed an overall sensitivity of the HET-MN of 84% (Table 2C). The overall specificity was 98%. Only eugenol produced one equivocal experiment and, in consequence, an equivocal study while the remaining studies with TN and MP concluded in correct negative predictions (Table 2C). The 16 chemicals with reported positive *in vivo* genotoxicity findings covered those undergoing metabolisation in connection with DNA damage (2-aminoanthracene, 2-AAF, 2,4-diaminotoluene, aniline, BaP) (46–50), those with an underlying aneugenic mechanism (taxol, griseofulvin, 8-hydroxyquinoline) (47, 48, 51), metal salts acting *via* different mechanisms including oxidative stress (potassium dichromate, cadmium sulphate) (52–54), alkylating agents (ethyl methanesulfonate, 4-vinyl-1-cyclohexene diepoxide, 4-nitroquinoline 1-oxide) (47, 55, 56), a nucleoside analogue (5-fluorouracil) (46), potassium bromate that induces oxidative stress (57) and a topoisomerase inhibitor (etoposide) (46). The sensitivity was calculated to be 84%. While Lab B observed a sensitivity of 89%, it was 67% in Labs A and C. The incorrect calls in the latter two laboratories originate from four chemicals. Three of them were further investigated after the validation revealing a dose-dependent increase in MN frequency, i.e., BaP and potassium dichromate with the day-9 protocol after strong toxicity was observed at dose groups < 0.03 and 0.18 mg/egg, respectively. In addition, CdSO_4_ was re-tested in the standard protocol after it has been evaluated with the ‘repeated-dose’ regimen during coded-testing, a dosing regimen that was deprioritised after the validation (for details, refer also to Section Dosing regimen). The fourth chemical was griseofulvin, which limited solubility has been highlighted above. Lab B did not test all of the four chemicals. The overall accuracy of the HET-MN was calculated to be 91%.

In order to put the predictivity of the HET-MN into reference, the validation outcome was compared with the predictivity of the MNvit for which two data sets were available. First, a retrospective analysis of MNvit data published in 2008 (5) in order to support establishing the OECD TG 487. This data set is however not discussed in further detail here because the predictivity of the MNvit data set was calculated with reference to data of the *in vitro* chromosomal aberration test to which the MNvit was supposed to function as an alternative. Generally, validation data are rather set in reference to *in vivo* data, which are considered of higher biological relevance compared with *in vitro* results. Therefore, another study was used to evaluate the HET-MN data set. In specific, a respective analysis of MNvit data referenced to *in vivo* data (6) revealed a sensitivity of the classical MNvit of 78.7%, while specificity was 30.8% (or 53.8% when the chemicals classified as equivocal *in vivo* were considered negative). It should be noted that the MNvit results used for the calculation were obtained with different cell lines and not with one test system as used for the current validation.

### Protocol improvements

Apart from providing key information of the predictive capacity of the HET-MN, the comprehensive validation data set was additionally used to investigate specific protocol aspects, which are addressed in the following.

#### Dosing regimen

In the development and optimisation phases of the assay, three different dosing regimens were used (10): the standard protocol involving the single application on day 8; the ‘repeated-dose’ regimen with repeated dosing on days 8, 9 and 10; and a single-dose regimen with application on day 9. All regimens foresee a sampling on day 11. The usefulness of the two non-standard regimens is discussed in the following.

##### Repeated-dose regimen

During the validation phase, Labs A and B employed exclusively the standard protocol. Lab C, which already participated in the optimisation phase, additionally used the ‘repeated-dose’ regimen, which foresees a repeated administration of the same dose on three consecutive days. This treatment procedure was developed to maximise the applicable dose in comparison to a single exposure in case of a low solubility of test chemicals while in parallel an increase in viability could often be observed (10). In the validation study, the ‘repeated-dose’ regimen was applied for cadmium sulphate and phenanthrene in phase I and for 2-AAF in phase III.

**Fig. 2.**
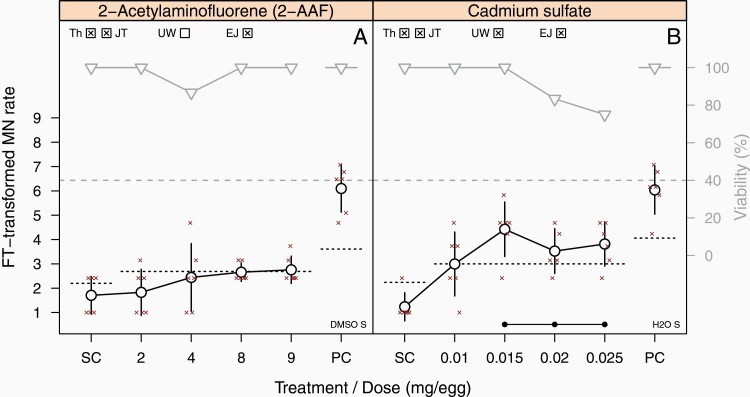
Chemicals re-tested non-coded with the standard protocol after using the ‘repeated-dose regimen’ during validation. One experiment each with 2-AAF and cadmium sulphate is shown. For further graphic details, see legend of Figure 1.

In case of cadmium sulphate, Lab C was able to double the dose when using the ‘repeated-dose’ regimen in comparison to the other laboratories (Supplementary Figure S11E and F, available at *Mutagenesis* Online). However, signs of strong toxicity occurred at doses similar to those of the other laboratories without observing an impact on MN frequency. An increase in MN frequency could, however, be observed when the lab re-tested cadmium sulphate with the standard protocol after the validation exercise (Figure 2). Phenanthrene could be applied in threefold higher doses with the ‘repeated-dose’ regime (Supplementary Figure S24E and F, available at *Mutagenesis* Online), but the laboratory nevertheless faced the same problem as with the single-dose regimen used in the in the other laboratories: The viability remained high, and the bioavailability could not be proven. Only the repeated-dose study with 2-AAF produced a correct call (Supplementary Figure S2C–E, available at *Mutagenesis* Online) in line with published *in vivo* data. After coded testing, the laboratory re-tested the chemical and correctly predicted the chemical using the standard design (Figure 2A). Thus, as the ‘repeated-dose regimen’ was shown to be of limited value in supporting correct calls, it is no longer be described in the HET-MN protocol (18).

##### Day-9 protocol

**Fig. 3.**
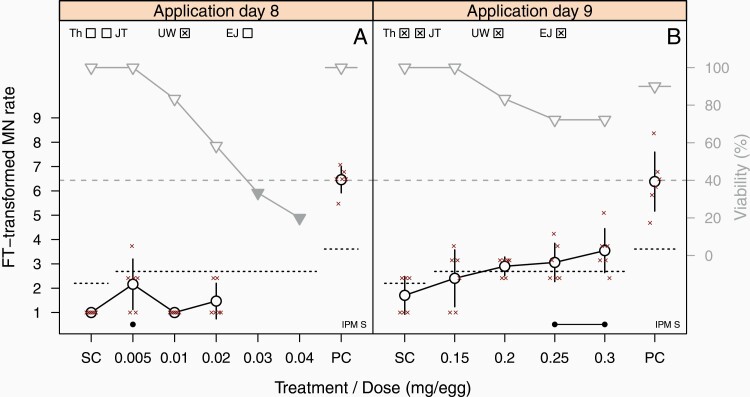
Comparison of the standard protocol with the ‘day 9 protocol’ using benzo[*a*]pyrene. Eggs were either treated on day 8 of egg development (left) or on day 9 (right) with the indicated doses of benzo[*a*]pyrene, while sampling was done on day 11 in the laboratory, which performed both experiments. For further graphic details, see legend of Figure 1.

The third dosing regimen was conceived during the development and optimisation phases of the assay (10) and came into play in response to the effects observed with BaP, i.e., strong toxicity in the absence of genotoxic effects already at very low doses (0.03 mg/egg; Supplementary Figure S10, available at *Mutagenesis* Online) when compared with the top dose considered for the HET-MN of 100 mg/egg (aligned with OECD TG on the *in vivo* MNT (7). BaP was therefore re-tested after the validation phase with a slightly modified protocol in which single doses were applied on day 9 (instead of day 8 according to the standard protocol), whereas sampling remained on day 11. With this modification, 10-fold higher BaP doses could be applied without inducing strong toxicity, while a clear increase in MN frequency was noticed (Figure 3, Table 3).

**Table 3.** Chemicals tested after the validation with the shortened study protocol starting at day 9 of egg development compared with coded testing, which started at day 8

Chemical	CAS no.	Category	Results	Results
			Day 8–11	Day 9–11
			Correct studies/total studies	
Benzo[*a*]pyrene	50-32-8	TP	0/2	Pos
Eugenol	97-53-0	MP	1.5/2	Neg
p-Nitrophenol	100-02-7	MP	1/1	Neg
Potassium dichromate	7778-50-9	TP	1/3	Pos
Resorcinol	108-46-3	MP	4/4	Neg

Neg, correct negative study; Pos, correct positive study.

To investigate whether this approach is of broader relevance, further chemicals were tested with the day-9 protocol after the validation. Similar to BaP, potassium dichromate had also produced strong toxicity at low doses (0.18 mg/egg) when applied on day 8 (Supplementary Figure S28 and S33A, available at *Mutagenesis* Online); the single application on day 9 enabled the application of twofold higher doses without producing strong toxicity, while a dose-dependent increase in MN frequency could be observed (Supplementary Figure S33B, available at *Mutagenesis* Online). It is worth noting that more than half (10/16) of the TP chemicals, when tested with the standard protocol, induced a clear increase in the MN rate at low doses ≤ 1 mg/egg. This applies to 2-AA, 2-AFF, 4-nitroquinole N-oxide, 4-vinyl-1-cyclohexene diepoxide, 5-flurouracil, 8HQ, ethyl methanesulfonate, etoposide, potassium bromate and taxol. Two of the TPs were found to be positive at doses of >10 mg/egg (aniline; 2,4-diaminotoluene) and the hardly soluble griseofulvin was detected at >50 mg/egg. A general reduction of the top dose for HET-MN experiments was not considered in order to avoid failing to detect other TPs in future experiments. In this context, it is important to note that the application of MPs and TNs at high doses of up to 100 mg/egg did not produce incorrect calls (the only equivocal experiment with eugenol used a top dose of 0.6 mg/egg). The day-9 protocol was also tested with three MPs (eugenol, *p*-nitrophenol, resorcinol) (Supplementary Figure S32B and D and S33D, available at *Mutagenesis* Online). Eugenol produced a clear negative call (Supplementary Figure S32B, available at *Mutagenesis* Online) in the same laboratory, which had classified the compound as equivocal in the main study (Supplementary Figures S19C and D and S32A, available at *Mutagenesis* Online). Resorcinol and *p*-nitrophenol reproduced the negative call from coded testing (summarised in Table 3). Therefore, the HET-MN protocol was amended with the recommendation to further investigate compounds, which induce strong toxicity already at low doses of <1 mg/egg—without having an impact on MN frequency—with the ‘day-9 protocol’.

#### Proof of test chemical’s bioavailability

The PCE/NCE ratio had been introduced at an early stage of the HET-MN development as an additional indicator for the test chemical’s bioavailability (11). A systematic analysis of the validation data set showed this parameter to be quite stable across all three laboratories even if accompanied by clear indications of genotoxicity or general toxicity (18). In consequence, the PCE/NCE ratio was not considered sufficiently sensitive to proof the bioavailability of a test chemical and is therefore no longer included in the HET-MN protocol.

#### Statistical analysis

A first PM (PM1) was introduced during development and optimisation phases (10), which combines a pre-defined threshold with the JT trend test to check for a monotonic increase of MN frequency. The latter is of special relevance in case of moderate increases that do not exceed the pre-defined threshold. A second PM (PM2) was developed in the initial phase of this joint project, before the start of the validation exercise (29). It is based on the UW test, which compares single- and pooled-dose groups against the SC, thereby being able to detect different types of dose–response curves. An analysis of the performance of both PMs based on the validation data set by Maul *et al.* (18) resulted in the recommendation to merge the statistical methods by combining the threshold procedure (PM1) with the UW test (PM2) in a final PM to identify increases of MN frequency of varying dose–response relationships. For transparency reasons, the outcomes of both PM1 and PM2 are indicated in the respective graphs (Figures 1–3 and Supplementary Figures S1–S33, available at *Mutagenesis* Online). None of the calls presented in this publication would change when applying the final PM.

### Strategic use of the HET-MN assay

The presence of MN in cultured cells has been reported as early as the 1960s (58) as an indicator for clastogenic and aneugenic effects (59). Meanwhile, the mechanistic relevance of MN formation for toxicological assessment is widely accepted as documented in respective OECD TGs (1, 7), supporting the assessment of chemicals in different regulatory sectors such as industrial chemicals (3), plant protection products (4), pharmaceuticals (60) and cosmetics (2).

The MNvit holds a central position *in vitro* test batteries (2, 3). Its position is supported by the assay’s good sensitivity (5). However, when the MNvit is combined in a battery approach, positive findings were observed, which disagreed with negative *in vivo* findings obtained with the same chemical (6). Despite their optimisation (see revised OECD testing Guidelines (1)), classical *in vitro* genotoxicity assays based on 2D cell cultures remain limited in mirroring the route of exposure and in showing an intrinsic xenobiotic metabolism, necessitating the use of an external metabolising system, two crucial aspects specified by current OECD TGs (1, 7). In consequence, follow-up testing is often performed using animal experiments, which are prohibited or restricted by a growing number of legislations across the globe (3, 61–63). Therefore, three-dimensional test systems have been introduced into genotoxicity testing (9), including the HET-MN (10), to fill a toolbox to further investigate positive findings from initial testing without animal experiments.

With the new assays, which utilise test systems with clear intrinsic metabolic capacity, the three routes of exposure can be addressed. For the dermal route, reconstructed skin (RS) tissues have been employed to develop the RS Comet assay (64) and the RS MN assay (65), which both successfully passed validation exercises recently (66, 67). In addition, proof-of-concept studies have been presented to address the inhalative route by combining EpiAirway™ tissues (MatTek) with the comet assay (68), while spheroids from a human liver cancer cell line, HepG2 cells, were used for the evaluation of MN to reflect genotoxic effects following exposures via the oral route (69).

The HET-MN is considered a good candidate to complement the *in vitro* genotoxicity toolbox. In contrast to 2D cell cultures, chicken eggs are characterised by a clear metabolic capacity, which is mediated by functional cell units in the yolk sac membrane, which in turn are in close vicinity to focal points of erythrocytes maturation, and in the developing liver. The metabolic capacity of the chicken eggs has been proven by the correct prediction of 12 pro-mutagens during development and validation phases. Furthermore, the developing chicken egg is a fast-cycling test system during the developmental stage at which the HET-MN is being performed, i.e., the number of erythrocytes per blood volume increases exponentially, while the same holds true for the blood volume. Moreover, erythrocytes bearing MN are not eliminated as the spleen is not yet functioning at this early developmental stage, and erythrocytes are almost the only cell type circulating in the blood at this stage. These aspects are supposed to establish the basis for the very good predictivity of the HET-MN in the validation exercise (specificity 98%, sensitivity 84%, overall accuracy 91%). In addition, during the development and optimisation phases of the assay (10–14), 21 chemicals had been tested and predicted correctly (Table 4).

**Table 4.** Chemicals tested non-coded during the development and optimisation phases of the HET-MN before the validation exercise

Chemical	CAS no.	Category	Lab C	Lab D	References
2,4-Dichlorophenol	120-83-2	MP	Neg	Neg	(10)
4-Chloroaniline	106-47-8	TP	Pos	Pos	(10)
Acrylamide	79-06-1	TP	Pos	Pos	(10)
Ampicillin sodium	69-52-3	TN	Neg	Neg	(10)
Azo rubin S	13613-55-3	TN	Neg	Neg	(10)
Cadmium chloride	10108-64-2	TP	—	Pos	(14)
Carbendazim	10605-21-7	TP	Pos	Pos	(10)
Cyclophosphamide	50-18-0	TP	Pos	Pos	(11, 12, 14)
Cytosine arabinoside	147-94-4	TP	—	Pos	(14)
7,12-Dimethyl-benz[*a*]anthracene	57-97-6	TP	Pos	Pos	(10–12)
Potassium chromate	7789-00-6	TP	—	Pos	(14)
Isophorone	78-59-1	TN	Neg	Neg	(10)
Methotrexate	59-05-2	TP	Pos	Pos	(10, 14)
Methyl methanesulfonate	66-27-3	TP	—	Pos	(11)
Mitomycin C	50-07-7	TP	—	Pos	(11)
*N*-Nitrosodiethanolamine	1116-54-7	TN	—	Neg	(13)
*N*-Nitrosodiethylamine	55-18-5	TP	Pos	Pos	(10, 13)
*N*-Nitrosodimethylamine	62-75-9	TP	—	Pos	(13)
Orange G	1936-15-8	TN	Neg	Neg	(10)
Starch	9005-25-8	TN	—	Neg	(14)
Vinorelbine tartrate	125317-39-7	TP	Pos	Pos	(10)

Neg, negative study; Pos, positive study.

Since 2018, the HET-MN is mentioned in the Notes of Guidance of the EU Scientific Committee on Consumer Safety (2). The independent expert panel of the European Commission, mandated to ensure the safe use of consumer products, suggested the HET-MN as one assay within a toolbox for a further evaluation of positive outcomes from initial testing with the MNvit (1) in a weight-of-evidence approach. The validation data set is supposed to build the basis for further regulatory acceptance.

## Conclusion

1. The performance of the assay to correctly predict the expected genotoxic effects of a difficult set of coded chemicals was very good, providing a sensitivity of 84% and a specificity of 98%. The overall accuracy was 91%.
2. The within-laboratory reproducibility was very good with 92%, as was the between-laboratory reproducibility with 87%, which was based on the final calls.
3. The validation proved the suitability of fertilised chicken eggs for genotoxicity assessment as shown by the reproducibly low background DNA damage and the intrinsic metabolic capacity being sufficient to toxify pro-mutagens.
4. The HET-MN has gained regulatory acceptance from the EU Scientific Committee on Consumer Safety, which now suggests the assay as a follow-up to help address positive findings from the initial testing with the classical *in vitro* test battery.
5. The HET-MN protocol has been finalised and comprises the test protocol, cell analysis, validity criteria and the evaluation of results based on statistical and biological relevance.

## Supplementary data

Supplementary data are available at *Mutagenesis* Online.

**Figures S1-31. Graphical overview of validation results**. For each compound, the lab-specific data of at least two experiments are shown. The FT-transformed MN rate (circles; left axis) and the egg viability (triangles; right axis) are given in relation to the different treatments. Filled triangles indicate viabilities below 40%. MN data are given as mean ± standard deviation and as raw data (small cross symbols). Dotted horizontal lines refer to the MN rate and indicate the upper acceptance limit for the solvent control (SC), and the lower acceptance limit for the positive control (PC). MN data were tested for an increase above the threshold (Th), i.e., the threshold for a positive call and for a linear trend using the Jonckheere-Terpstra (JT) test (prediction model 1, PM1). MN data were also analysed using the Umbrella-Williams (UW) procedure (PM2). Finally, the result of the expert judgment (EJ) is indicated. For each test, a positive outcome is indicated by a crossed check box at the top of graphs. Filled circles above the *x*-axis (individual or linked) indicate single or pooled dose groups for which the UW test indicated a statistically significant increase; circles with a black outline circles indicate single or pooled dose groups with the smallest significant *p*-value. The used solvents (DMSO, HSO, IPM) are indicated in the low right, adjacent labels “S” and “R” indicate single and repeated dose.

**Figures S32-33. Comparison of the standard protocol with the “day-9 protocol” using benzo[*a*]pyrene**. Eggs were either treated on day 8 of egg development (left) or on day 9 (right) with the indicated doses and chemicals while sampling was done on day 11. For further graphic details, see legend of Figure 1.

**Figure S34. Egg viability of solvent and positive controls (SC, PC) in the three laboratories in the course of the validation study.** In phase I instead of cyclophosphamide (CP, white triangles) 7,12-dimethyl-benz[*a*]anthracene (DMBA, filled triangles) was partly used as PC.

**Table S1.** Literature information on the *in vitro* and *in vivo* genotoxicity of the 32 chemicals included in the final evaluation.

geab016_suppl_Supplementary_Figures_S1_S31

geab016_suppl_Supplementary_Figure_S32_S33

geab016_suppl_Supplementary_Figure_S34

geab016_suppl_Supplementary_Table_S1
