# Supplementary material for: The hen’s egg test for micronucleus induction (HET-MN): validation data set
Source: Mutagenesis. 2021 Jun 3;37(2):61–75. doi: 10.1093/mutage/geab016 (PMC9071061; doi:10.1093/mutage/geab016)
Supplement: geab016_suppl_Supplementary_Figures_S1_S31 [file geab016_suppl_supplementary_figures_s1_s31.pdf]

Figure S1

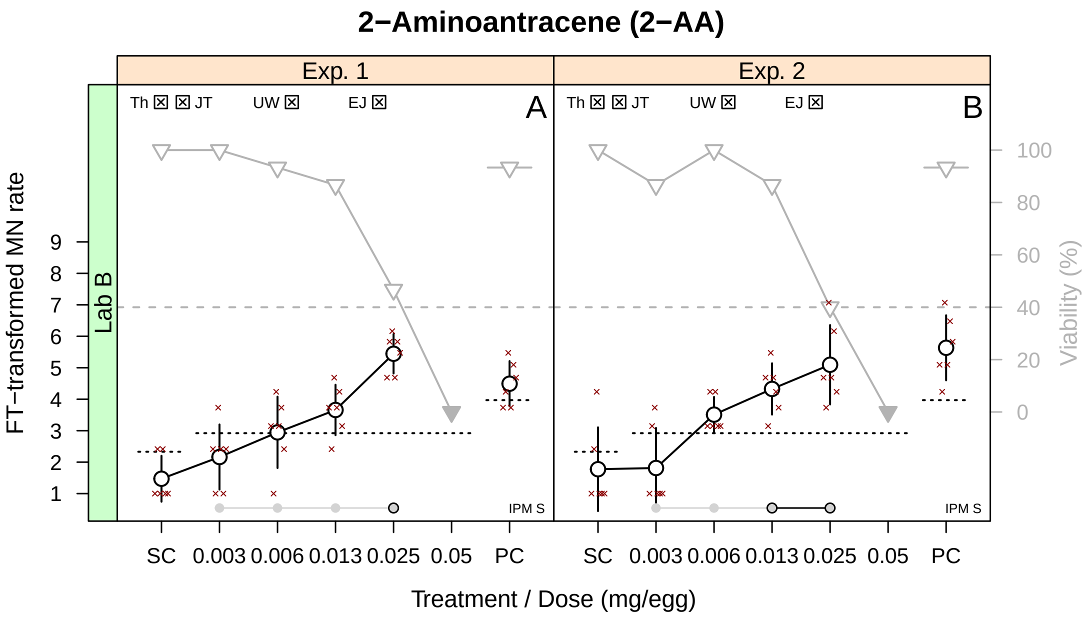

2−Aminoantracene (2−AA)
Exp. 1
Exp. 2
Lab B
Th ☒ ☒ JT
UW ☒
EJ ☒
A
B
IPM S
FT−transformed MN rate
Viability (%)
100
80
60
40
20
0
9
8
7
6
5
4
3
2
1
SC
0.003
0.006
0.013
0.025
0.05
PC
Treatment / Dose (mg/egg)

Figure S2

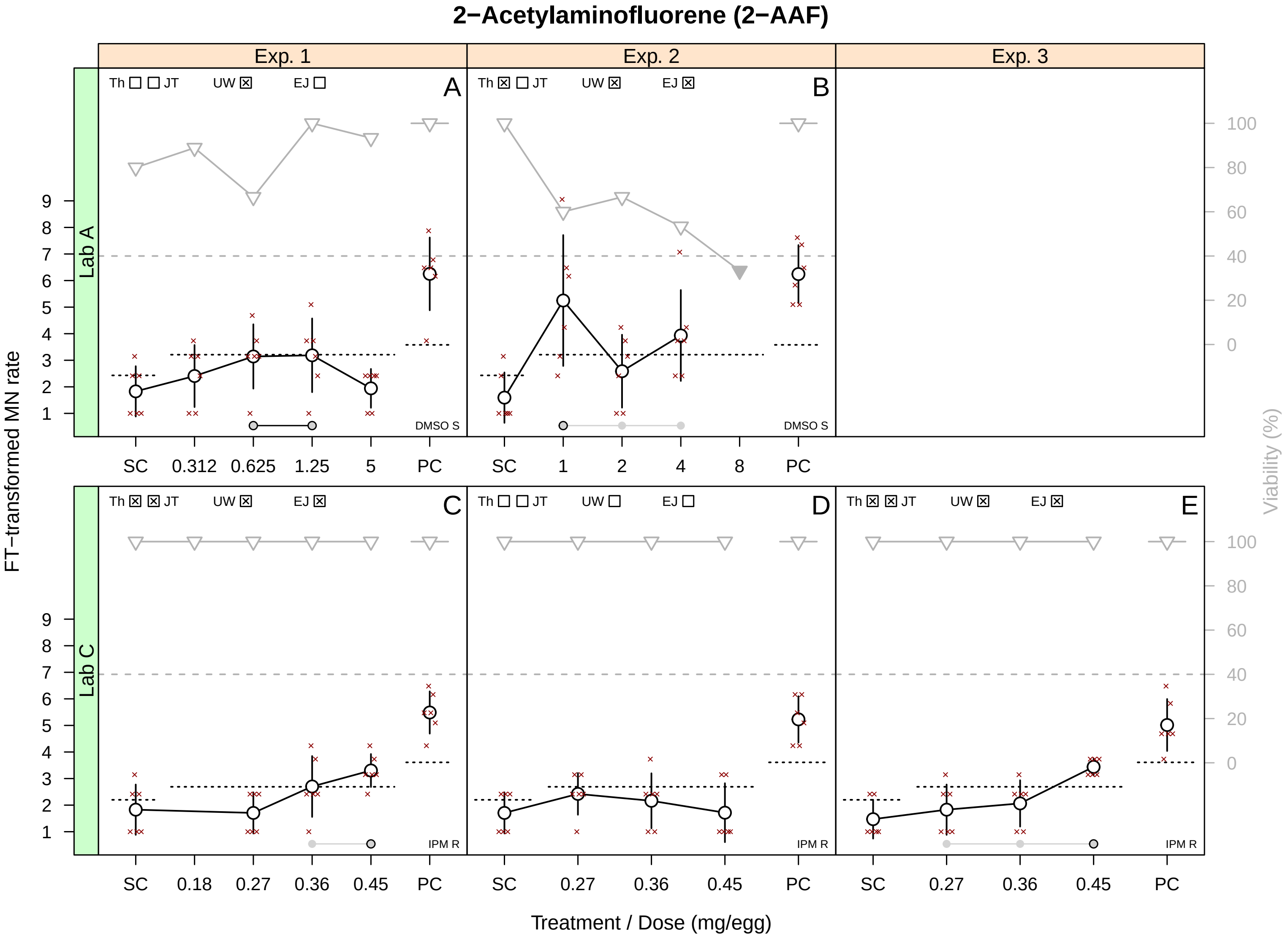
2−Acetylaminofluorene (2−AAF)
Exp. 1
Exp. 2
Exp. 3
Lab A
Lab C
A
B
C
D
E
Th JT UW EJ
DMSO S
IPM R
SC
0.312
0.625
1.25
5
PC
1
2
4
8
0.18
0.27
0.36
0.45
FT−transformed MN rate
Viability (%)
Treatment / Dose (mg/egg)

Figure S3

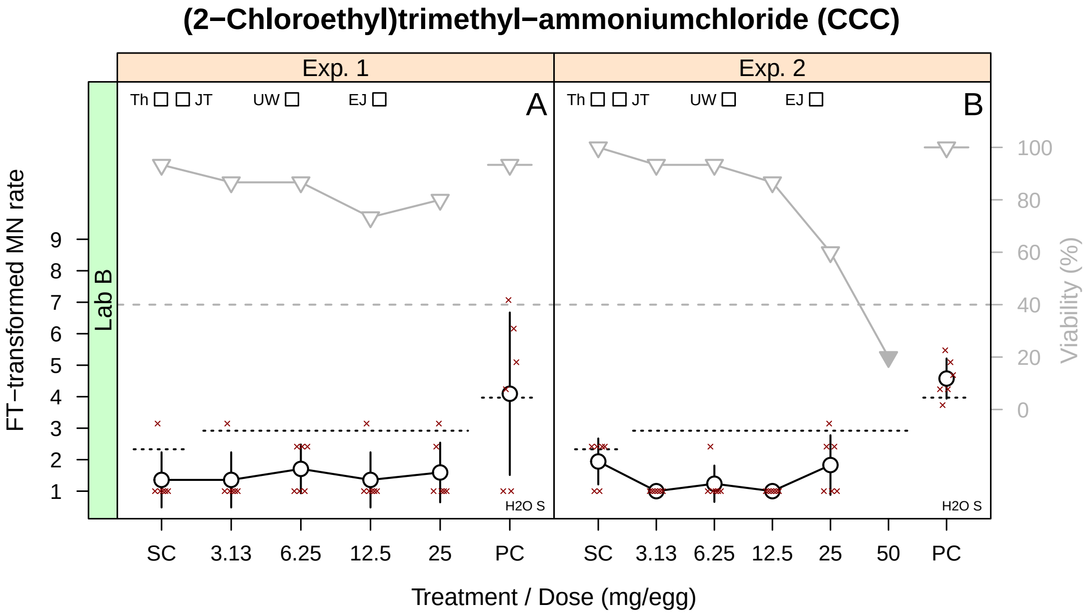
(2−Chloroethyl)trimethyl−ammoniumchloride (CCC)
Exp. 1
Exp. 2
Lab B
A
B
Th JT UW EJ
H2O S
FT−transformed MN rate
Viability (%)
SC 3.13 6.25 12.5 25 PC
SC 3.13 6.25 12.5 25 50 PC
Treatment / Dose (mg/egg)

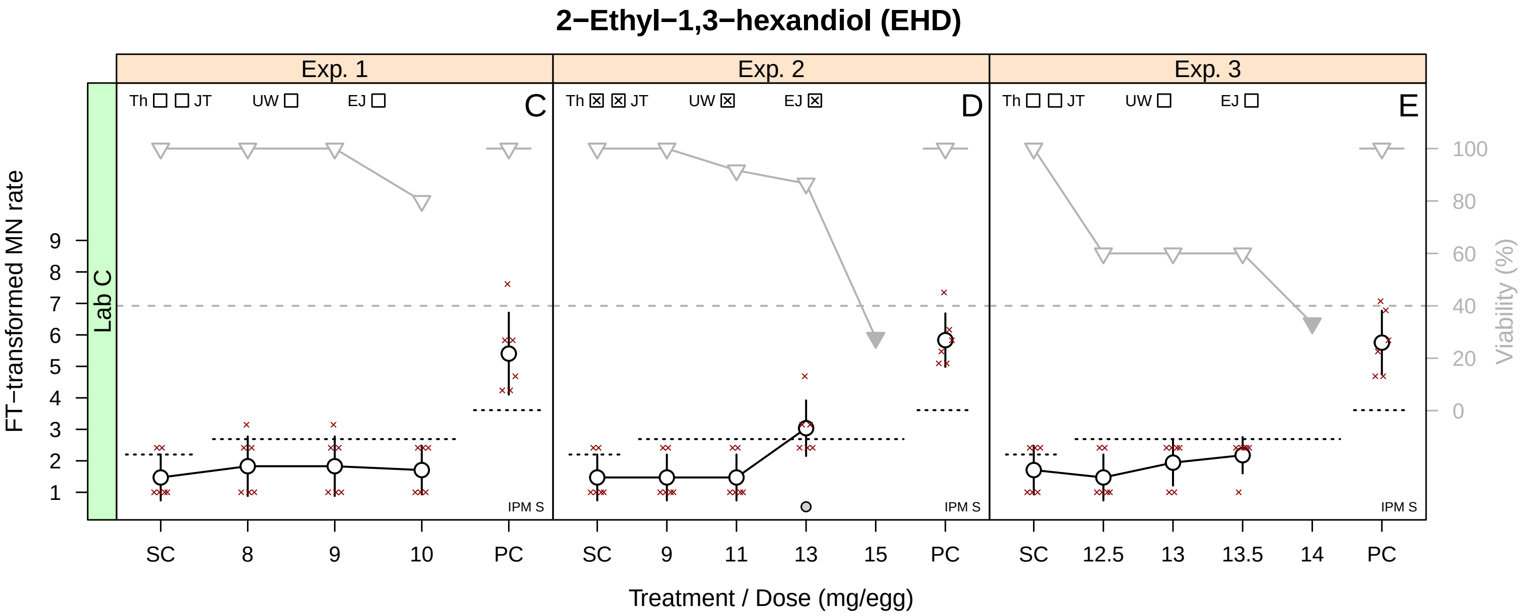
2−Ethyl−1,3−hexandiol (EHD)
Exp. 1
Exp. 2
Exp. 3
Lab C
C
D
E
Th JT UW EJ
IPM S
FT−transformed MN rate
Viability (%)
SC 8 9 10 PC
SC 9 11 13 15 PC
SC 12.5 13 13.5 14 PC
Treatment / Dose (mg/egg)

Figure S4

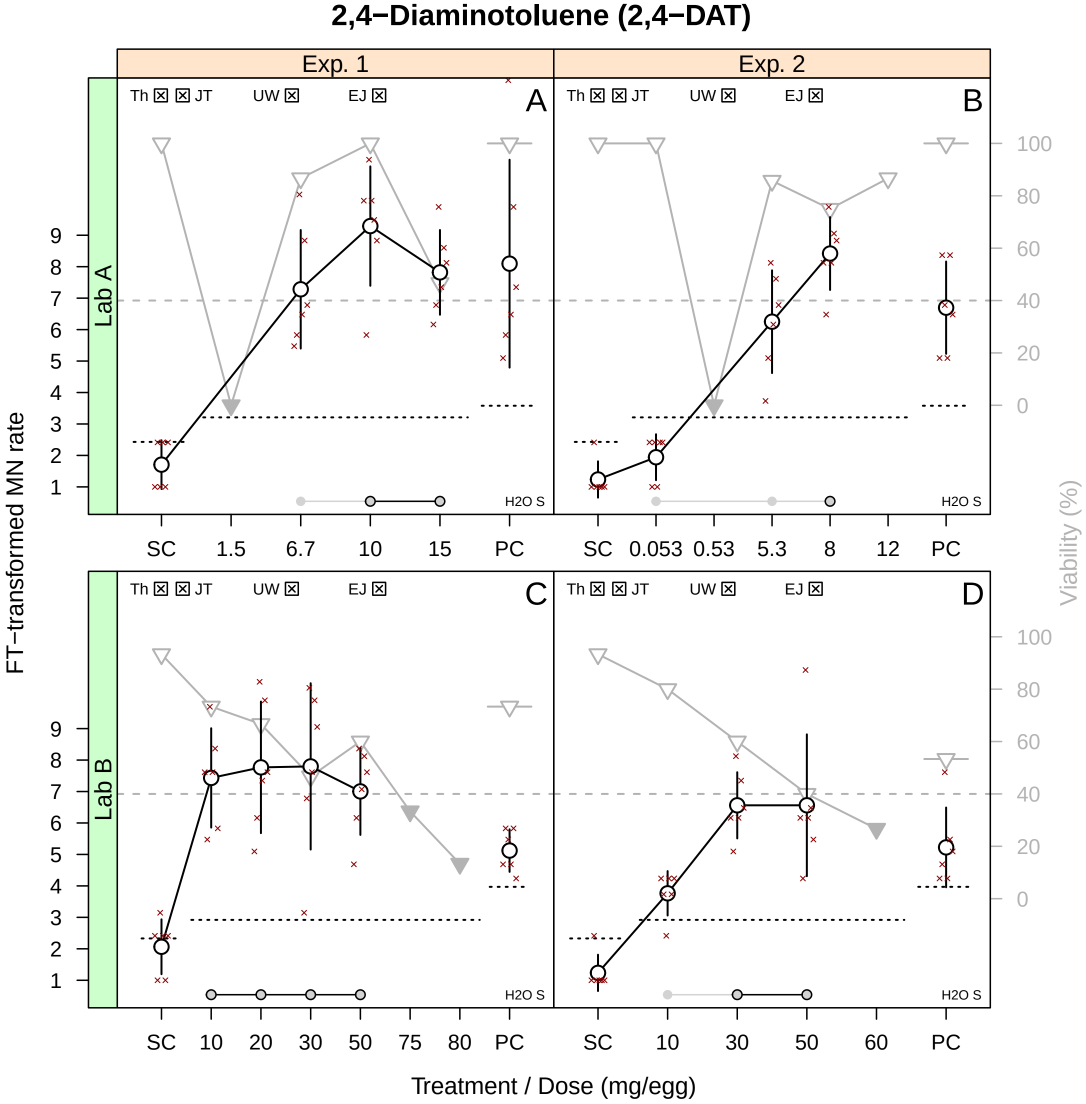
2,4−Diaminotoluene (2,4−DAT)
Exp. 1
Exp. 2
Lab A
Lab B
A
B
C
D
Th ☒ ☒ JT
UW ☒
EJ ☒
H2O S
SC
1.5
6.7
10
15
PC
0.053
0.53
5.3
8
12
20
30
50
75
80
60
FT−transformed MN rate
Viability (%)
Treatment / Dose (mg/egg)

Figure S5

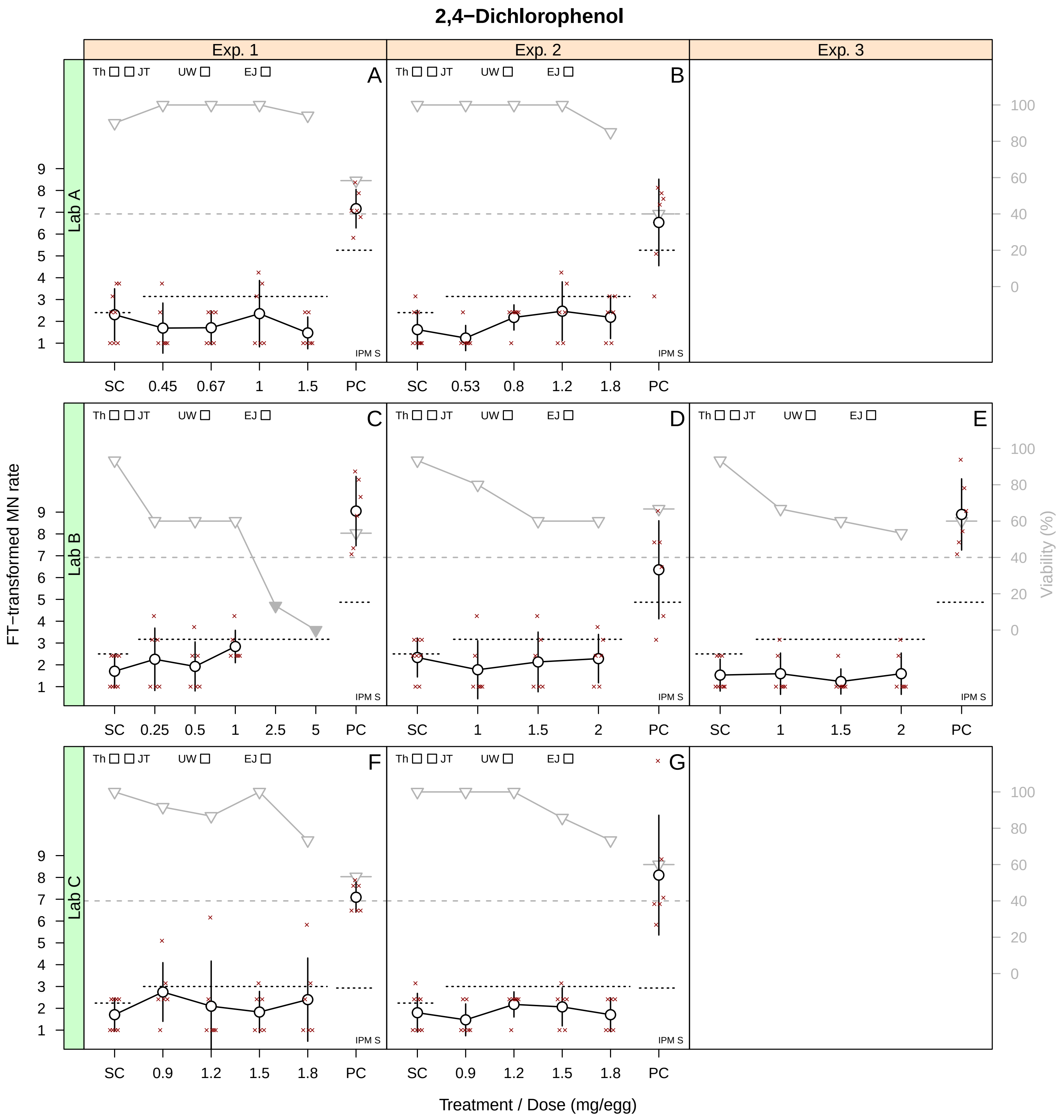
2,4−Dichlorophenol
Exp. 1
Exp. 2
Exp. 3
Lab A
Lab B
Lab C
A
B
C
D
E
F
G
Th
JT
UW
EJ
IPM S
SC
0.45
0.67
1
1.5
PC
0.53
0.8
1.2
1.8
0.25
0.5
2.5
5
2
0.9
FT−transformed MN rate
Viability (%)
Treatment / Dose (mg/egg)

Figure S6

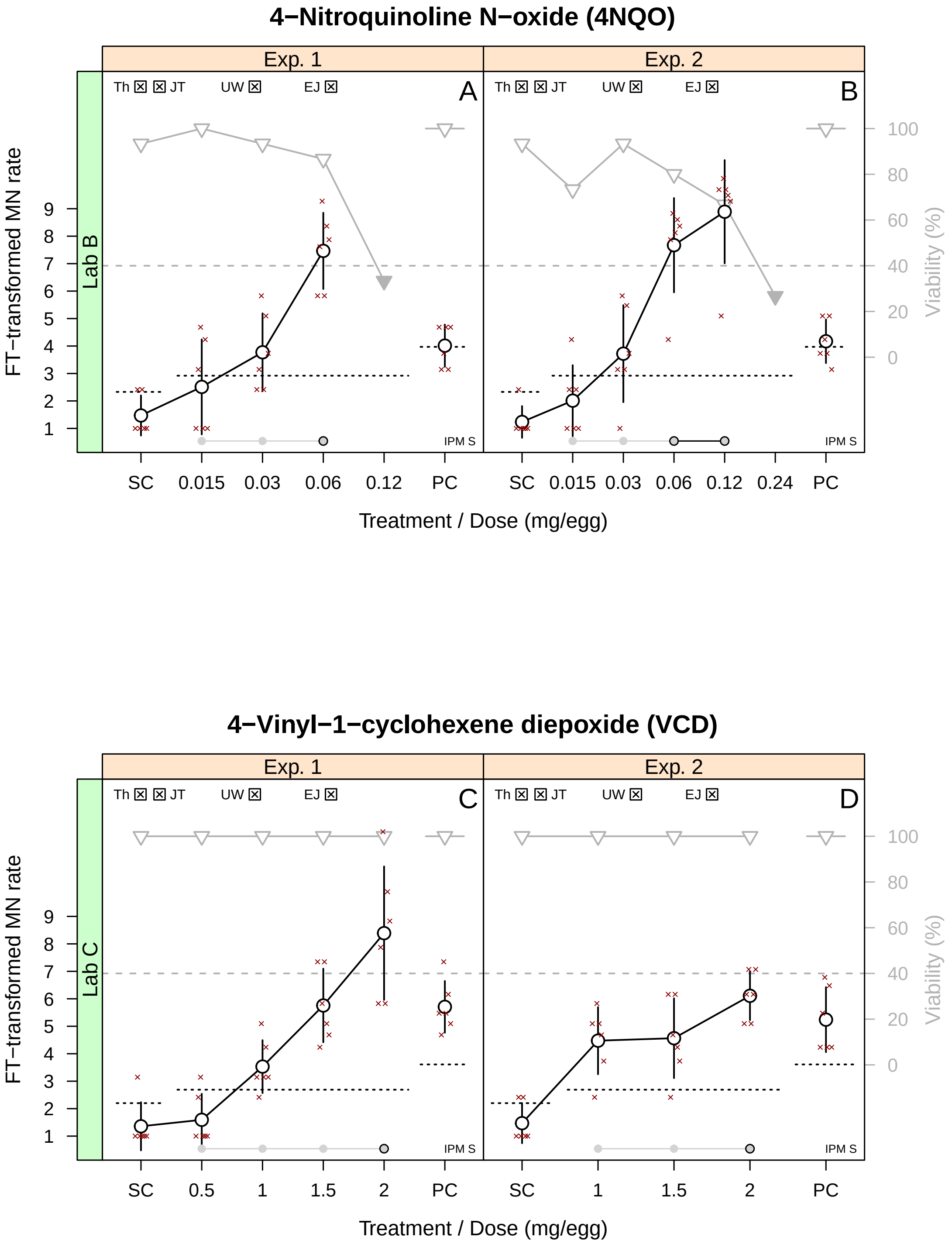
4−Nitroquinoline N−oxide (4NQO)
Exp. 1
Exp. 2
A
B
Lab B
FT−transformed MN rate
Viability (%)
IPM S
SC 0.015 0.03 0.06 0.12 PC
SC 0.015 0.03 0.06 0.12 0.24 PC
Treatment / Dose (mg/egg)
4−Vinyl−1−cyclohexene diepoxide (VCD)
C
D
Lab C
SC 0.5 1 1.5 2 PC
SC 1 1.5 2 PC
Treatment / Dose (mg/egg)

Figure S7

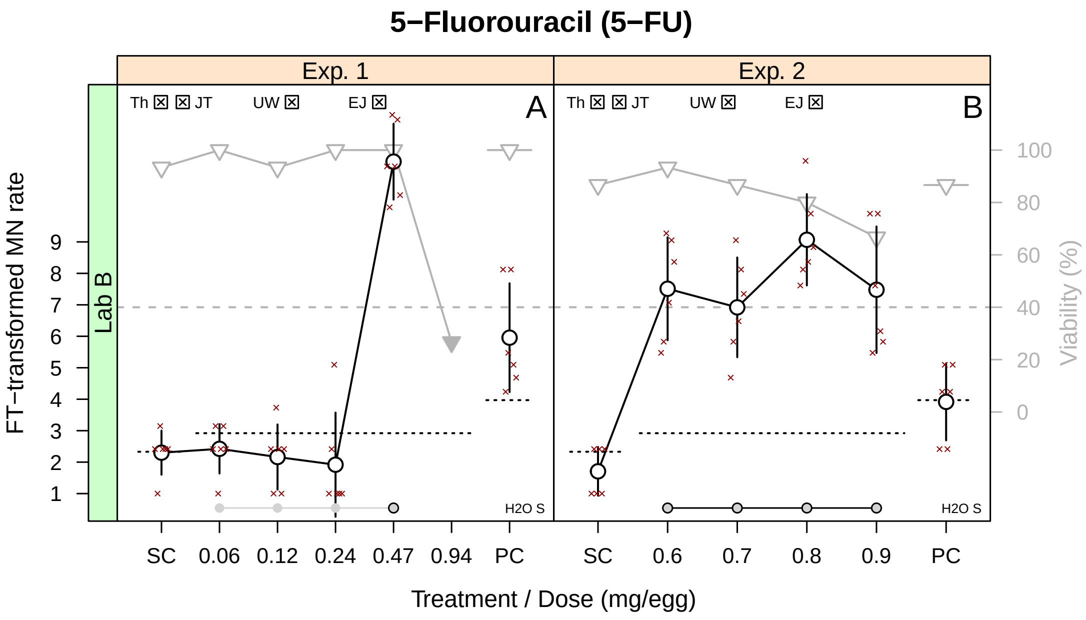
5−Fluorouracil (5−FU)
Exp. 1
Exp. 2
Lab B
Th ☒ ☒ JT
UW ☒
EJ ☒
A
B
H2O S
FT−transformed MN rate
Viability (%)
Treatment / Dose (mg/egg)
SC
0.06
0.12
0.24
0.47
0.94
PC
0.6
0.7
0.8
0.9
1
2
3
4
5
6
7
8
9
0
20
40
60
80
100

Figure S8

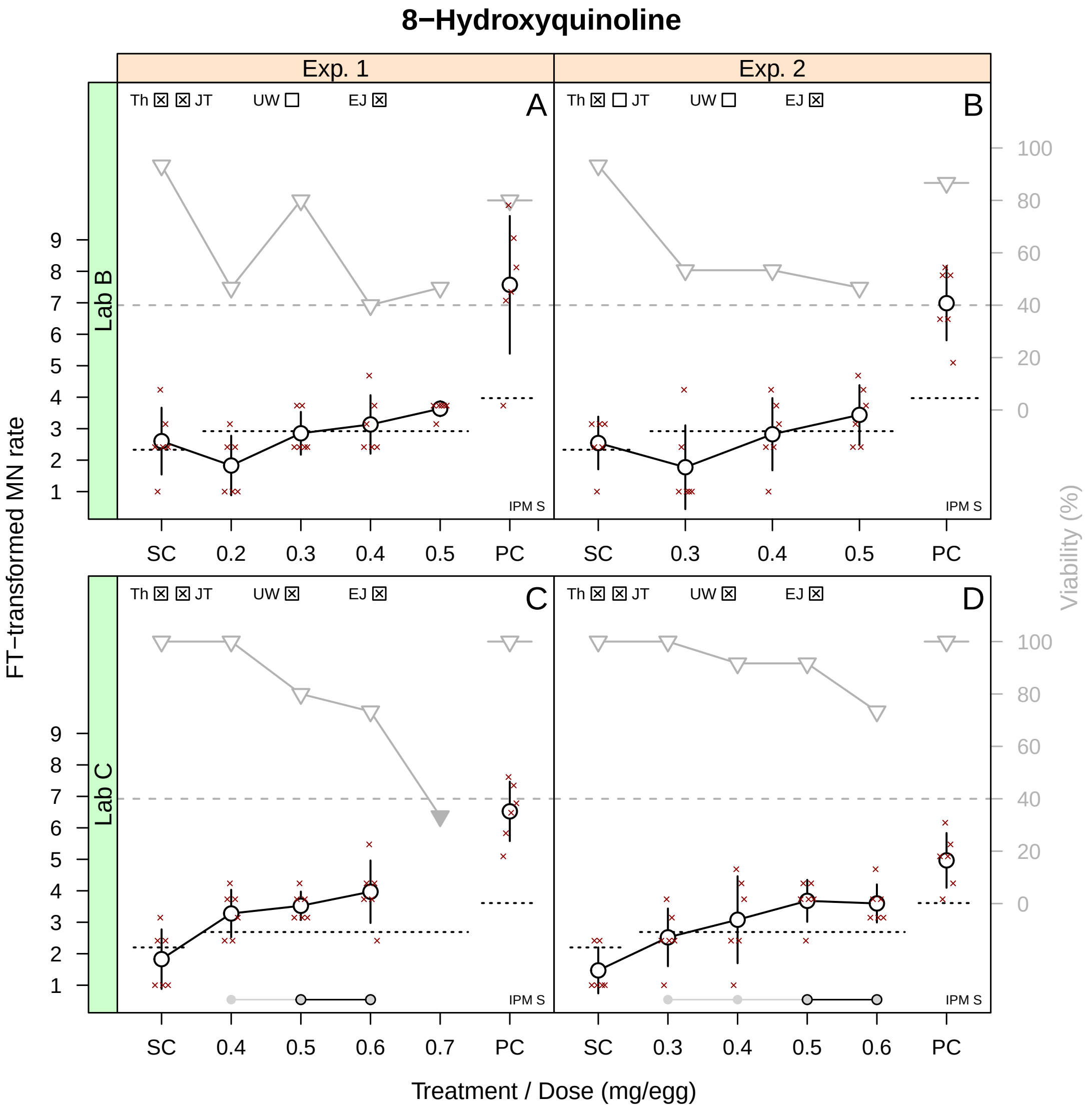

8−Hydroxyquinoline
Exp. 1
Exp. 2
Lab B
Lab C
A
B
C
D
Th ☒ ☒ JT
UW ☐
EJ ☒
Th ☒ ☐ JT
UW ☒
IPM S
FT−transformed MN rate
Viability (%)
SC
0.2
0.3
0.4
0.5
0.6
0.7
PC
Treatment / Dose (mg/egg)

Figure S9

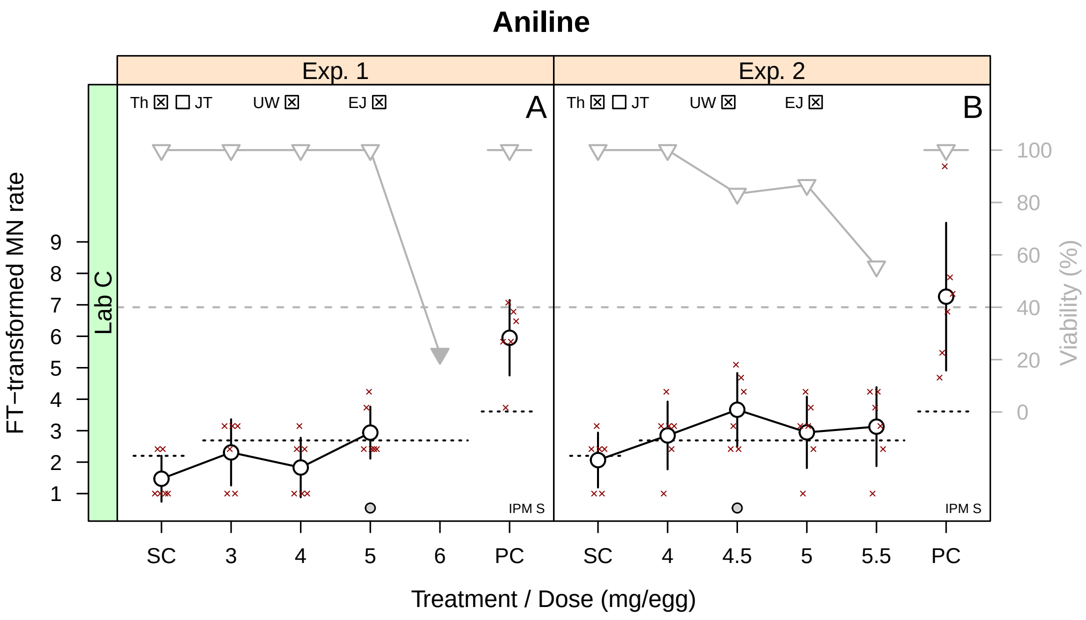

Aniline
Exp. 1
Exp. 2
Lab C
Th ☒ ☐ JT
UW ☒
EJ ☒
A
B
IPM S
FT−transformed MN rate
Viability (%)
SC
3
4
5
6
PC
4.5
5.5
Treatment / Dose (mg/egg)

Figure S10

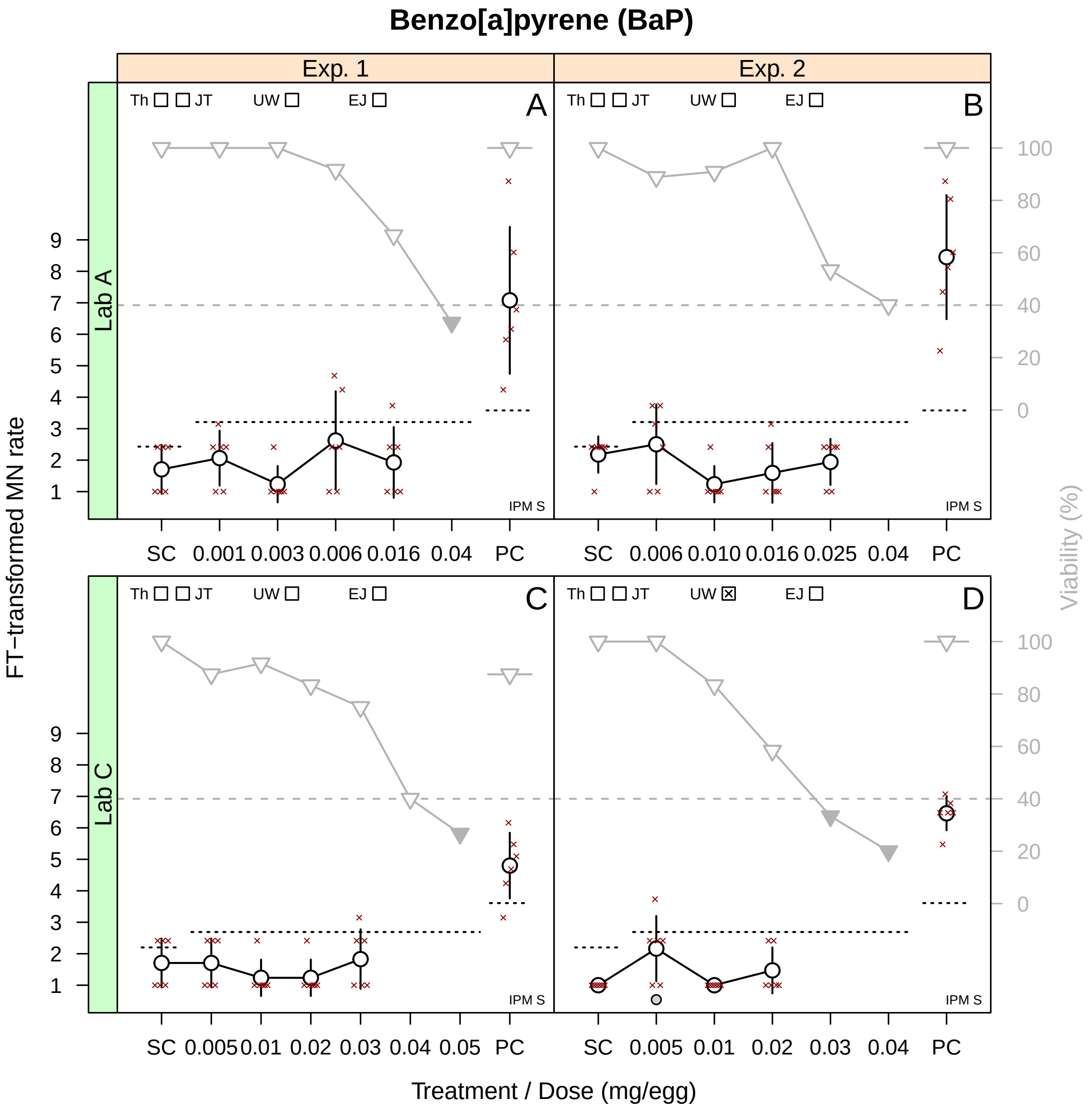
Benzo[a]pyrene (BaP)
Exp. 1
Exp. 2
Lab A
Lab C
A
B
C
D
Th
JT
UW
EJ
IPM S
FT−transformed MN rate
Viability (%)
SC 0.001 0.003 0.006 0.016 0.04 PC
SC 0.006 0.010 0.016 0.025 0.04 PC
SC 0.005 0.01 0.02 0.03 0.04 0.05 PC
SC 0.005 0.01 0.02 0.03 0.04 PC
Treatment / Dose (mg/egg)

Figure S11

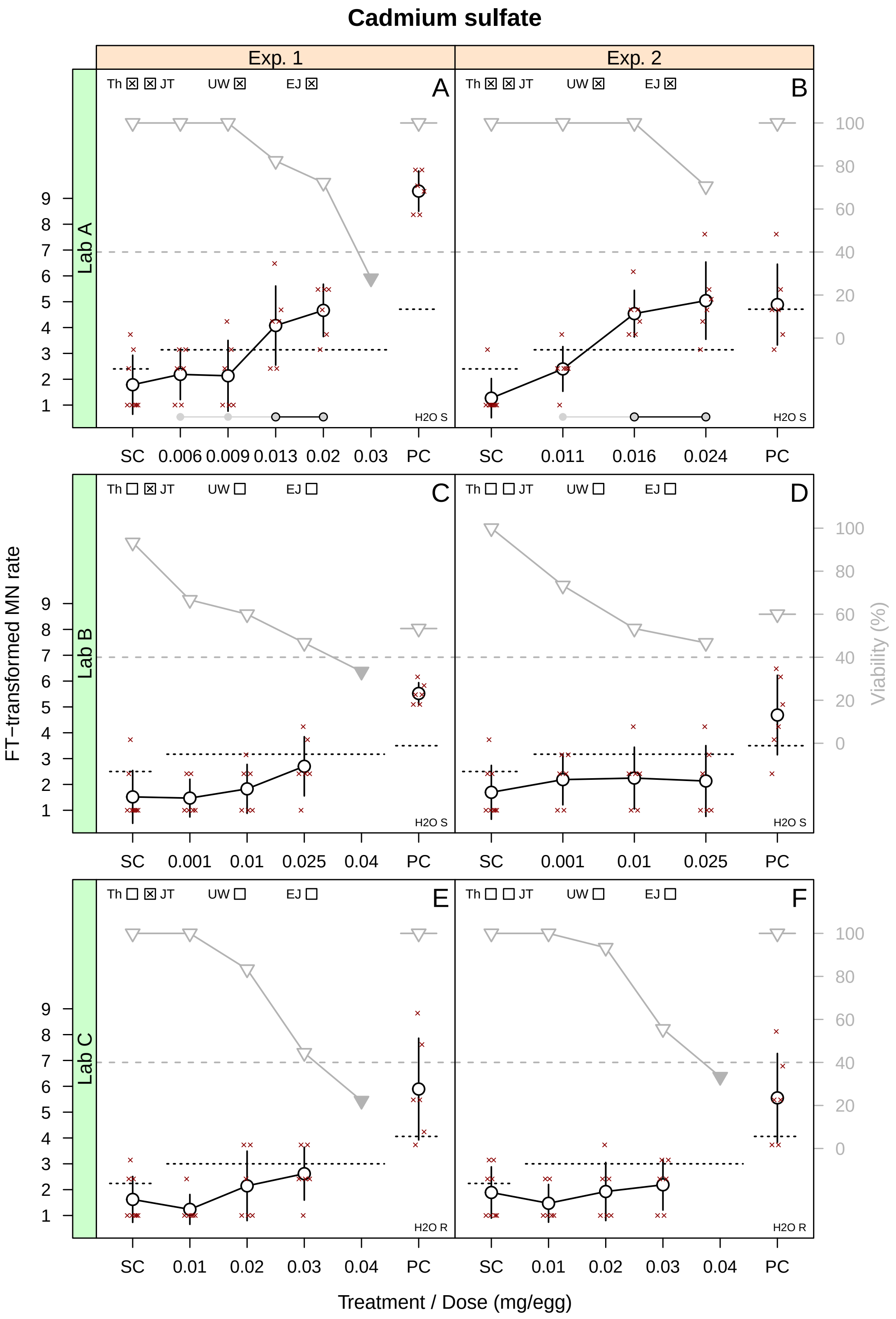
Cadmium sulfate
Exp. 1
Exp. 2
Lab A
Lab B
Lab C
A
B
C
D
E
F
Th JT UW EJ
H2O S
H2O R
SC 0.006 0.009 0.013 0.02 0.03 PC
SC 0.011 0.016 0.024 PC
SC 0.001 0.01 0.025 0.04 PC
SC 0.001 0.01 0.025 PC
SC 0.01 0.02 0.03 0.04 PC
FT−transformed MN rate
Viability (%)
Treatment / Dose (mg/egg)

Figure S12

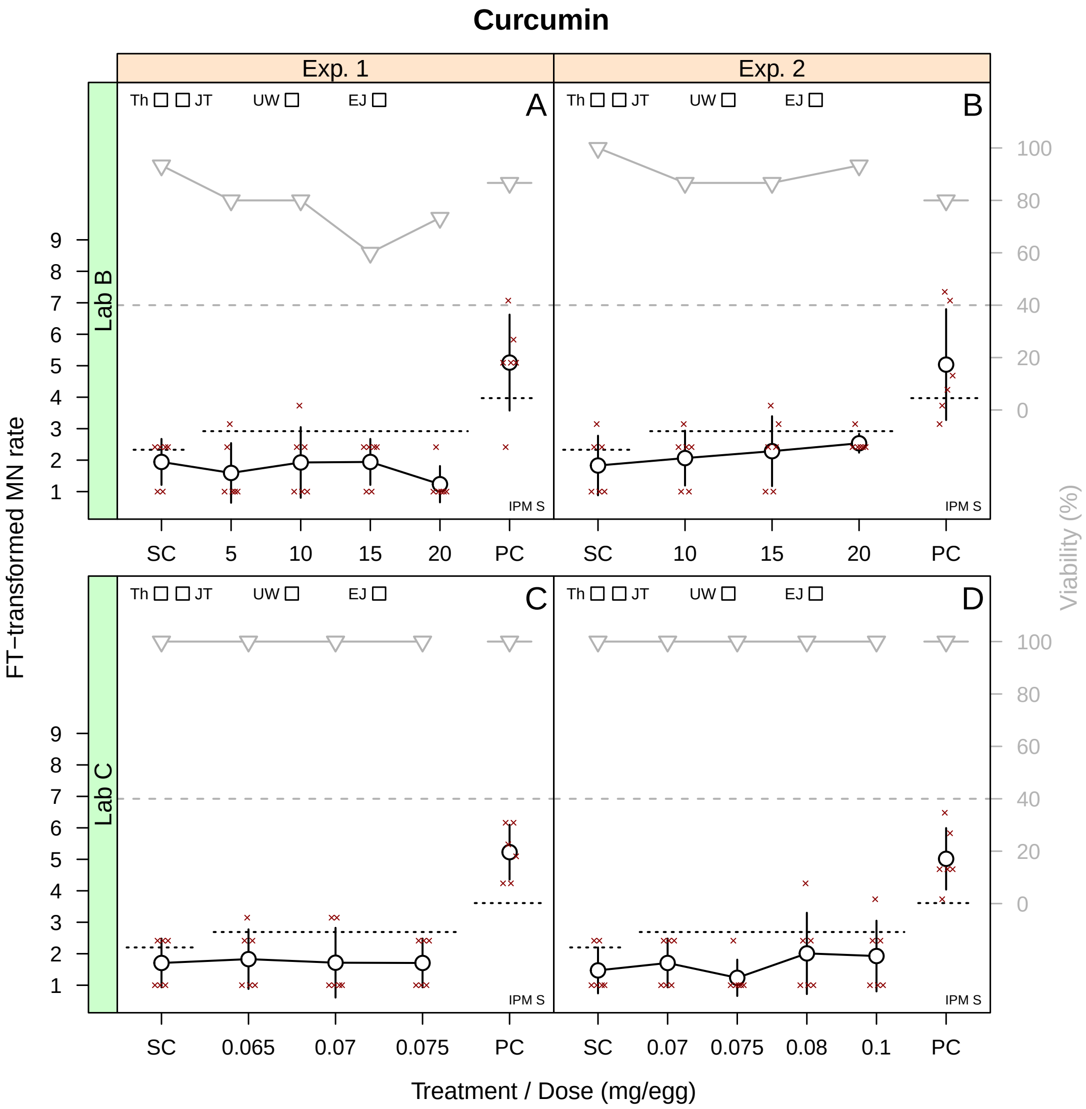

Curcumin
Exp. 1
Exp. 2
Lab B
Lab C
A
B
C
D
Th
JT
UW
EJ
IPM S
FT−transformed MN rate
Viability (%)
SC
5
10
15
20
PC
0.065
0.07
0.075
0.08
0.1
Treatment / Dose (mg/egg)

Figure S13

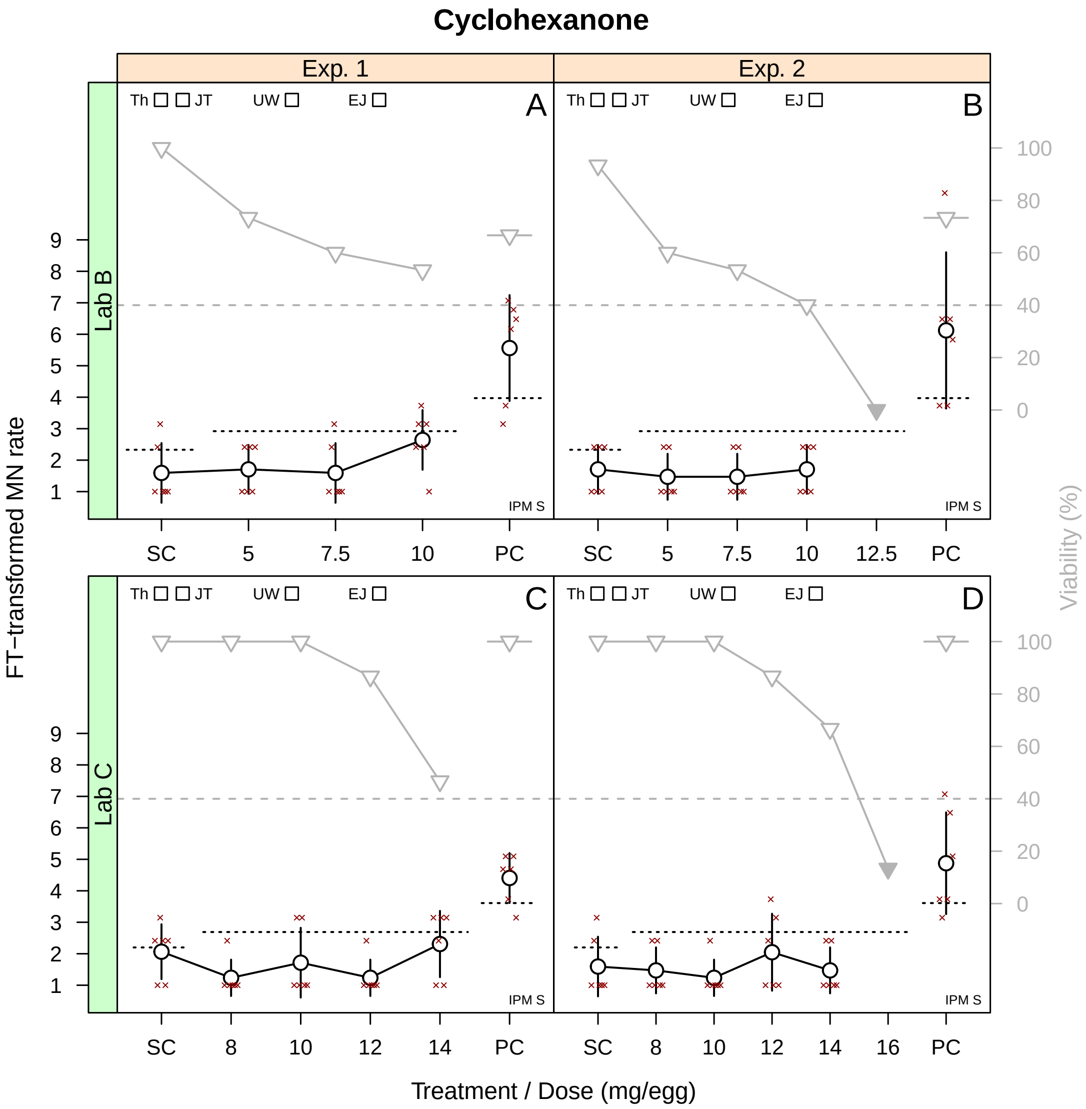
Cyclohexanone
Exp. 1
Exp. 2
Lab B
Lab C
A
B
C
D
Th
JT
UW
EJ
IPM S
FT−transformed MN rate
Viability (%)
Treatment / Dose (mg/egg)
SC
5
7.5
10
12.5
PC
8
12
14
16
100
80
60
40
20
0

Figure S14

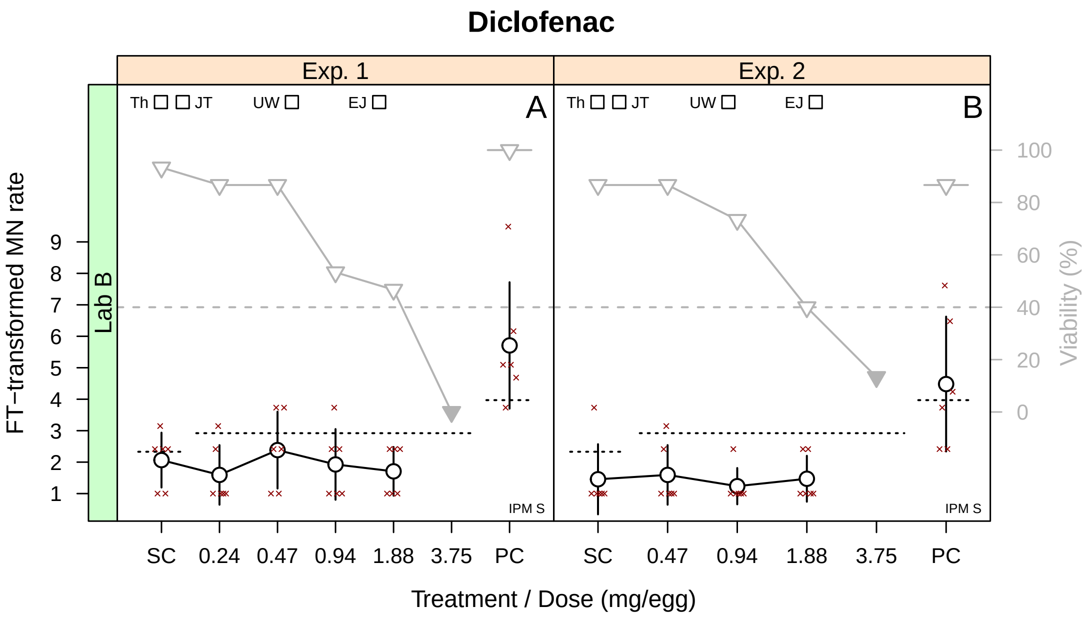

Diclofenac
Exp. 1
Exp. 2
Lab B
Th
JT
UW
EJ
A
B
IPM S
FT−transformed MN rate
Viability (%)
SC
0.24
0.47
0.94
1.88
3.75
PC
Treatment / Dose (mg/egg)

Figure S15

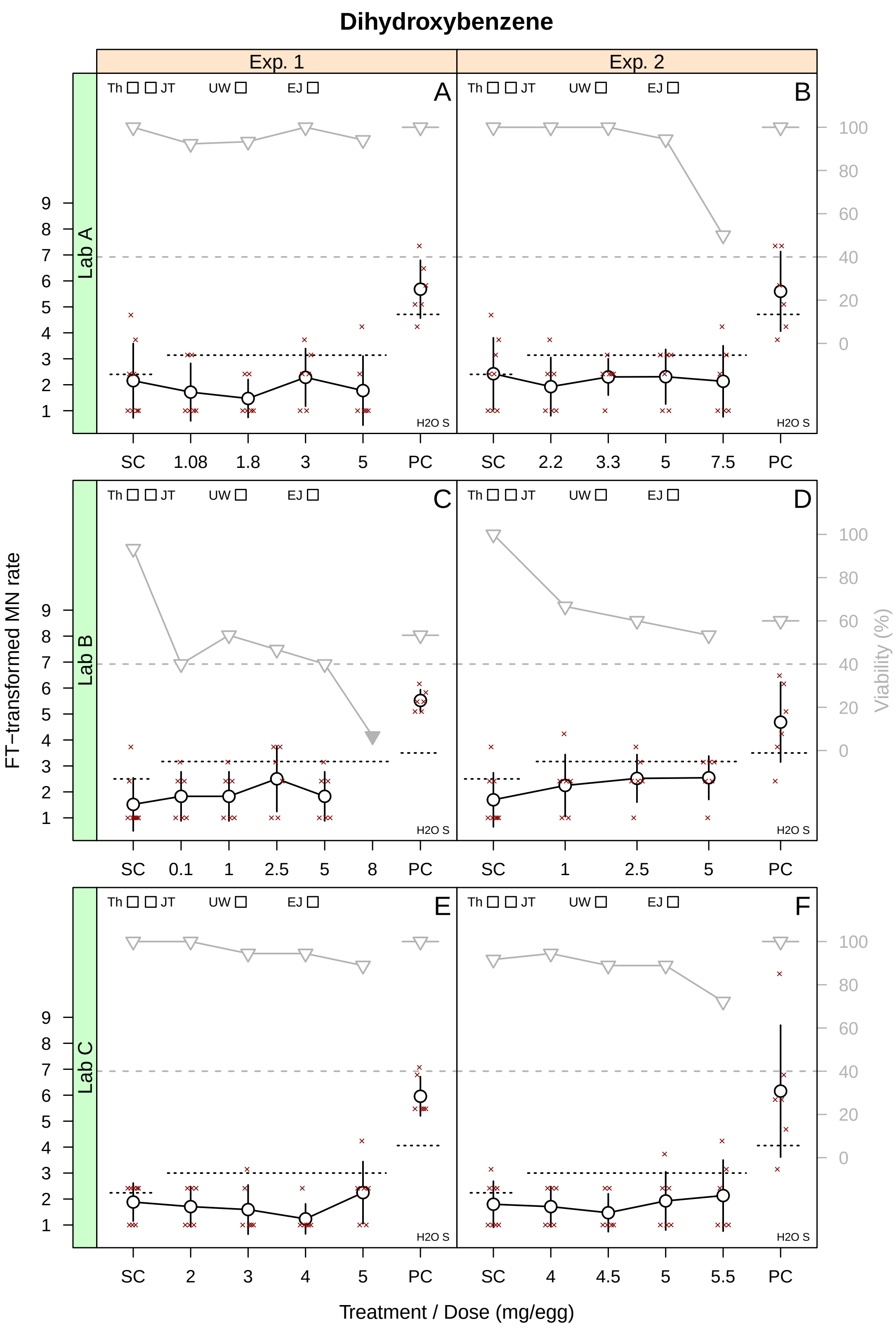
Dihydroxybenzene
Exp. 1
Exp. 2
Lab A
Lab B
Lab C
A
B
C
D
E
F
Th JT UW EJ
H2O S
SC 1.08 1.8 3 5 PC
SC 2.2 3.3 5 7.5 PC
SC 0.1 1 2.5 5 8 PC
SC 1 2.5 5 PC
SC 2 3 4 5 PC
SC 4 4.5 5 5.5 PC
FT−transformed MN rate
Viability (%)
Treatment / Dose (mg/egg)

Figure S16

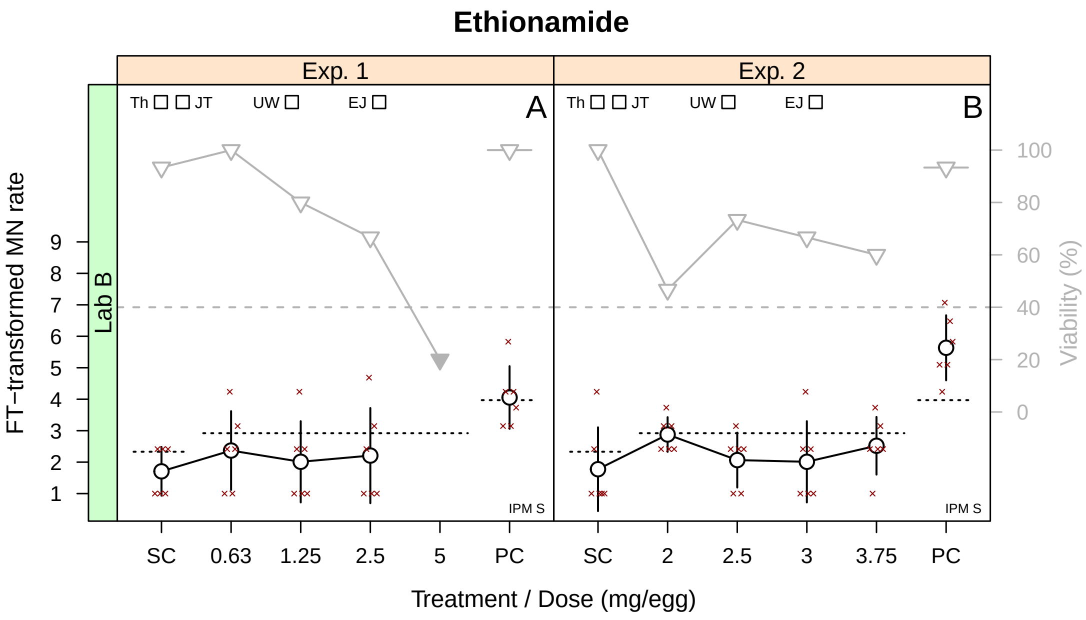
Ethionamide
Exp. 1
Exp. 2
Lab B
Th JT UW EJ
A
B
IPM S
FT−transformed MN rate
Viability (%)
1 2 3 4 5 6 7 8 9
0 20 40 60 80 100
SC 0.63 1.25 2.5 5 PC
SC 2 2.5 3 3.75 PC
Treatment / Dose (mg/egg)

Figure S17

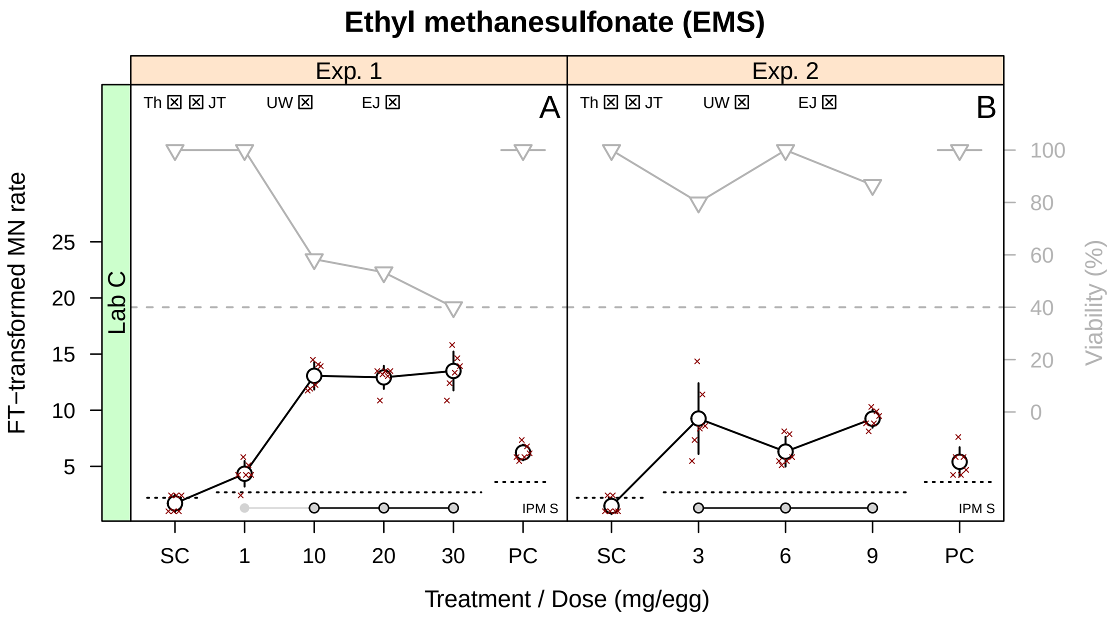

Ethyl methanesulfonate (EMS)
Exp. 1
Exp. 2
Lab C
A
B
Th ☒ ☒ JT
UW ☒
EJ ☒
IPM S
FT−transformed MN rate
Viability (%)
5
10
15
20
25
0
20
40
60
80
100
SC
1
10
20
30
PC
3
6
9
Treatment / Dose (mg/egg)

Figure S18

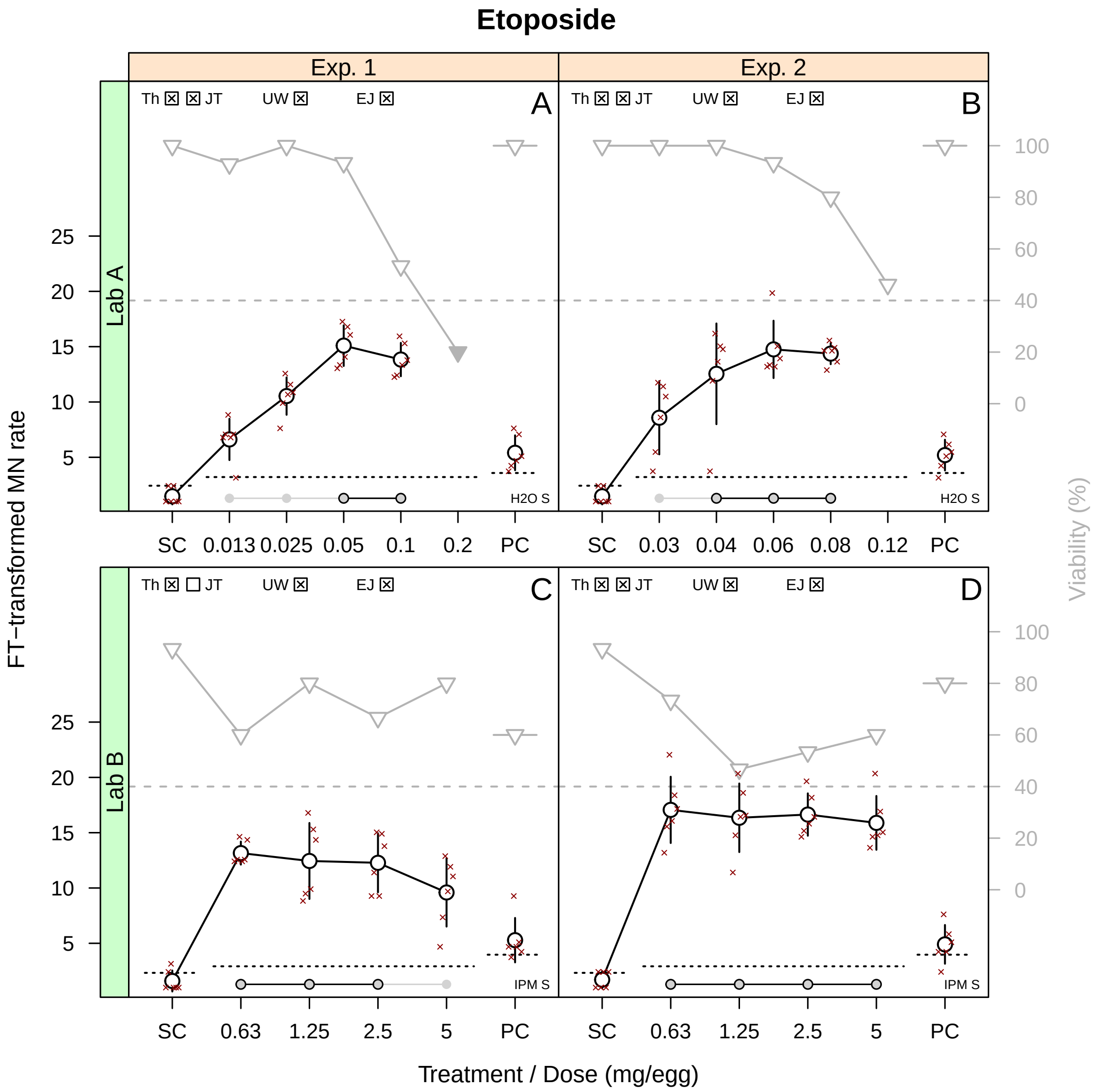

Etoposide
Exp. 1
Exp. 2
Lab A
Lab B
A
B
C
D
Th ☒ ☒ JT
UW ☒
EJ ☒
Th ☒ ☐ JT
H2O S
IPM S
SC
0.013
0.025
0.05
0.1
0.2
PC
0.03
0.04
0.06
0.08
0.12
0.63
1.25
2.5
5
FT−transformed MN rate
Viability (%)
Treatment / Dose (mg/egg)

Figure S19

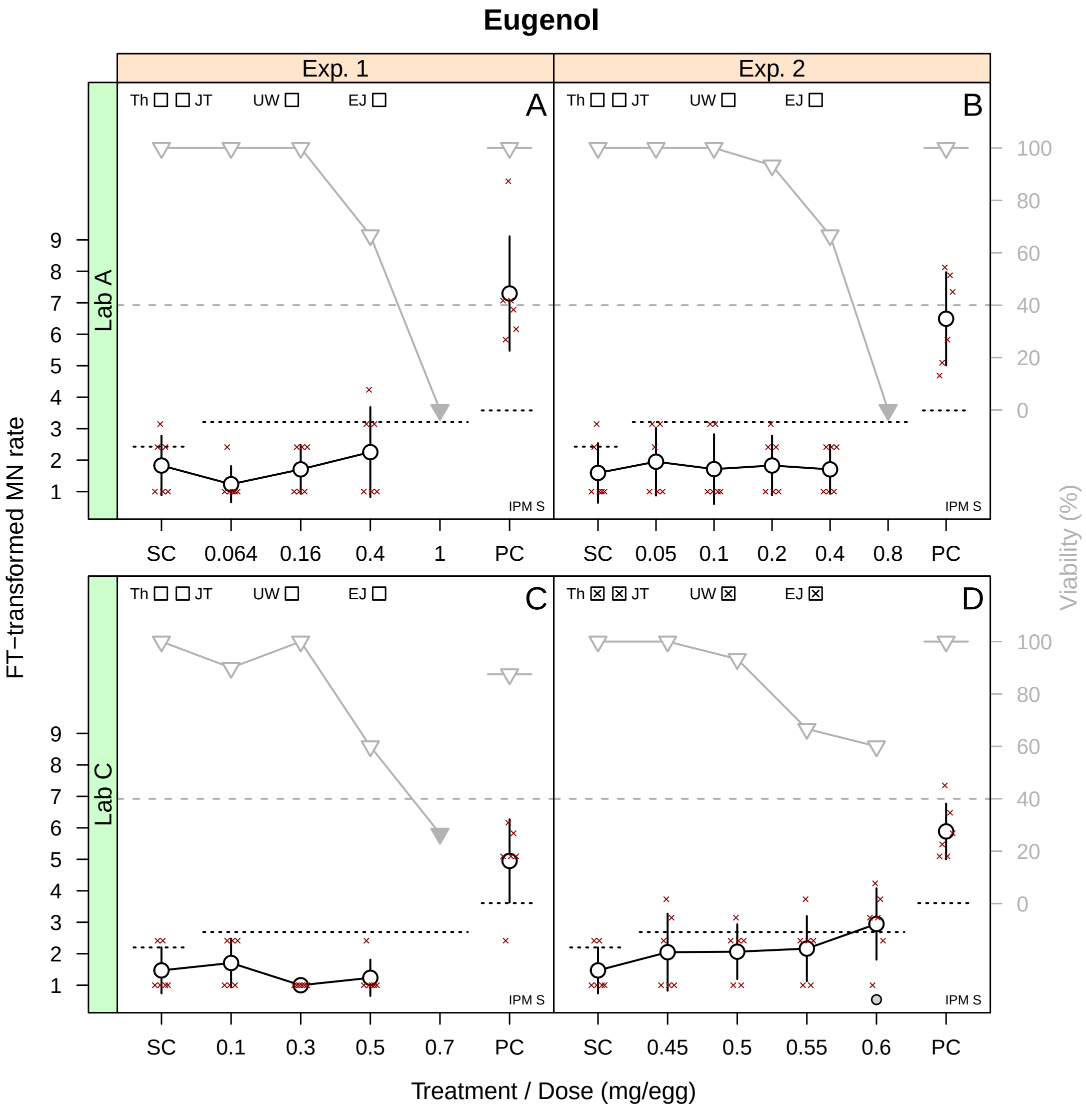

Eugenol
Exp. 1
Exp. 2
Lab A
Lab C
A
B
C
D
Th JT UW EJ
IPM S
FT−transformed MN rate
Viability (%)
1 2 3 4 5 6 7 8 9
0 20 40 60 80 100
SC 0.064 0.16 0.4 1 PC
SC 0.05 0.1 0.2 0.4 0.8 PC
SC 0.1 0.3 0.5 0.7 PC
SC 0.45 0.5 0.55 0.6 PC
Treatment / Dose (mg/egg)

Figure S20

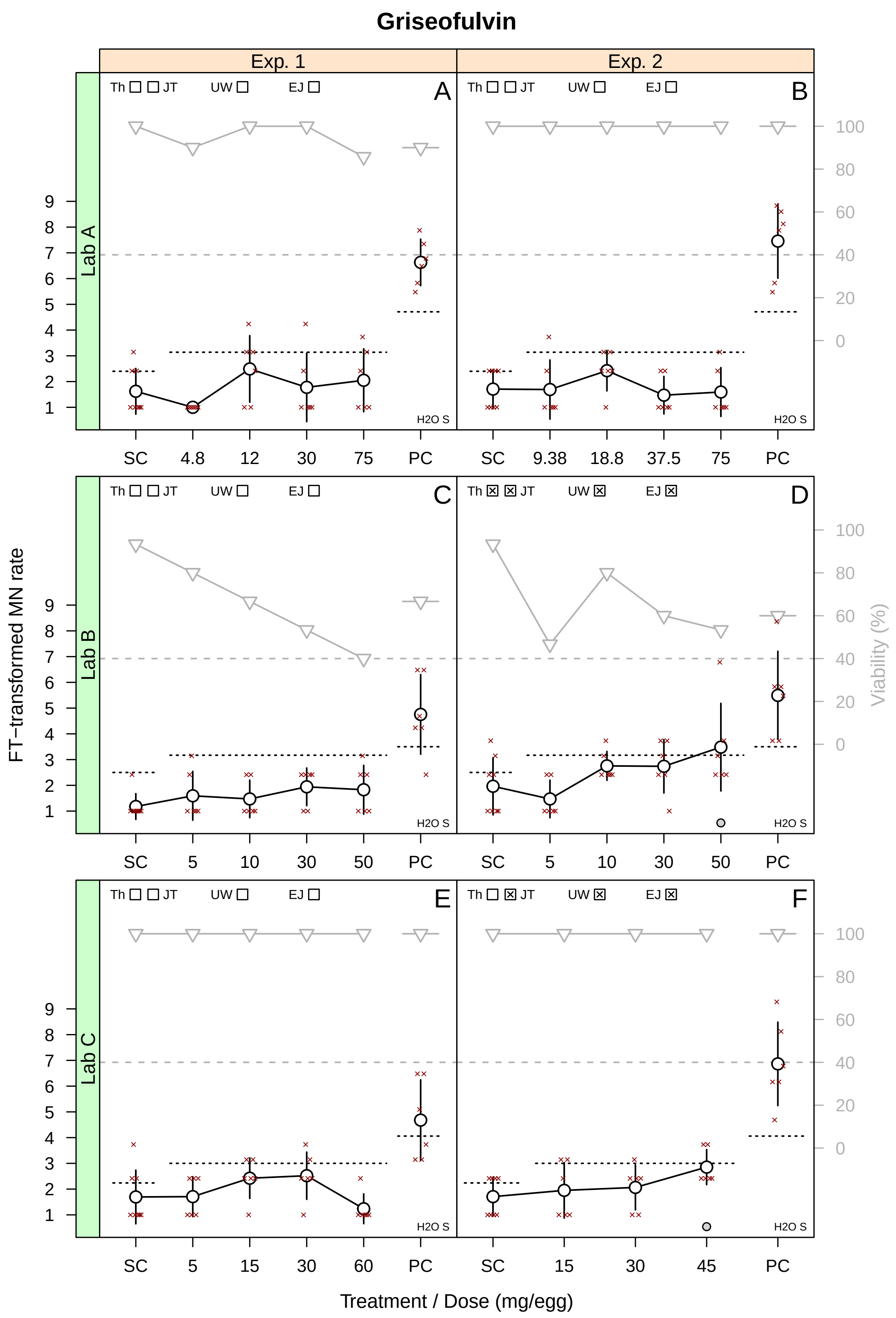
Griseofulvin
Exp. 1
Exp. 2
Lab A
Lab B
Lab C
A
B
C
D
E
F
Th JT UW EJ
H2O S
SC
4.8
12
30
75
PC
9.38
18.8
37.5
5
10
50
15
60
45
FT−transformed MN rate
Viability (%)
Treatment / Dose (mg/egg)

Figure S21

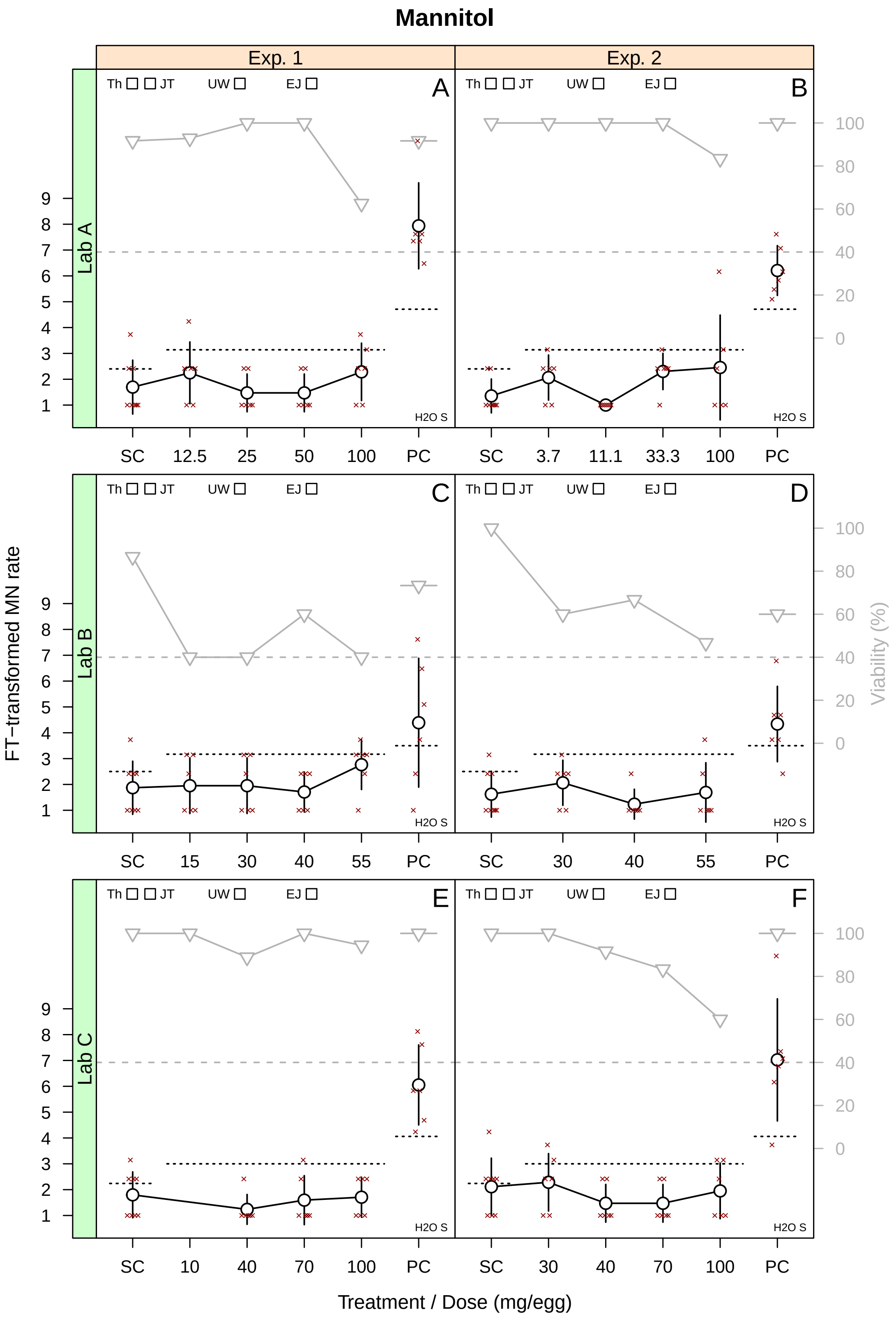
Mannitol
Exp. 1
Exp. 2
Lab A
Lab B
Lab C
A
B
C
D
E
F
Th
JT
UW
EJ
H2O S
SC
12.5
25
50
100
PC
3.7
11.1
33.3
15
30
40
55
10
70
FT−transformed MN rate
Viability (%)
Treatment / Dose (mg/egg)

Figure S22

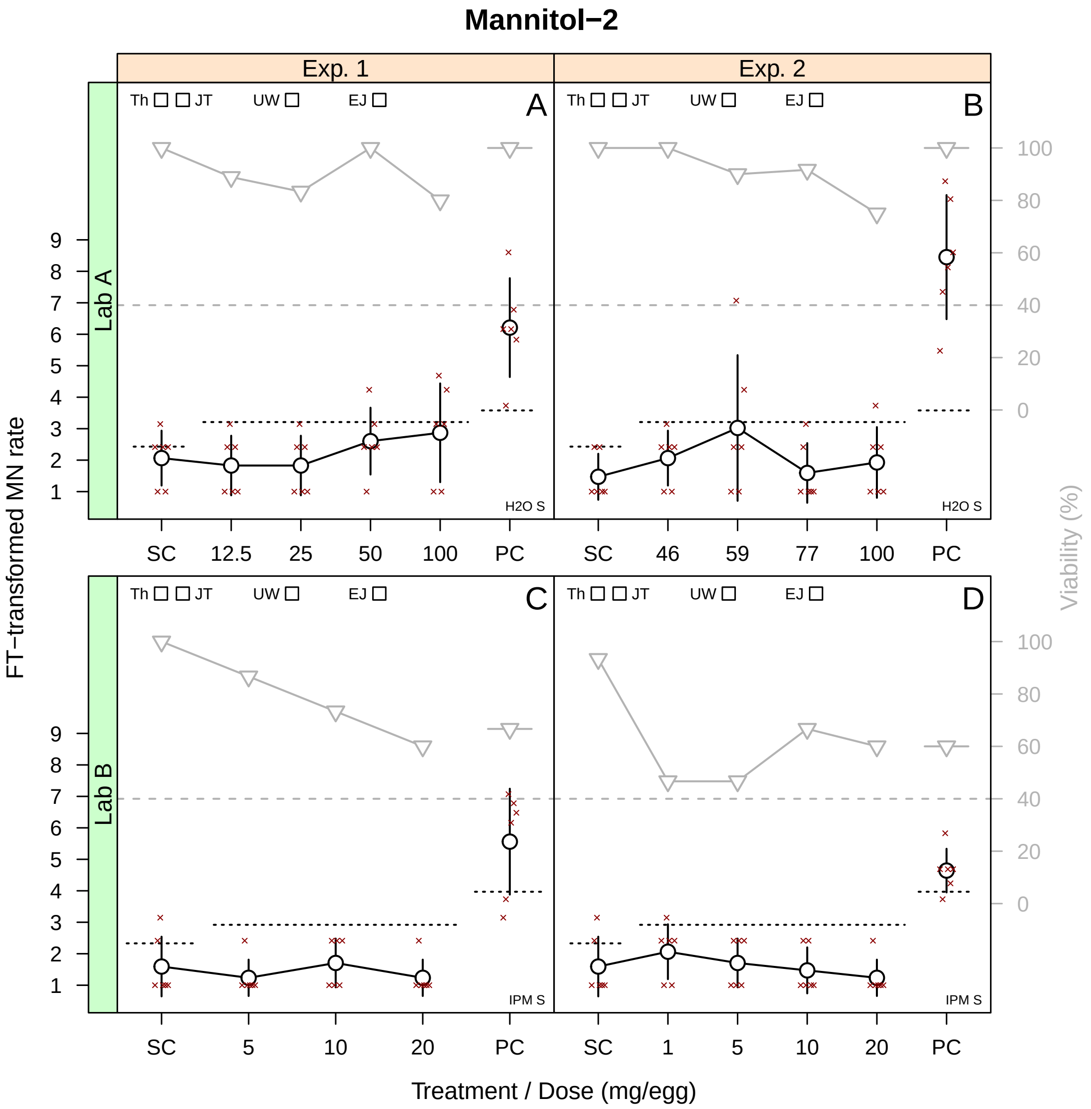

Mannitol−2
Exp. 1
Exp. 2
Lab A
Lab B
Th JT UW EJ
A
B
C
D
H2O S
IPM S
SC
12.5
25
50
100
PC
46
59
77
5
10
20
1
FT−transformed MN rate
Viability (%)
Treatment / Dose (mg/egg)

Figure S23

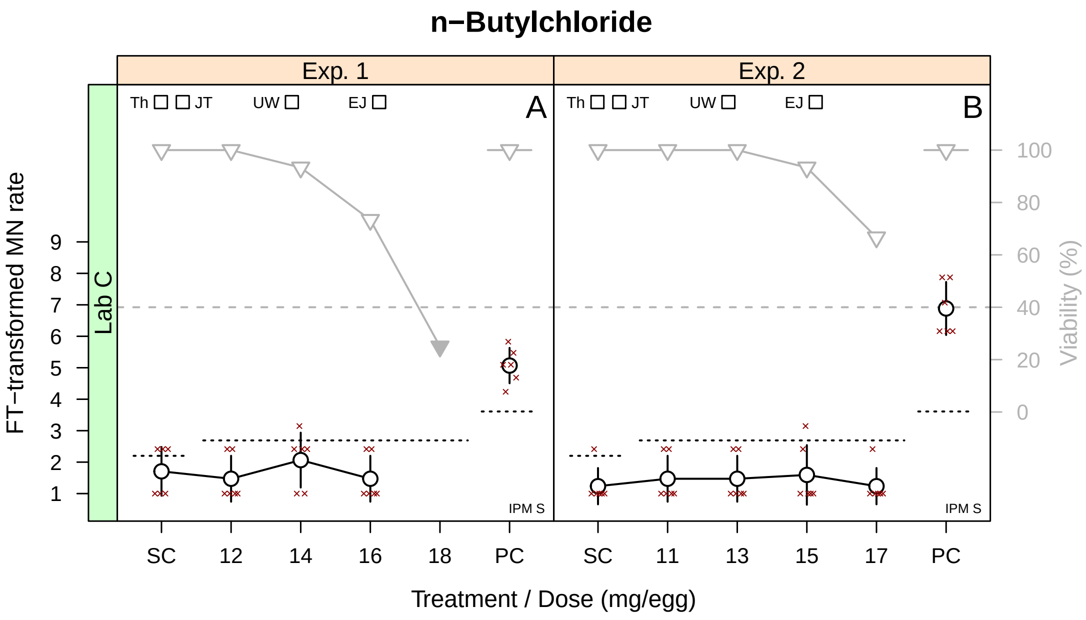
n−Butylchloride
Exp. 1
Exp. 2
Th
JT
UW
EJ
A
B
IPM S
Lab C
FT−transformed MN rate
Viability (%)
1
2
3
4
5
6
7
8
9
0
20
40
60
80
100
SC
12
14
16
18
PC
SC
11
13
15
17
PC
Treatment / Dose (mg/egg)

Figure S24

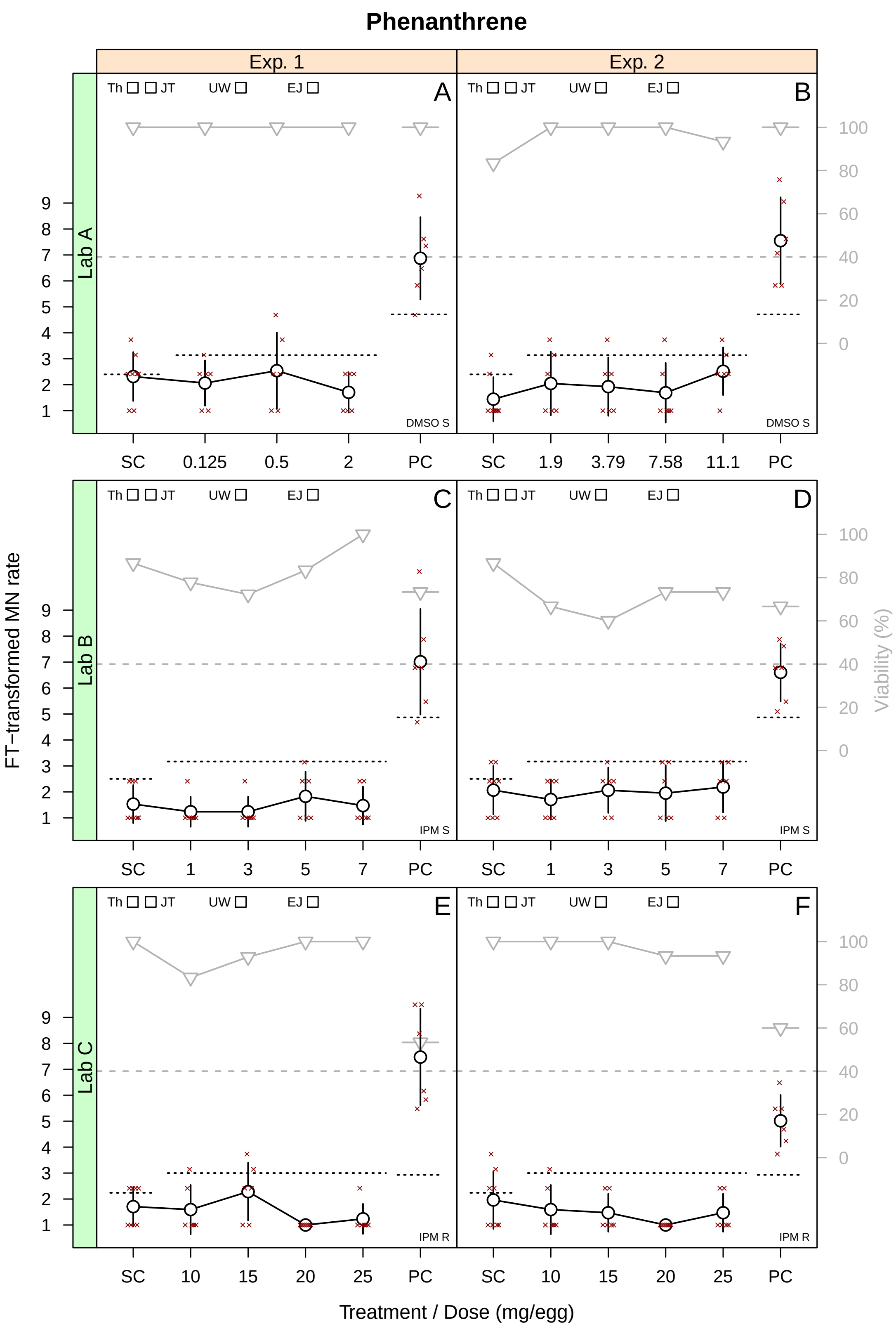

Phenanthrene
Exp. 1
Exp. 2
Lab A
Lab B
Lab C
A
B
C
D
E
F
Th
JT
UW
EJ
DMSO S
IPM S
IPM R
SC
0.125
0.5
2
PC
1.9
3.79
7.58
11.1
1
3
5
7
10
15
20
25
FT−transformed MN rate
Viability (%)
Treatment / Dose (mg/egg)

Figure S25

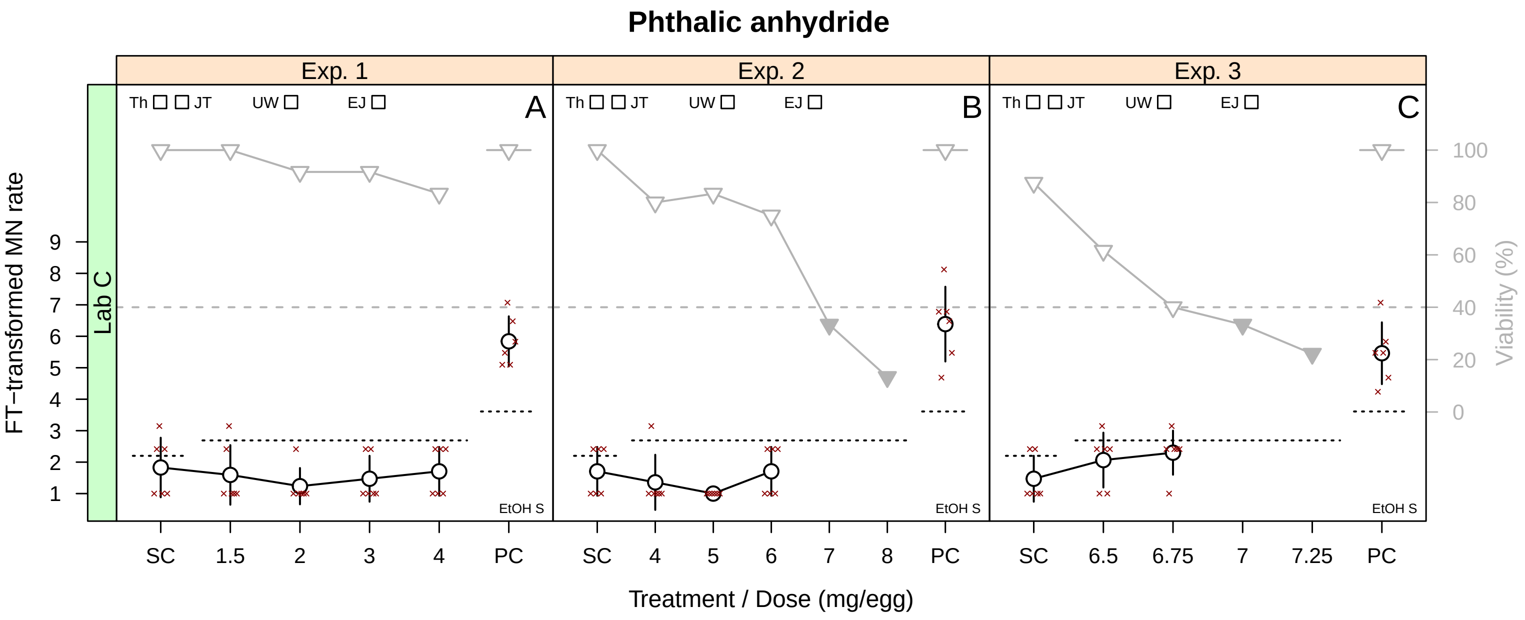

Phthalic anhydride
Exp. 1
Exp. 2
Exp. 3
Lab C
A
B
C
Th JT UW EJ
EtOH S
FT−transformed MN rate
Viability (%)
SC 1.5 2 3 4 PC
SC 4 5 6 7 8 PC
SC 6.5 6.75 7 7.25 PC
Treatment / Dose (mg/egg)

Figure S26

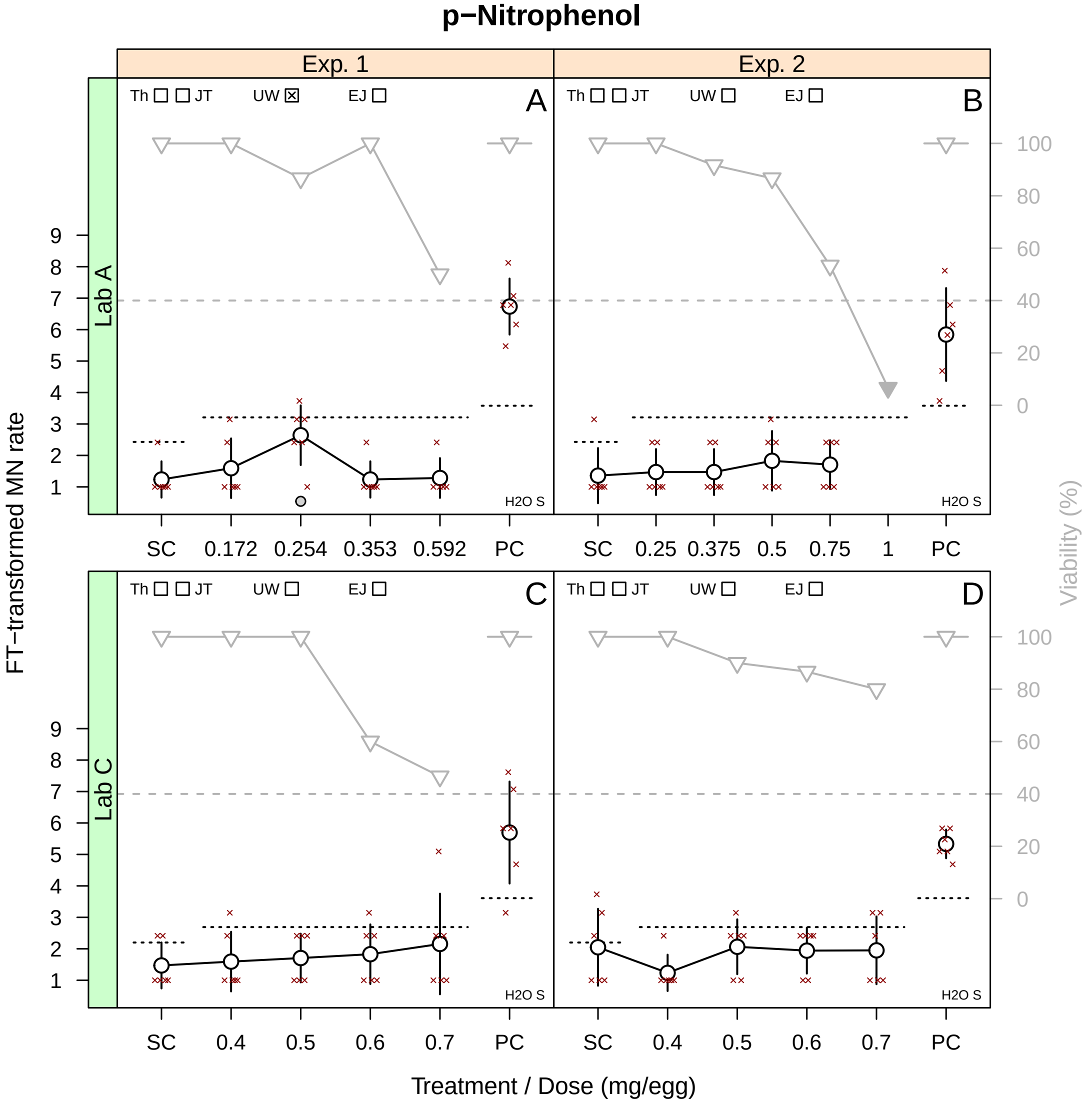

p−Nitrophenol
Exp. 1
Exp. 2
Lab A
Lab C
A
B
C
D
Th JT UW EJ
H2O S
SC 0.172 0.254 0.353 0.592 PC
SC 0.25 0.375 0.5 0.75 1 PC
SC 0.4 0.5 0.6 0.7 PC
SC 0.4 0.5 0.6 0.7 PC
FT−transformed MN rate
Viability (%)
Treatment / Dose (mg/egg)

Figure S27

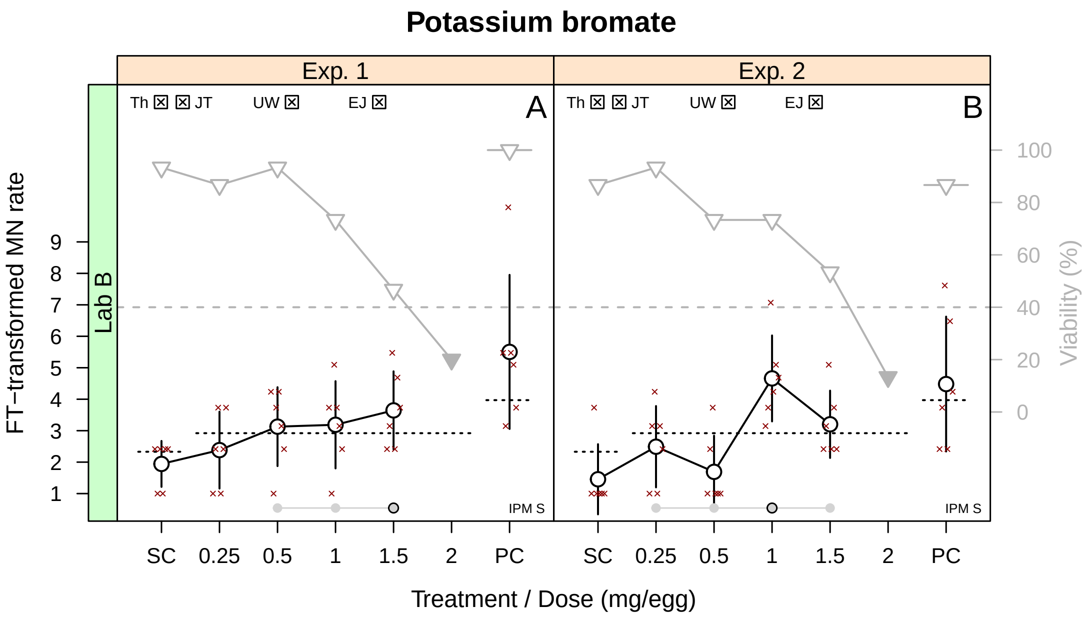
Potassium bromate
Exp. 1
Exp. 2
Lab B
Th ☒ ☒ JT
UW ☒
EJ ☒
A
B
IPM S
FT−transformed MN rate
Viability (%)
9
8
7
6
5
4
3
2
1
100
80
60
40
20
0
SC
0.25
0.5
1
1.5
2
PC
Treatment / Dose (mg/egg)

Figure S28

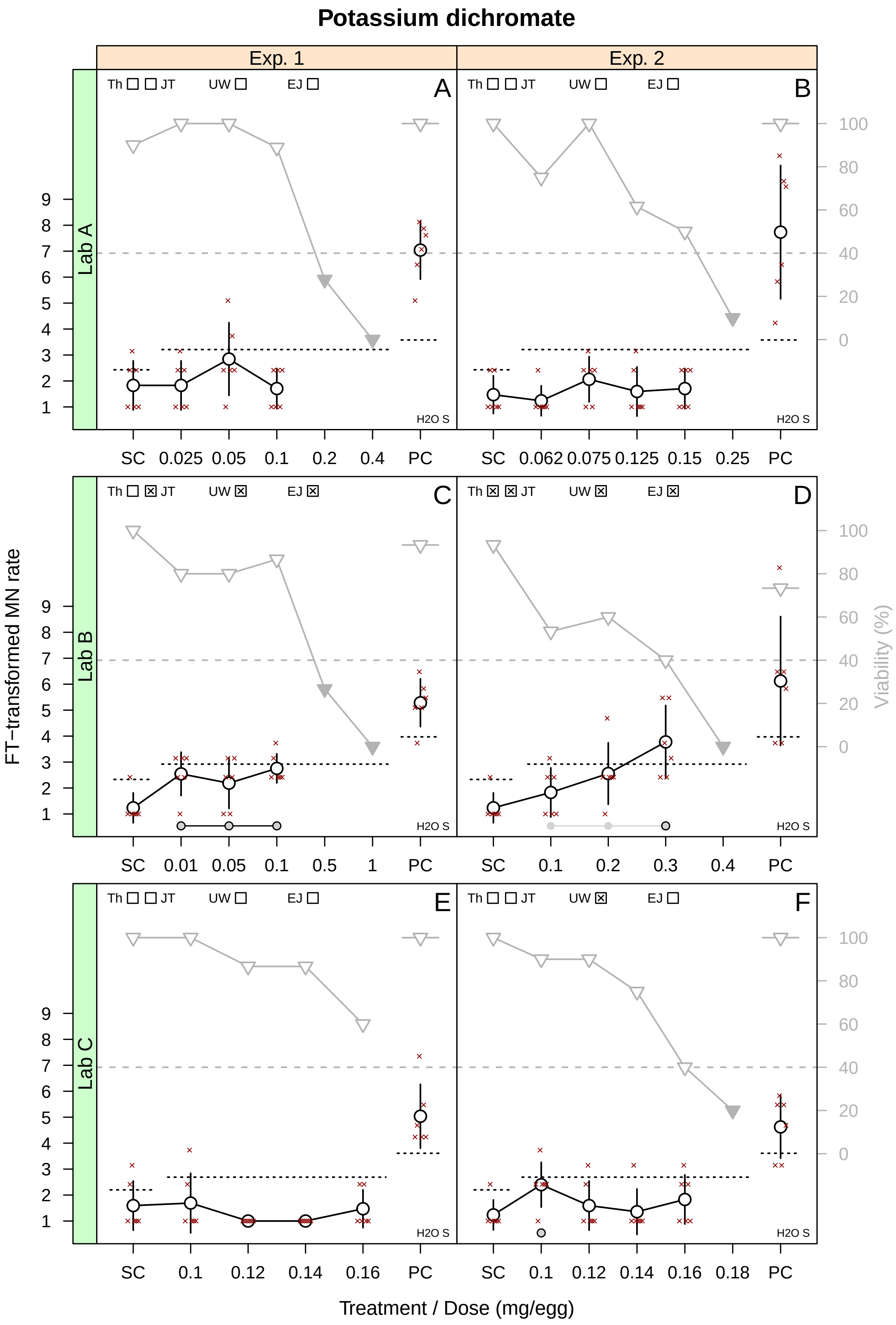
Potassium dichromate
Exp. 1
Exp. 2
Lab A
Lab B
Lab C
A
B
C
D
E
F
Th JT UW EJ
H2O S
FT−transformed MN rate
Viability (%)
SC 0.025 0.05 0.1 0.2 0.4 PC
SC 0.062 0.075 0.125 0.15 0.25 PC
SC 0.01 0.05 0.1 0.5 1 PC
SC 0.1 0.2 0.3 0.4 PC
SC 0.1 0.12 0.14 0.16 PC
SC 0.1 0.12 0.14 0.16 0.18 PC
Treatment / Dose (mg/egg)

Figure S29

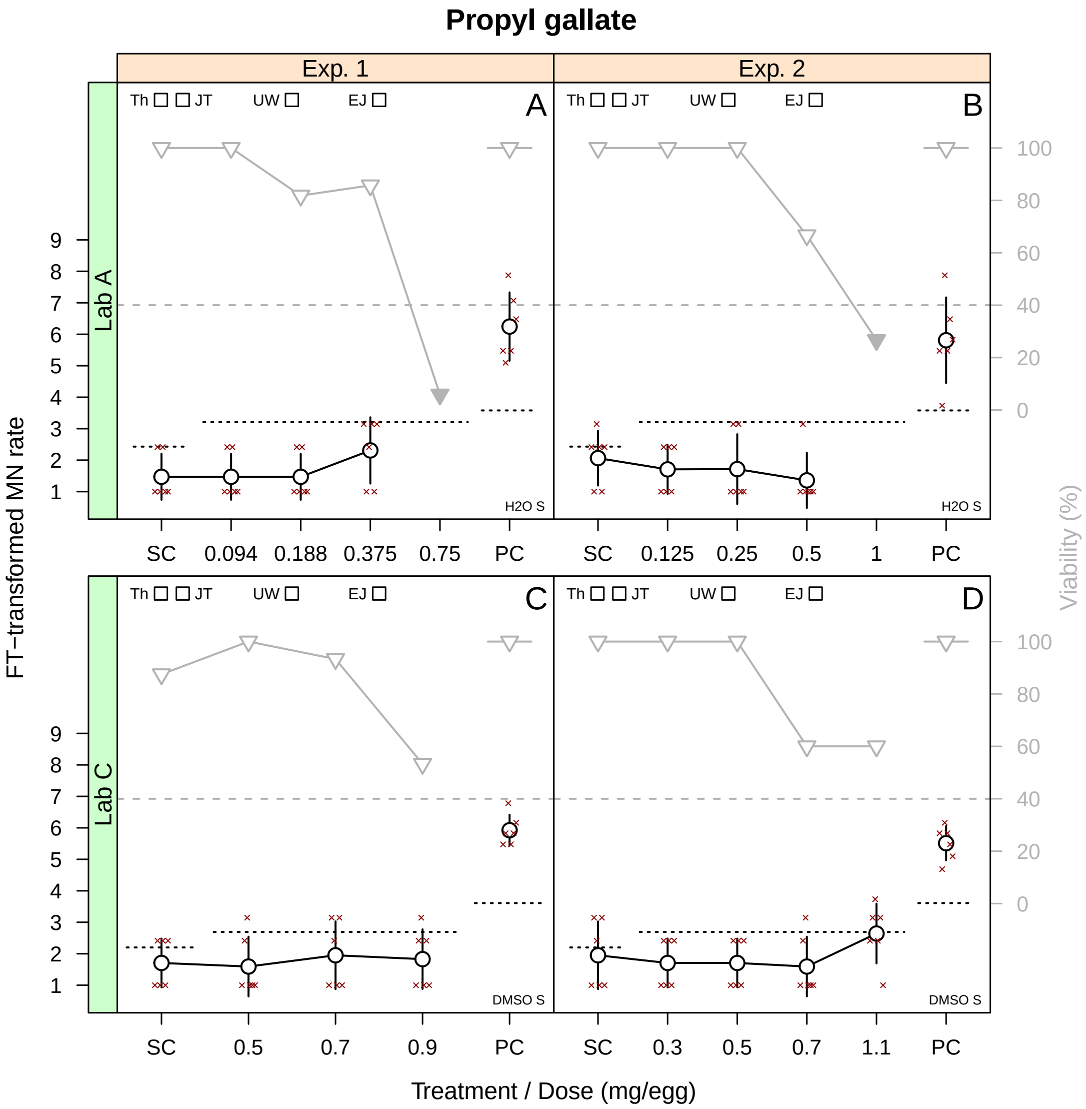

Propyl gallate
Exp. 1
Exp. 2
Lab A
Lab C
A
B
C
D
Th JT UW EJ
H2O S
DMSO S
FT−transformed MN rate
Viability (%)
1 2 3 4 5 6 7 8 9
0 20 40 60 80 100
SC 0.094 0.188 0.375 0.75 PC
SC 0.125 0.25 0.5 1 PC
SC 0.5 0.7 0.9 PC
SC 0.3 0.5 0.7 1.1 PC
Treatment / Dose (mg/egg)

Figure S30

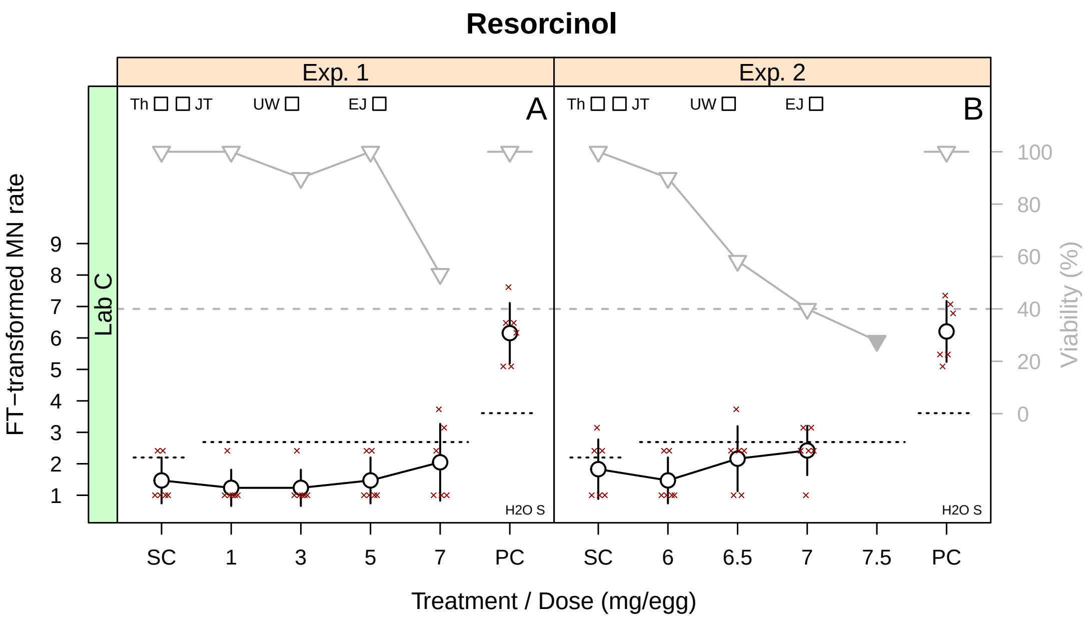
Resorcinol
Exp. 1
Exp. 2
Th JT UW EJ
A
B
Lab C
FT−transformed MN rate
Viability (%)
H2O S
SC 1 3 5 7 PC
SC 6 6.5 7 7.5 PC
Treatment / Dose (mg/egg)

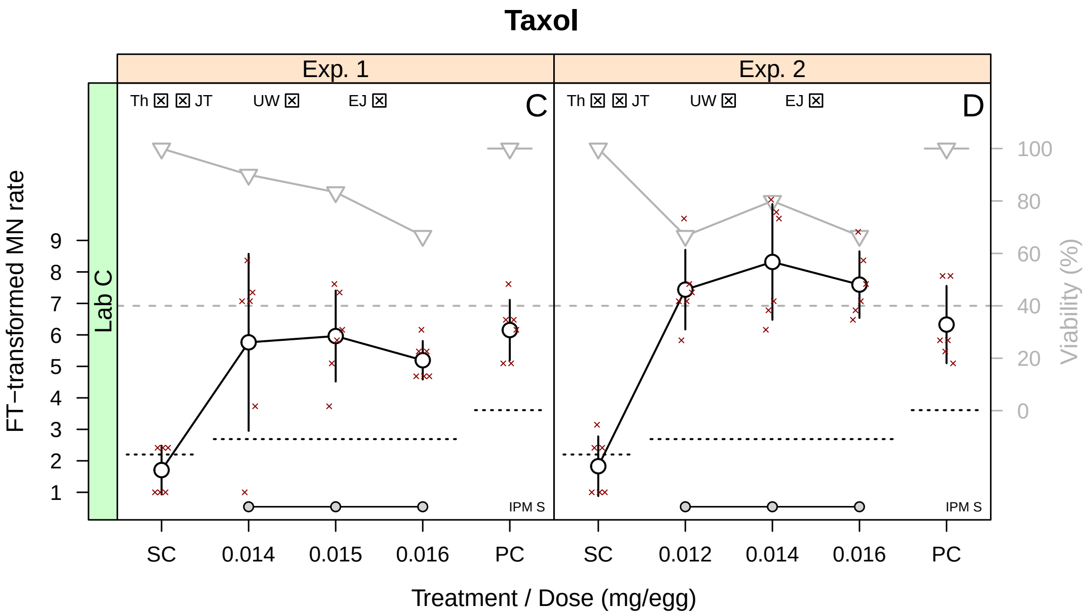
Taxol
Exp. 1
Exp. 2
Th JT UW EJ
C
D
Lab C
FT−transformed MN rate
Viability (%)
IPM S
SC 0.014 0.015 0.016 PC
SC 0.012 0.014 0.016 PC
Treatment / Dose (mg/egg)

Figure S31

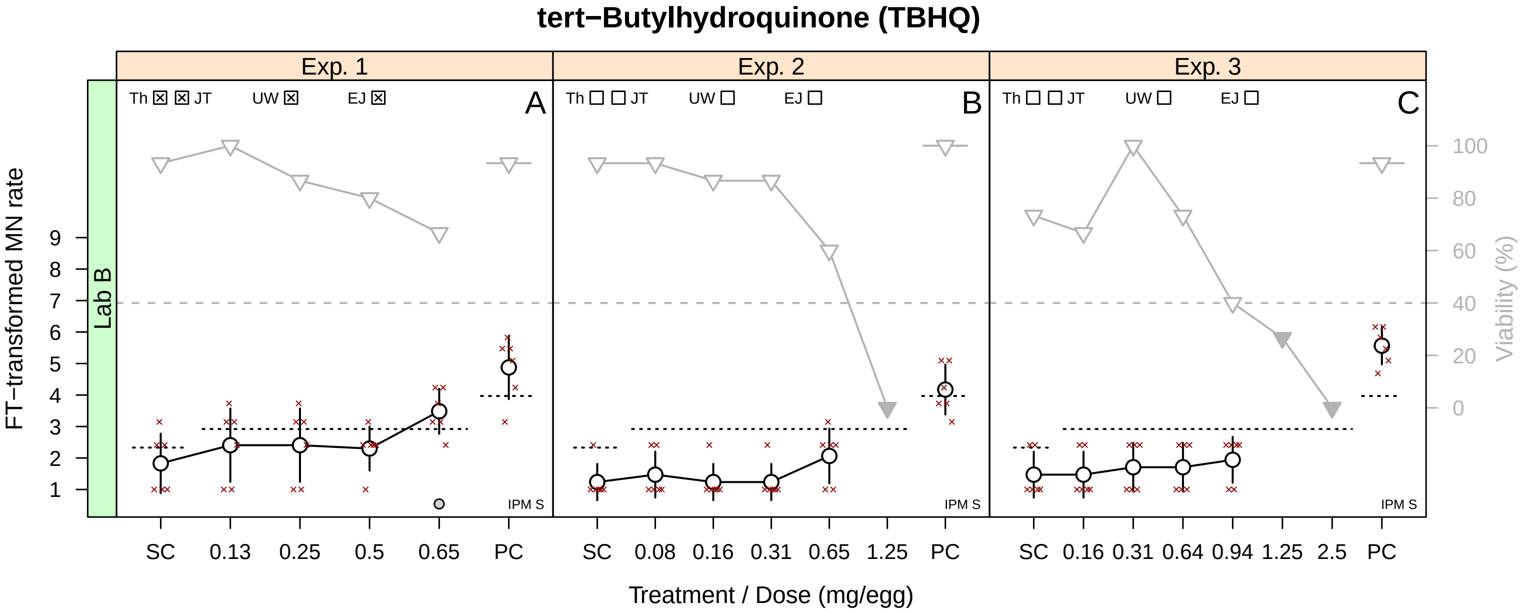

tert−Butylhydroquinone (TBHQ)
Exp. 1
Exp. 2
Exp. 3
Lab B
A
B
C
Th JT UW EJ
IPM S
FT−transformed MN rate
Viability (%)
SC 0.13 0.25 0.5 0.65 PC
SC 0.08 0.16 0.31 0.65 1.25 PC
SC 0.16 0.31 0.64 0.94 1.25 2.5 PC
Treatment / Dose (mg/egg)
