# Supplementary figures and images for: The hen’s egg test for micronucleus induction (HET-MN): validation data set
Source: Mutagenesis. 2021 Jun 3;37(2):61–75. doi: 10.1093/mutage/geab016 (PMC9071061; doi:10.1093/mutage/geab016)

# Figure S32

## Eugenol

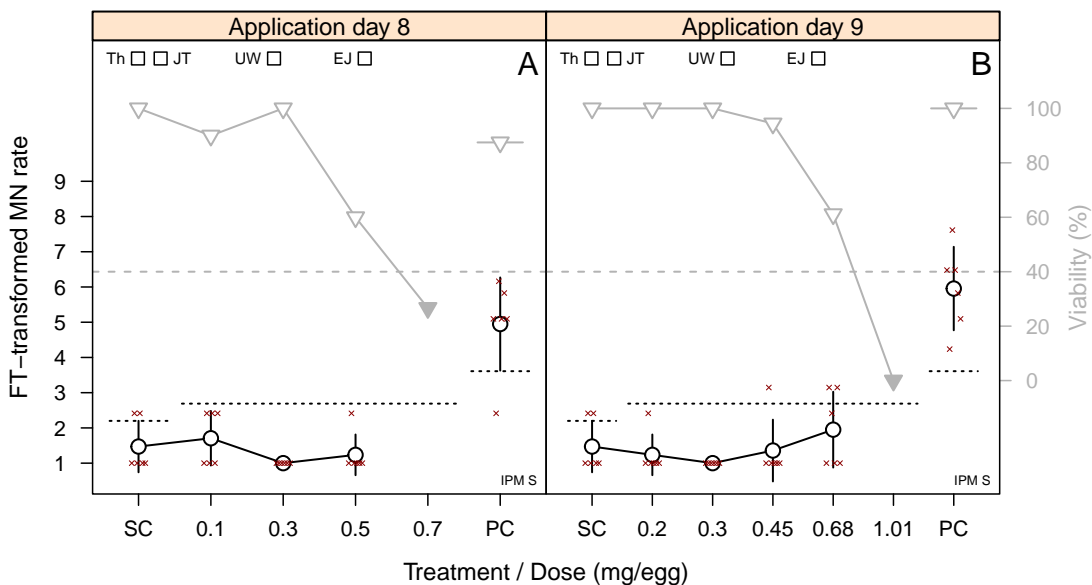

## p-Nitrophenol

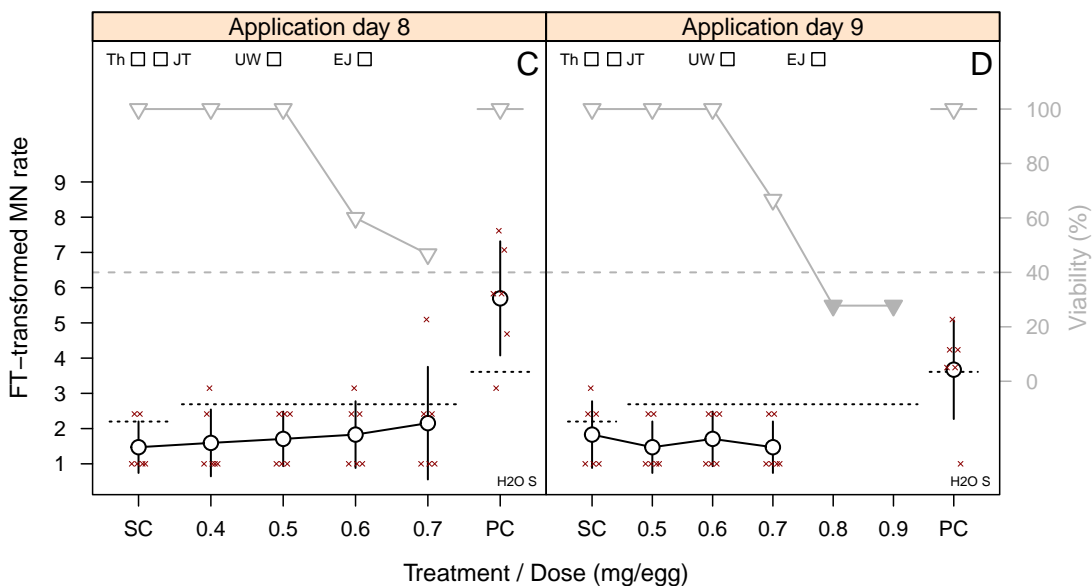

# Figure S33

## Potassium dichromate

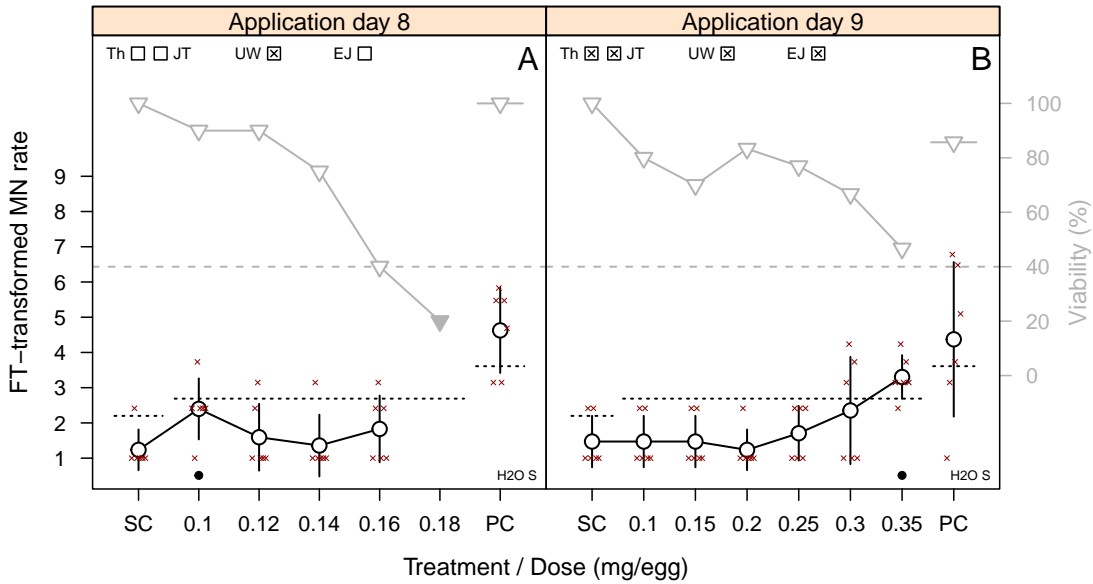

## Resorcinol

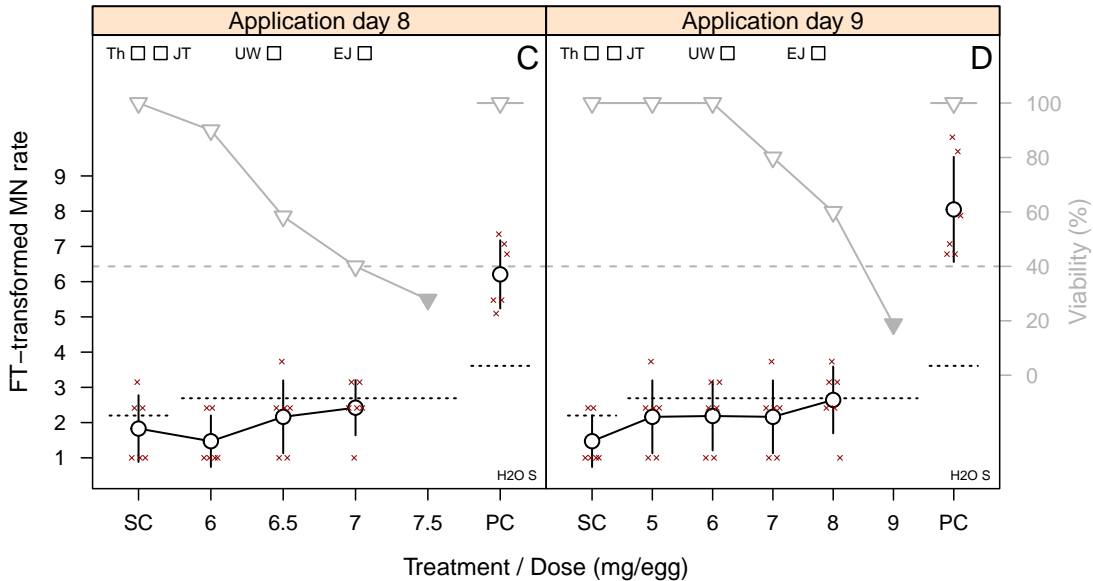

Supplement: geab016_suppl_Supplementary_Figure_S32_S33 [file geab016_suppl_supplementary_figure_s32_s33.pdf]

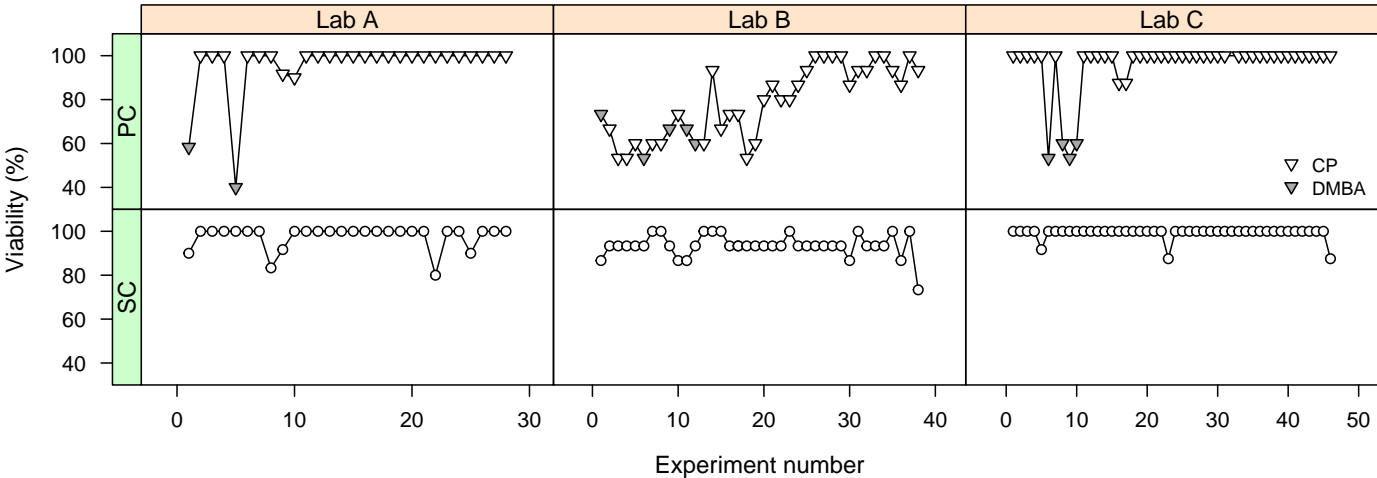

Supplement: geab016_suppl_Supplementary_Figure_S34 [file geab016_suppl_supplementary_figure_s34.pdf]
