# Supplementary material for: The hen’s egg test for micronucleus induction (HET-MN): validation data set
Source: Mutagenesis. 2021 Jun 3;37(2):61–75. doi: 10.1093/mutage/geab016 (PMC9071061; doi:10.1093/mutage/geab016)
Supplement: geab016_suppl_Supplementary_Table_S1 [file geab016_suppl_supplementary_table_s1.docx]

**Supplemental Table S1. Literature information on the *in vitro* and *in vivo* genotoxicity of the 32 chemicals included in the final evaluation.** Test results were compiled for the bacterial reverse mutation test (Ames test), micronucleus (MN) test, sister chromatid exchange (SCE) assay, unscheduled DNA synthesis (UDS) test, mouse lymphoma assay (MLA), chromosomal aberration (CA) test, mammalian cell HPRT gene mutation assay, and transgenic rodent (TGR) gene mutation assay. TN = true negative; MP = misleading positive; TP = true positive; NT = not tested; Pos = positive in test; Neg = negative in test; Inc = inconclusive in test; Eqv = equivocal in test; +S9 = in the presence of S9; –S9 = in the absence of S9; ±S9 = in the absence or presence of S9; Cat. = category; BM = bone marrow; CHL or V79 = Chinese hamster lung fibroblast cells; CHO = Chinese hamster ovary cells; CHL-D = Chinese hamster lung (Don) cells; HepG2 = human liver cancer cell line; Hu-Fib = human fibroblasts; HuLy = human lymphocytes; PRH = primary rat hepatocytes; PHH = primary human hepatocytes; TK6 = human lymphoblast cells; UD = unpublished data; i.p.= intraperitoneal; p.o. = per os.

| **Fig. No.** | **Chemical (CAS No)** | **Cat.** | ***In vitro*** | | | ***In vivo*** | | |
| --- | --- | --- | --- | --- | --- | --- | --- | --- |
|  | **Expected positive results** |  | **Assay** | **Result** | **Ref.** | **Assay** | **Result** | **Ref.** |
| S1 | 2-Aminoanthracene  (613-13-8) | TP | **Ames**  **MN**  **SCE**  **CA** | Pos (+S9)  Pos (±S9)  Pos (CHO, CHL-D ±S9)  Eqv (CHO +S9) | (30,31)  (1,32)  (33,34)  (1,34) | **No data** |  |  |
| S2 | 2-Acetylaminofluorene  (2-AAF)  (53-96-3) | TP | **Ames**  **MN**  **SCE**  **UDS**  **MLA**  **CA**  **HPRT** | Pos (+S9 (–S9 NT))  Pos (±S9)  Pos (human skin fibroblasts –S9, CHO+S9)  Pos (PRH)  Pos (±S9)  Pos (CHL ±S9), Neg (V79 –S9)  Eqv (variable results) | (1-4)  (1)  (5)  (6)  (7,8)  (9,10)  (11-13) | **MN**  **UDS**  **Comet**  **CA**  **TGR** | Pos (BM)  Pos (liver)  Pos (blood) and one Neg  Pos (blood)  Pos (liver) | (10,14,15)  (16-18)  (19-25)  (14,26)  (27-29) |
| S3 | (2-Chloroethyl)trimethyl-ammonium chloride  (999-81-5) | TN | **Ames**  **SCE**  **CA**  **HPRT** | Neg (+S9)  Eqv (variable results)  Neg (no info on S9) (but re-evaluated as “uninterpretable”)  Neg (no info on S9) | (35-39)  (36)  (36,40)  (36) | **CA** | Neg (BM) | (39) |
| S3 | 2-Ethyl-1,3-hexandiol  (94-96-2) | MP | **Ames**  **MN**  **SCE**  **MLA**  **CA**  **HPRT** | Neg (±S9)  Neg (CHL, TK6 ±S9)  Neg (±S9)  Neg  Pos (+S9 only + high concs)  Neg (±S9) | (41-43)  (44)  (41)  (45)  (41,42)  (41,42) | **MN**  **CA** | Neg (blood)  Neg (BM) | (41)  (41) |
| S4 | 2,4-Diaminotoluene  (2,4-DAT)  (95-80-7) | TP | **Ames**  **MN**  **SCE**  **UDS**  **Comet**  **MLA**  **CA**  **HPRT** | Pos (+S9)  Pos (HepG2 –S9)  Pos (±S9)  Pos (HepG2 –S9)  Pos (HepG2 –S9)  Pos (–S9 only)  Pos (±S9)  Neg (±S9) | (30,38,46-50)  (51)  (52,53)  (51,54)  (51)  (55,56)  (52,53,57)  (11) | **MN**  **UDS**  **Comet**  **TGR** | Weak Pos/Neg (BM), Pos (liver)  Pos (liver)  Pos (liver but not stomach)  Pos (liver, kidney but not skin) | (15,37,58)  (16,17,58)  (25,59,60)  (29,61,62) |
| S5 | 2,4-Dichlorophenol  (120-83-2) | MP | **Ames**  **MN**  **SCE**  **UDS**  **MLA**  **CA**  **HPRT** | Neg (±S9)  Pos (V79, CHO, CHL ±S9); Neg (HuLy, TK6, HepG2 ±S9)  Pos (±S9)  Neg  Pos (-S9)  Neg (CHO ±S9); Eqv (CHO ±S9 at high doses)  Neg (V79 -S9 [+S9 NT]) | (38,63)  (44)  (64)  (65)  (66)  (64,67)  (68) | **CA**  **Comet** | Pos (BM, spermatocytes)  Pos (stomach, colon) | (69)  (70) |
| S6 | 4-Nitroquinoline 1-oxide  (56-57-5) | TP | **Ames**  **MN**  **SCE**  **UDS**  **MLA**  **CA**  **HPRT** | Pos (±S9)  Pos  Pos (HuLy -S9)  Pos (HepG2)  Pos (±S9)  Pos (several cell types -S9)  Pos (-S9) | (1,2,30,71-74)  (75-77)  (78,79)  (6,80,81)  (8,56,76,82)  (1,9,79,83)  (12,84-86) | **MN**  **Comet**  **CA**  **TGR** | Pos (BM)  Mainly Pos (multiple tissues inc. skin)  Pos (BM)  Pos (several organs mouse (i.p.), oral cavity (p.o.)) | (87-89)  (70,90-96)  (97,98)  (87,99-101) |
| S6 | 4-Vinyl-1-cyclohexene diepoxide  (106-87-6) | TP | **Ames**  **MN**  **SCE**  **MLA**  **CA**  **HPRT** | Neg (+S9)  Neg (V79 lung -S9, +S9 NT)  Pos (±S9)  Pos (-S9 [+S9 NT])  Pos (±S9)  Neg (-S9, [+S9 NT]) | (102)  (103)  (102,103)  (102,104)  (102)  (103,105) | **No data** |  |  |
| S7 | 5-Fluorouracil  (51-21-8) | TP | **Ames**  **MN**  **MLA**  **CA** | Neg (±S9)  Pos (L5178Y ML cells); Neg (V79 -S9)  Pos (±S9)  Pos (CHO, CHL - S9) | (106-108)  (109-113)  (114)  (1,9) | **MN**  **Comet** | Pos (blood)  Neg (liver, stomach) | (15,115-118)  (25,119) |
| S8 | 8-Hydroxyquinoline  (148-24-3) | TP i.p. | **Ames**  **SCE**  **MLA**  **CA** | Pos (+S9)  Pos (CHO ±S9)  Pos (-S9)  Weakly pos (CHO +S9); Neg (CHO - S9) | (47)  (52,120)  (104)  (52) | **MN**  **Lifetime studies** | Pos (BM)  Pos (tumor at application site) | (121)  (122) |
|  |  | TN  p.o. |  |  |  | **MN**  **UDS**  **Comet**  **CA** | Neg (BM)  Neg (liver)  Neg (stomach, liver)  Neg (BM) | (120)  (123)  (25)  (97,120) |
| S9 | Aniline  (62-53-3) | TP | **Ames**  **SCE**  **MLA**  **CA** | Neg (±S9)  Pos (±S9)  Pos (±S9)  Pos (weak in CHO +S9) | (1,63)  (124)  (125)  (1,124) | **MN** | Pos (blood, BM) | (126,127) |
| S10 | Benzo[*a*]pyrene  (BaP)  (50-32-8) | TP | **Ames**  **MN**  **SCE**  **MLA**  **CA**  **HPRT** | Pos (+S9)  Pos (TK6 +S9)  Pos (no info on S9)  Pos (+S9) Neg (–S9)  Pos (CHL +S9)  Pos (V79, CHO+S9); Neg (–S9) | (1,63,128)  (129)  (130)  (8,131)  (1,130)  (84,132) | **MN**  **UDS**  **Comet**  **CA**  **TGR** | Pos (blood)  Pos (liver)  Pos (various tissues)  Pos (BM)  Pos (various tissues) | (133)  (16,134)  (19,20,22,135-143)  (130)  (144-146) |
| S11 | Cadmium sulfate  (10124-36-4) | TP | **Ames**  **MN**  **SCE**  **UDS**  **MLA**  **CA** | Mostly Neg in std strains (±S9), weakly Pos TA102 ±S9)  Pos (-S9)  Neg (HuLy -S9, +S9 NT)  Pos (PRH)  Pos (-S9)  Pos (CHO -S9, +S9 NT, Huly) | (147)  (1)  (147)  (147)  (147)  (1,147) | **No data** |  |  |
| S12 | Curcumin  (458-37-7) | MP | **Ames**  **MN** | Neg (±S9)  Pos (V79, CHO, CHL, HuLy, TK6 ±S9 apoptosis); Neg (HepG2 ±S9) | (35)  (1,148) | **MN** | Neg (BM) | (149) |
| S13 | Cyclohexanone  (108-94-1) | TN | **Ames**  **MN**  **MLA**  **CA**  **HPRT** | Neg (±S9)  Inc - only tested -S9  Neg (±S9)  Pos (aneuploidy); Neg (HuLy -S9)  Neg (CHO ±S9 | (63)  (150)  (104)  (150-152)  (153) | **MN**  **CA** | Experiments unreliable  Eqv: Neg (BM), Pos (blood)  EFSA & IARC final conclusion: Neg | (153)  (153,154)  (155,156) |
| S14 | Diclofenac  (15307-79-6) | TN | **Ames**  **MLA**  **CA**  **HPRT** | Neg (±S9)  Neg  Neg (HuLy ±S9, Rat-Ly +S9)  Neg (±S9) | (30,157-161)  (162)  (158,163)  (162) | **MN** | Neg (BM) | (158,162,163) |
| S15/S30 | Resorcinol  (1,3-Dihydroxybenzene)  (108-46-3) | MP | **Ames**  **MN**  **SCE**  **MLA**  **CA** | Neg (±S9)  Pos (V79, CHO, CHL, HepG2), Neg HuLy, TK6 ±S9)  Pos (V79 -S9, [+S9 NT], HuLy)  Pos (-S9, [+S9 NT])  Pos (CHO, CHL, HuLy ±S9) | (63,234,235)  (44)  (236,237)  (104)  (238-241) | **MN**  **SCE** | Neg (BM)  Neg (BM) | (239,242)  (243) |
| S16 | Ethionamide  (536-33-4) | MP | **Ames**  **MN**  **SCE**  **CA** | Neg (±S9)  Neg (V79, CHO, CHL, HuLy, TK6, HepG2 ±S9)  Neg (no info on S9)  Weak Pos at high concs. with precipitation | (47,164)  (44)  (165)  (9,67) | **Comet** | Neg (liver, stomach) | (25,166) |
| S17 | Ethyl methanesulfonate  (EMS)  (62-50-0) | TP | **Ames**  **MN**  **SCE**  **UDS**  **MLA**  **CA**  **HPRT** | Pos (±S9)  Pos (CHL -S9)  Pos (HuLy, V79, CHO -S9)  Pos (Hu-Fib)  Pos (-S9)  Pos (several cell types -S9)  Pos (-S9) | (35)  (167)  (168,169)  (6)  (7,170,171)  (169)  (84,171-173) | **MN**  **Comet**  **CA**  **TGR** | Pos (BM)  Pos (multiple tissues)  Pos (BM)  Pos (BM) | (174)  (19,22,70,175-179)  (180)  (174) |
| S18 | Etoposide  (33419-42-0) | TP | **Ames**  **MN**  **SCE**  **Comet**  **MLA**  **CA**  **HPRT** | Pos (±S9)  Pos (-S9, [+S9 NT])  Pos (-S9)  Pos (CHO -S9)  Pos (-S9)  Pos (-S9)  Pos (-S9) | (181-184)  (111,185-187)  (181)  (181,186,188)  (181,184,186)  (181)  (181) | **MN**  **Comet**  **CA** | Pos (BM)  Pos (multiple tissues)  Pos (BM) | (181,182)  (188)  (181) |
| S19 | Eugenol  (97-53-0) | MP | **Ames**  **MN**  **MLA**  **CA** | Neg (±S9)  Neg (HuLy, TK6±S9); Pos (V79, CHO, CHL, HepG2 ±S9)  Pos (±S9)  Pos (V79 ±S9) | (38,63,189)  (44)  (8,56,114)  (124,190-192) | **MN**  **UDS**  **TGR** | Pos at high doses by i.p. in mice but mostly Neg results by i.p. and p.o. (BM)  Neg (liver)  Neg (liver) | (193-198)  (197,199)  (29,199) |
| S20 | Griseofulvin  (126-07-8) | TP | **Ames**  **MN**  **UDS**  **CA** | Neg (±S9)  Pos (HuLy, rodent cells e.g. V79 -S9, PRH, +S9, [-S9 NT])  Neg (PRH)  Pos (HuLy -S9) | (200)  (1,200)  (200)  (1) | **MN**  **CA**  **SCE** | Neg (BM), (Pos)  Neg (BM); Pos (spermatocytes)  Pos (BM) | (200)  (200)  (200) |
| S21/S22 | D-Mannitol  (69-65-8) | TN | **Ames**  **SCE**  **MLA**  **CA** | Neg (±S9)  Neg (CHO ±S9)  Neg (±S9, but “uninterpretable”)  Neg (CHO ±S9) | (38,63,106,201)  (202)  (40,56,203)  (202,204) | **MN**  **CA** | Neg (BM)  Neg (BM) | (195)  (204,205) |
| S23 | n-Butyl chloride  (109-69-3) | TN | **Ames**  **MN**  **SCE**  **MLA**  **CA** | Neg (±S9)  Neg (-S9)  Neg (CHO ±S9)  Neg (±S9 but re-evaluated as “uninterpretable”)  Neg (CHO ±S9) | (107,206,207)  (208)  (64)  (40,56,66)  (64) | **No data** |  |  |
| S24 | Phenanthrene  (85-01-8) | TN | **Ames**  **MN**  **MLA**  **CA**  **HPRT** | Neg in most reports (except 1)  Neg (h1A1v2 -S9, MCL-5 cells)  Neg (h1A1v2 -S9, MCL-5 cells)  Neg (CHL, HuLy -S9)  Neg (MCL-5) but only single conc. tested | (30,47,128,209-211)  (212,213)  (212,214)  (83)  (212) | **MN** | Neg (blood) | (215) |
| S25 | Phthalic anhydride  (85-44-9) | MP | **Ames**  **MN**  **SCE**  **MLA**  **CA** | Neg (±S9)  Neg (V79, CHO, HuLy, HepG2 ±S9), Pos (CHL, TK6 ±S9)  Neg (CHO ±S9)  Pos (no info on S9)  Neg (CHO ±S9) | (216)  (44)  (124)  (217)  (124) | **No data** |  |  |
| S26 | p-Nitrophenol  (100-02-7) | MP | **Ames**  **MN**  **SCE**  **MLA**  **CA**  **HPRT** | Neg (±S9); weak Pos in TA1535  Neg (V79, HepG2, TK6 ±S9); Pos (CHO, CHL, HepG2 ±S9)  Neg (no info on S9)  Neg and Inc results  Pos (-S9 only)  Neg | (63,218-221)  (44)  (222)  (56,218)  (218,223)  (12) | **MN** | Neg (BM) | (224) |
| S27 | Potassium bromate  (7758-01-2) | TP | **Ames**  **MN**  **Comet**  **CA**  **HPRT** | Pos (+S9)  Pos (CHL, V79 -S9)  Pos (V79 -S9)  Pos (±S9)  Pos (V79 -S9) | (225)  (1,226)  (226)  (225)  (226) | **MN**  **CA** | Pos (blood, BM)  Pos (BM) | (225,227)  (225) |
| S28 | Potassium dichromate  (7778-50-9) | TP | **Ames**  **SCE**  **UDS**  **Comet**  **CA**  **HPRT** | Pos (-S9)  Pos (HuLy, CHO -S9)  Neg (Hu-fib -S9)  Neg (V79 -S9)  Pos (CHL ±S9)  Pos (CHO -S9) | (228)  (228-230)  (228)  (228)  (83)  (228) | **MN**  **Comet** | Pos (BM)  Pos (liver, kidneys and lung) | (231)  (231) |
| S29 | Propyl Gallate  (121-79-9) | MP | **Ames**  **MN**  **MLA**  **CA** | Neg (±S9)  Pos (V79, CHO, CHL, TK6 ±S9); Neg (HuLy, HepG2 ±S9)  Pos (-S9 [+S9 NT])  Pos (rodent cell lines ±S9) | (106,201)  (44)  (104)  (232) | **MN**  **CA** | Pos (BM), Neg (BM)  Pos (BM), Neg (BM)  EFSA & IARC final conclusion: Neg | (205,232)  (205,232)  (232,233) |
| S30 | Taxol  (33069-62-4) | TP | **Ames**  **MN**  **MLA**  **CA** | Neg  Pos  Pos (-S9)  Pos (polyploidy) (HuLy -S9) | (164,244)  (75,245-248)  (249)  (250) | **MN** | Pos (BM, blood, duodenum, colon) | (251-253) |
| S31 | Tertiary-butylhydroquinone  (1948-33-0) | MP | **Ames**  **MN**  **CA** | Neg (±S9)  Neg (HuLy, HepG2 ±S9); Pos (V79, CHO, CHL, TK6 ±S9)  Pos (possibly at cytotoxic doses) (no info on S9) | (254-256)  (44)  (257) | **MN**  **Comet**  **CA** | Neg (BM)  Pos ([borderline] liver)  Neg & Pos (BM) | (258)  (25,259)  (258,260) |

References

1. Corvi, R., Albertini, S., Hartung, T., Hoffmann, S., Maurici, D., Pfuhler, S., van Benthem, J., and Vanparys, P. (2008) ECVAM retrospective validation of in vitro micronucleus test (MNT). *Mutagenesis*, **23**, 271-283.

2. Dunkel, V.C., Zeiger, E., Brusick, D., McCoy, E., McGregor, D., Mortelmans, K., Rosenkranz, H.S., and Simmon, V.F. (1985) Reproducibility of microbial mutagenicity assays: II. Testing of carcinogens and noncarcinogens in Salmonella typhimurium and Escherichia coli. *Environ Mutagen*, **7 Suppl 5**, 1-248.

3. Durston, W.E., and Ames, B.N. (1974) A simple method for the detection of mutagens in urine: studies with the carcinogen 2-acetylaminofluorene. *Proc Natl Acad Sci U S A*, **71**, 737-741.

4. PubChem National Center for Biotechnology Information. 2-Acetylaminofluorene, CID=5897, PubChem Online Database <https://pubchem.ncbi.nlm.nih.gov/compound/2-Acetylaminofluorene> (accessed on Jan. 13, 2020).

5. Schneider, E.L., and Gilman, B. (1979) Sister chromatid exchanges and aging. III. The effect of donor age on mutagen-induced sister chromatid exchange in human diploid fibroblasts. *Hum Genet*, **46**, 57-63.

6. Mitchell, A.D., Casciano, D.A., Meltz, M.L., Robinson, D.E., San, R.H., Williams, G.M., and Von Halle, E.S. (1983) Unscheduled DNA synthesis tests. A report of the U.S. Environmental Protection Agency Gene-Tox Program. *Mutation research*, **123**, 363-410.

7. Clive, D., Johnson, K.O., Spector, J.F., Batson, A.G., and Brown, M.M. (1979) Validation and characterization of the L5178Y/TK+/- mouse lymphoma mutagen assay system. *Mutation research*, **59**, 61-108.

8. Myhr, B.C., and Caspary, W.J. (1988) Evaluation of the L5178Y mouse lymphoma cell mutagenesis assay: intralaboratory results for sixty-three coded chemicals tested at Litton Bionetics, Inc. *Environmental and molecular mutagenesis*, **12 Suppl 13**, 103-194.

9. Ishidate, M., Jr., Harnois, M.C., and Sofuni, T. (1988) A comparative analysis of data on the clastogenicity of 951 chemical substances tested in mammalian cell cultures. *Mutation research*, **195**, 151-213.

10. NTP Testing Status of 2-Acetylaminofluorene 10361-C. National Toxicology Program [web site], <https://ntp.niehs.nih.gov/testing/status/agents/ts-10361-c.html>, (accessed July 1 2019).

11. Fassina, G., Abbondandolo, A., Mariani, L., Taningher, M., and Parodi, S. (1990) Mutagenicity in V79 cells does not correlate with carcinogenity in small rodents for 12 aromatic amines. *J Toxicol Environ Health*, **29**, 109-130.

12. Oberly, T.J., Rexroat, M.A., Bewsey, B.J., Richardson, K.K., and Michaelis, K.C. (1990) An evaluation of the CHO/HGPRT mutation assay involving suspension cultures and soft agar cloning: results for 33 chemicals. *Environmental and molecular mutagenesis*, **16**, 260-271.

13. Bitsch, A., Fecher, J., Jost, M., Klöhn, P.C., and Neumann, H.G. (1997) Genotoxic and Chronic Toxic Effects in the Carcinogenicity of Aromatic Amines. In Müller-Hermelink, H.K., Neumann, H.G. and Dekant, W. (eds.), *Risk and Progression Factors in Carcinogenesis*. Springer Berlin Heidelberg, Berlin, Heidelberg, pp. 209-223.

14. Heddle, J.A., Hite, M., Kirkhart, B., Mavournin, K., MacGregor, J.T., Newell, G.W., and Salamone, M.F. (1983) The induction of micronuclei as a measure of genotoxicity. A report of the U.S. Environmental Protection Agency Gene-Tox Program. *Mutation research*, **123**, 61-118.

15. Suzuki, H., Takasawa, H., Kobayashi, K., Terashima, Y., Shimada, Y., Ogawa, I., Tanaka, J., Imamura, T., Miyazaki, A., and Hayashi, M. (2009) Evaluation of a liver micronucleus assay with 12 chemicals using young rats (II): a study by the Collaborative Study Group for the Micronucleus Test/Japanese Environmental Mutagen Society-Mammalian Mutagenicity Study Group. *Mutagenesis*, **24**, 9-16.

16. Mirsalis, J.C., Tyson, C.K., and Butterworth, B.E. (1982) Detection of genotoxic carcinogens in the in vivo-in vitro hepatocyte DNA repair assay. *Environ Mutagen*, **4**, 553-562.

17. Madle, S., Dean, S.W., Andrae, U., Brambilla, G., Burlinson, B., Doolittle, D.J., Furihata, C., Hertner, T., McQueen, C.A., and Mori, H. (1994) Recommendations for the performance of UDS tests in vitro and in vivo. *Mutation research*, **312**, 263-285.

18. Ashby, J., and Beije, B. (1985) Concomitant observations of UDS in the liver and micronuclei in the bone marrow of rats exposed to cyclophosphamide or 2-acetylaminofluorene. *Mutation research*, **150**, 383-392.

19. Sasaki, Y.F., Nishidate, E., Izumiyama, F., Matsusaka, N., and Tsuda, S. (1997) Simple detection of chemical mutagens by the alkaline single-cell gel electrophoresis (Comet) assay in multiple mouse organs (liver, lung, spleen, kidney, and bone marrow). *Mutation research*, **391**, 215-231.

20. Sekihashi, K., Sasaki, T., Yamamoto, A., Kawamura, K., Ikka, T., Tsuda, S., and Sasaki, Y.F. (2001) A comparison of intraperitoneal and oral gavage administration in comet assay in mouse eight organs. *Mutation research*, **493**, 39-54.

21. Smith, C.C., Adkins, D.J., Martin, E.A., and O'Donovan, M.R. (2008) Recommendations for design of the rat comet assay. *Mutagenesis*, **23**, 233-240.

22. Bowen, D.E., Whitwell, J.H., Lillford, L., Henderson, D., Kidd, D., Mc Garry, S., Pearce, G., Beevers, C., and Kirkland, D.J. (2011) Evaluation of a multi-endpoint assay in rats, combining the bone-marrow micronucleus test, the Comet assay and the flow-cytometric peripheral blood micronucleus test. *Mutation research*, **722**, 7-19.

23. Kraynak, A.R., Barnum, J.E., Cunningham, C.L., Ng, A., Ykoruk, B.A., Bennet, B., Stoffregen, D., Merschman, M., Freeland, E., and Galloway, S.M. (2015) Alkaline comet assay in liver and stomach, and micronucleus assay in bone marrow, from rats treated with 2-acetylaminofluorene, azidothymidine, cisplatin, or isobutyraldehyde. *Mutat Res Genet Toxicol Environ Mutagen*, **786-788**, 77-86.

24. Hobbs, C.A., Recio, L., Streicker, M., Boyle, M.H., Tanaka, J., Shiga, A., and Witt, K.L. (2015) Comet assay evaluation of six chemicals of known genotoxic potential in rats. *Mutat Res Genet Toxicol Environ Mutagen*, **786-788**, 172-181.

25. Uno, Y., Kojima, H., Omori, T., Corvi, R., Honma, M., Schechtman, L.M., Tice, R.R., Beevers, C., De Boeck, M., Burlinson, B., Hobbs, C.A., Kitamoto, S., Kraynak, A.R., McNamee, J., Nakagawa, Y., Pant, K., Plappert-Helbig, U., Priestley, C., Takasawa, H., Wada, K., Wirnitzer, U., Asano, N., Escobar, P.A., Lovell, D., Morita, T., Nakajima, M., Ohno, Y., and Hayashi, M. (2015) JaCVAM-organized international validation study of the in vivo rodent alkaline comet assay for detection of genotoxic carcinogens: II. Summary of definitive validation study results. *Mutat Res Genet Toxicol Environ Mutagen*, **786-788**, 45-76.

26. Sawada, S., Yamanaka, T., Yamatsu, K., Furihata, C., and Matsushima, T. (1991) Chromosome aberrations, micronuclei and sister-chromatid exchanges (SCEs) in rat liver induced in vivo by hepatocarcinogens including heterocyclic amines. *Mutation research*, **251**, 59-69.

27. Brooks, T.M., and Dean, S.W. (1996) Detection of gene mutation in skin, stomach and liver of MutaMouse following oral or topical treatment with N-methyl-N'-nitro-N-nitrosoguanidine or 1-chloromethylpyrene: some preliminary observations. *Mutagenesis*, **11**, 529-532.

28. Ross, J.A., Nelson, G.B., Wilson, K.H., Rabinowitz, J.R., Galati, A., Stoner, G.D., Nesnow, S., and Mass, M.J. (1995) Adenomas induced by polycyclic aromatic hydrocarbons in strain A/J mouse lung correlate with time-integrated DNA adduct levels. *Cancer Res*, **55**, 1039-1044.

29. Lambert, I.B., Singer, T.M., Boucher, S.E., and Douglas, G.R. (2005) Detailed review of transgenic rodent mutation assays. *Mutation research*, **590**, 1-280.

30. McCann, J., Choi, E., Yamasaki, E., and Ames, B.N. (1975) Detection of carcinogens as mutagens in the Salmonella/microsome test: assay of 300 chemicals. *Proc Natl Acad Sci U S A*, **72**, 5135-5139.

31. Simmon, V.F., Rosenkranz, H.S., Zeiger, E., and Poirier, L.A. (1979) Mutagenic activity of chemical carcinogens and related compounds in the intraperitoneal host-mediated assay. *J Natl Cancer Inst*, **62**, 911-918.

32. Massey, E.D., and Hinchliffe, S. (2010) 2-Aminoanthracene, diethylstilboestrol and vinblastine tested in the in vitro mammalian cell micronucleus test at British American Tobacco UK in support of the OECD draft Test Guideline 487. *Mutation research*, **702**, 208-211.

33. Baker, R.S., Mitchell, G.A., Meher-Homji, K.M., and Podobna, E. (1983) Sensitivity of two Chinese hamster cell lines to SCE induction by a variety of chemical mutagens. *Mutation research*, **118**, 103-116.

34. NTP Testing Status of 2-Aminoanthracene 10912-X. National Toxicology Program [web site], <https://ntp.niehs.nih.gov/testing/status/agents/ts-10912-x.html>, (accessed July 1 2019).

35. Mortelmans, K., Haworth, S., Lawlor, T., Speck, W., Tainer, B., and Zeiger, E. (1986) Salmonella mutagenicity tests: II. Results from the testing of 270 chemicals. *Environ Mutagen*, **8 Suppl 7**, 1-119.

36. NTP Testing Status of 2-Chloroethyltrimethylammonium chloride 10669-Y. National Toxicology Program [web site], <https://ntp.niehs.nih.gov/testing/status/agents/ts-10669-y.html>, (accessed July 1 2019).

37. Morita, T., Asano, N., Awogi, T., Sasaki, Y.F., Sato, S., Shimada, H., Sutou, S., Suzuki, T., Wakata, A., Sofuni, T., and Hayashi, M. (1997) Evaluation of the rodent micronucleus assay in the screening of IARC carcinogens (groups 1, 2A and 2B) the summary report of the 6th collaborative study by CSGMT/JEMS MMS. Collaborative Study of the Micronucleus Group Test. Mammalian Mutagenicity Study Group. *Mutation research*, **389**, 3-122.

38. Zeiger, E. (1977) Genotoxicity database. *Handbook of carcinogenic potency and genotoxicity databases*. CRC Press Inc., Boca Raton, pp. 687-729.

39. EPA Summary of Toxicology data on chlormequat chloride. California EPA Department of Pesticide Regulation Medical Toxicology Branch. Toxicology Data Review Summaries. Original Document 12-24-2002. Information retrieved afrom <http://www.cdpr.ca.gov/docs/risk/toxsums/toxsumlist.htm>.

40. Schisler, M., Bhaskar Gollapudi, B., and M. Moore, M. (2010) *Evaluation of the Mouse Lymphoma Mutation Assay (MLA) Data of the U. S. National Toxicology Program (NTP) Using International Workshop on Genotoxicity,Tests (IWGT) Criteria*.

41. Slesinski, R.S., Guzzie, P.J., Putman, D.L., and Ballantyne, B. (1988) In vitro and in vivo evaluation of the genotoxic potential of 2-ethyl-1,3-hexanediol. *Toxicology*, **53**, 179-198.

42. ECHA 2-ethylhexane-1,3-diol (2-Ethyl-1,3-hexandiol) dossier ECHA database [web site] <https://echa.europa.eu/registration-dossier/-/registered-dossier/13598/7/7/2> (accessed July 1 2019).

43. NTP Testing Status of 2-Ethyl-1,3-hexanediol M930037. National Toxicology Program [web site], <https://ntp.niehs.nih.gov/testing/status/agents/ts-m930037.html>, (accessed July 1 2019).

44. Fowler, P., Smith, K., Young, J., Jeffrey, L., Kirkland, D., Pfuhler, S., and Carmichael, P. (2012) Reduction of misleading ("false") positive results in mammalian cell genotoxicity assays. I. Choice of cell type. *Mutation research*, **742**, 11-25.

45. Seifried, H.E., Seifried, R.M., Clarke, J.J., Junghans, T.B., and San, R.H. (2006) A compilation of two decades of mutagenicity test results with the Ames Salmonella typhimurium and L5178Y mouse lymphoma cell mutation assays. *Chem Res Toxicol*, **19**, 627-644.

46. Renner, H.W., and Munzner, R. (1980) Mutagenicity of sulphonylureas. *Mutation research*, **77**, 349-355.

47. Zeiger, E., Anderson, B., Haworth, S., Lawlor, T., and Mortelmans, K. (1988) Salmonella mutagenicity tests: IV. Results from the testing of 300 chemicals. *Environmental and molecular mutagenesis*, **11 Suppl 12**, 1-157.

48. Ames, B.N., Kammen, H.O., and Yamasaki, E. (1975) Hair dyes are mutagenic: identification of a variety of mutagenic ingredients. *Proc Natl Acad Sci U S A*, **72**, 2423-2427.

49. Shahin, M.M., Chopy, C., and Lequesne, N. (1985) Comparisons of mutation induction by six monocyclic aromatic amines in Salmonella typhimurium tester strains TA97, TA1537, and TA1538. *Environ Mutagen*, **7**, 535-546.

50. Cunningham, M.L., and Matthews, H.B. (1990) Evidence for an acetoxyarylamine as the ultimate mutagenic reactive intermediate of the carcinogenic aromatic amine 2,4-diaminotoluene. *Mutation research*, **242**, 101-110.

51. Severin, I., Jondeau, A., Dahbi, L., and Chagnon, M.C. (2005) 2,4-Diaminotoluene (2,4-DAT)-induced DNA damage, DNA repair and micronucleus formation in the human hepatoma cell line HepG2. *Toxicology*, **213**, 138-146.

52. Loveday, K.S., Anderson, B.E., Resnick, M.A., and Zeiger, E. (1990) Chromosome aberration and sister chromatid exchange tests in Chinese hamster ovary cells in vitro. V: Results with 46 chemicals. *Environmental and molecular mutagenesis*, **16**, 272-303.

53. NTP Testing Status of 2,4-Diaminotoluene (2,4-toluene diamine) 10463-P. National Toxicology Program [web site], <https://ntp.niehs.nih.gov/testing/status/agents/ts-10463-p.html>, (accessed July 1 2019).

54. Selden, J.R., Dolbeare, F., Clair, J.H., Miller, J.E., McGettigan, K., DiJohn, J.A., Dysart, G.R., and DeLuca, J.G. (1994) Validation of a flow cytometric in vitro DNA repair (UDS) assay in rat hepatocytes. *Mutation research*, **315**, 147-167.

55. Coppinger, W.J., Brennan, S.A., Carver, J.H., and Thompson, E.D. (1984) Locus specificity of mutagenicity of 2,4-diaminotoluene in both L5178Y mouse lymphoma and AT3-2 Chinese hamster ovary cells. *Mutation research*, **135**, 115-123.

56. Mitchell, A.D., Auletta, A.E., Clive, D., Kirby, P.E., Moore, M.M., and Myhr, B.C. (1997) The L5178Y/tk+/- mouse lymphoma specific gene and chromosomal mutation assay a phase III report of the U.S. Environmental Protection Agency Gene-Tox Program. *Mutation research*, **394**, 177-303.

57. Armstrong, M.J., Bean, C.L., and Galloway, S.M. (1992) A quantitative assessment of the cytotoxicity associated with chromosomal aberration detection in Chinese hamster ovary cells. *Mutation research*, **265**, 45-60.

58. George, E., and Westmoreland, C. (1991) Evaluation of the in vivo genotoxicity of the structural analogues 2,6-diaminotoluene and 2,4-diaminotoluene using the rat micronucleus test and rat liver UDS assay. *Carcinogenesis*, **12**, 2233-2237.

59. Sasaki, Y.F., Fujikawa, K., Ishida, K., Kawamura, N., Nishikawa, Y., Ohta, S., Satoh, M., Madarame, H., Ueno, S., Susa, N., Matsusaka, N., and Tsuda, S. (1999) The alkaline single cell gel electrophoresis assay with mouse multiple organs: results with 30 aromatic amines evaluated by the IARC and U.S. NTP. *Mutation research*, **440**, 1-18.

60. De Boeck, M., van der Leede, B.J., De Vlieger, K., Geys, H., Vynckier, A., and Van Gompel, J. (2015) Evaluation of p-phenylenediamine, o-phenylphenol sodium salt, and 2,4-diaminotoluene in the rat comet assay as part of the Japanese Center for the Validation of Alternative Methods (JaCVAM)-initiated international validation study of in vivo rat alkaline comet assay. *Mutat Res Genet Toxicol Environ Mutagen*, **786-788**, 151-157.

61. Suter, W., Ahiabor, R., Blanco, B., Locher, F., Mantovani, F., Robinson, M., Sreenan, G., Staedtler, F., Swingler, T., Vignutelli, A., and Perentes, E. (1996) Evaluation of the in vivo genotoxic potential of three carcinogenic aromatic amines using the Big Blue transgenic mouse mutation assay. *Environmental and molecular mutagenesis*, **28**, 354-362.

62. Kirkland, D., and Beevers, C. (2006) Induction of LacZ mutations in Muta Mouse can distinguish carcinogenic from non-carcinogenic analogues of diaminotoluenes and nitronaphthalenes. *Mutation research*, **608**, 88-96.

63. Haworth, S., Lawlor, T., Mortelmans, K., Speck, W., and Zeiger, E. (1983) Salmonella mutagenicity test results for 250 chemicals. *Environ Mutagen*, **5 Suppl 1**, 1-142.

64. Anderson, B.E., Zeiger, E., Shelby, M.D., Resnick, M.A., Gulati, D.K., Ivett, J.L., and Loveday, K.S. (1990) Chromosome aberration and sister chromatid exchange test results with 42 chemicals. *Environmental and molecular mutagenesis*, **16 Suppl 18**, 55-137.

65. Probst, G.S., McMahon, R.E., Hill, L.E., Thompson, C.Z., Epp, J.K., and Neal, S.B. (1981) Chemically-induced unscheduled DNA synthesis in primary rat hepatocyte cultures: a comparison with bacterial mutagenicity using 218 compounds. *Environ Mutagen*, **3**, 11-32.

66. Myhr, B., McGregor, D., Bowers, L., Riach, C., Brown, A.G., Edwards, I., McBride, D., Martin, R., and Caspary, W.J. (1990) L5178Y mouse lymphoma cell mutation assay results with 41 compounds. *Environmental and molecular mutagenesis*, **16 Suppl 18**, 138-167.

67. Hilliard, C.A., Armstrong, M.J., Bradt, C.I., Hill, R.B., Greenwood, S.K., and Galloway, S.M. (1998) Chromosome aberrations in vitro related to cytotoxicity of nonmutagenic chemicals and metabolic poisons. *Environmental and molecular mutagenesis*, **31**, 316-326.

68. Jansson, K., and Jansson, V. (1986) Inability of chlorophenols to induce 6-thioguanine-resistant mutants in V79 Chinese hamster cells. *Mutation research*, **171**, 165-168.

69. Amer, S.M., and Aly, F.A. (2001) Genotoxic effect of 2,4-dichlorophenoxy acetic acid and its metabolite 2,4-dichlorophenol in mouse. *Mutation research*, **494**, 1-12.

70. Sasaki, Y.F., Sekihashi, K., Izumiyama, F., Nishidate, E., Saga, A., Ishida, K., and Tsuda, S. (2000) The comet assay with multiple mouse organs: comparison of comet assay results and carcinogenicity with 208 chemicals selected from the IARC monographs and U.S. NTP Carcinogenicity Database. *Crit Rev Toxicol*, **30**, 629-799.

71. Brusick, D.J., Simmon, V.F., Rosenkranz, H.S., Ray, V.A., and Stafford, R.S. (1980) An evaluation of the Escherichia coli WP2 and WP2 uvrA reverse mutation assay. *Mutation research*, **76**, 169-190.

72. Rinkus, S.J., and Legator, M.S. (1979) Chemical characterization of 465 known or suspected carcinogens and their correlation with mutagenic activity in the Salmonella typhimurium system. *Cancer Res*, **39**, 3289-3318.

73. O'Donovan, M.R. (1990) The comparative responses of Salmonella typhimurium TA1537 and TA97a to a range of reference mutagens and novel compounds. *Mutagenesis*, **5**, 267-274.

74. Diehl, M., and Fort, F. (1996) Spiral Salmonella assay: validation against the standard pour-plate assay. *Environmental and molecular mutagenesis*, **27**, 227-236.

75. Nesslany, F., and Marzin, D. (1999) A micromethod for the in vitro micronucleus assay. *Mutagenesis*, **14**, 403-410.

76. Fellows, M.D., and O'Donovan, M.R. (2007) Cytotoxicity in cultured mammalian cells is a function of the method used to estimate it. *Mutagenesis*, **22**, 275-280.

77. Scolastici, C., Alves de Lima, R.O., Barbisan, L.F., Ferreira, A.L., Ribeiro, D.A., and Salvadori, D.M. (2007) Lycopene activity against chemically induced DNA damage in Chinese hamster ovary cells. *Toxicology in vitro : an international journal published in association with BIBRA*, **21**, 840-845.

78. Murli, H., Galloway, S.M., Ivett, J.L., Parry, D.M., and Mulvihill, J.J. (1987) Baseline and mutagen-induced sister-chromatid exchanges in cultures of human whole blood and purified fresh or frozen lymphocytes. *Mutation research*, **180**, 101-108.

79. NTP Testing Status of 4-Nitroquinoline-N-oxide 10365-S. National Toxicology Program [web site], <https://ntp.niehs.nih.gov/whatwestudy/testpgm/status/ts-10365-s.html>, (accessed July 1 2019).

80. Valentin-Severin, I., Thybaud, V., Le Bon, A.M., Lhuguenot, J.C., and Chagnon, M.C. (2004) The autoradiographic test for unscheduled DNA synthesis: a sensitive assay for the detection of DNA repair in the HepG2 cell line. *Mutation research*, **559**, 211-217.

81. Homme, M., Jacobi, H., Juhl-Strauss, U., and Witte, I. (2000) Synergistic DNA damaging effects of 4-nitroquinoline-1-oxide and non-effective concentrations of methyl methanesulfonate in human fibroblasts. *Mutation research*, **461**, 211-219.

82. Wangenheim, J., and Bolcsfoldi, G. (1988) Mouse lymphoma L5178Y thymidine kinase locus assay of 50 compounds. *Mutagenesis*, **3**, 193-205.

83. Preston, R.J., Au, W., Bender, M.A., Brewen, J.G., Carrano, A.V., Heddle, J.A., McFee, A.F., Wolff, S., and Wassom, J.S. (1981) Mammalian in vivo and in vitro cytogenetic assays: a report of the U.S. EPA's gene-tox program. *Mutation research*, **87**, 143-188.

84. Gupta, R.S., and Singh, B. (1982) Mutagenic responses of five independent genetic loci in CHO cells to a variety of mutagens. Development and characteristics of a mutagen screening system based on selection for multiple drug-resistant markers. *Mutation research*, **94**, 449-466.

85. Hsie AW, O.J., Machanoff R, Schenley RL, Brimer PA (1981) Screening for mutagenic response of four coded chemicals by the CHO/HGPRT system, In: Evaluation of short-term tests for carcinogens: Report of the International Collaborative Program. *Prog Mutat Res*, **1**, 602-607.

86. Nishi, Y., Hasegawa, M.M., Taketomi, M., Ohkawa, Y., and Inui, N. (1984) Comparison of 6-thioguanine-resistant mutation and sister chromatid exchanges in Chinese hamster V79 cells with forty chemical and physical agents. *Cancer Res*, **44**, 3270-3279.

87. Nakajima, M., Kikuchi, M., Saeki, K., Miyata, Y., Terada, M., Kishida, F., Yamamoto, R., Furihata, C., and Dean, S.W. (1999) Mutagenicity of 4-nitroquinoline 1-oxide in the MutaMouse. *Mutation research*, **444**, 321-336.

88. Sato, S., Takizawa, H., and Inui, N. (1990) A comparison of micronucleus induction in 3 mouse strains with representative clastogens. *Toxicology letters*, **52**, 215-220.

89. Trzos, R.J., Petzold, G.L., Brunden, M.N., and Swenberg, J.A. (1978) The evaluation of sixteen carcinogens in the rat using the micronucleus test. *Mutation research*, **58**, 79-86.

90. Carvalho, J.G., Noguti, J., da Silva, V.H., Dedivitis, R.A., Franco, M., and Ribeiro, D.A. (2012) Alkylation-induced genotoxicity as a predictor of DNA repair deficiency following experimental oral carcinogenesis. *J Mol Histol*, **43**, 145-150.

91. Miranda, S.R., Noguti, J., Carvalho, J.G., Oshima, C.T., and Ribeiro, D.A. (2011) Oxidative DNA damage is a preliminary step during rat tongue carcinogenesis induced by 4-nitroquinoline 1-oxide. *J Mol Histol*, **42**, 181-186.

92. Ribeiro, D.A., Bazo, A.P., da Silva Franchi, C.A., Marques, M.E., and Salvadori, D.M. (2004) Chlorhexidine induces DNA damage in rat peripheral leukocytes and oral mucosal cells. *J Periodontal Res*, **39**, 358-361.

93. Ribeiro, D.A., Favero Salvadori, D.M., da Silva, R.N., Ribeiro Darros, B., and Alencar Marques, M.E. (2004) Genomic instability in non-neoplastic oral mucosa cells can predict risk during 4-nitroquinoline 1-oxide-induced rat tongue carcinogenesis. *Oral Oncol*, **40**, 910-915.

94. Ribeiro, D.A., Grilli, D.G., and Salvadori, D.M. (2008) Genomic instability in blood cells is able to predict the oral cancer risk: an experimental study in rats. *J Mol Histol*, **39**, 481-486.

95. Stankowski, L.F., Jr., Roberts, D.J., Chen, H., Lawlor, T., McKeon, M., Murli, H., Thakur, A., and Xu, Y. (2011) Integration of Pig-a, micronucleus, chromosome aberration, and Comet assay endpoints in a 28-day rodent toxicity study with 4-nitroquinoline-1-oxide. *Environmental and molecular mutagenesis*, **52**, 738-747.

96. Toyoizumi, T., Ohta, R., Kawakami, K., Nakagawa, Y., Tazura, Y., Kuwagata, M., Noguchi, S., Sui, H., and Yamakage, K. (2012) Usefulness of combined in vivo skin comet assay and in vivo skin micronucleus test. *Mutation research*, **743**, 42-51.

97. McFee, A.F. (1989) Genotoxic potency of three quinoline compounds evaluated in vivo in mouse marrow cells. *Environmental and molecular mutagenesis*, **13**, 325-331.

98. McFee, A.F., and Tice, R.R. (1990) Influence of treatment to sacrifice time and the presence of BrdUrd on chemically-induced aberration rates in mouse marrow cells. *Mutation research*, **241**, 95-108.

99. Young, R.R., Thompson, C.M., Dinesdurage, H.R., Elbekai, R.H., Suh, M., Rohr, A.C., and Proctor, D.M. (2015) A robust method for assessing chemically induced mutagenic effects in the oral cavity of transgenic Big Blue(R) rats. *Environmental and molecular mutagenesis*, **56**, 629-636.

100. Horibata, K., Ukai, A., Kimoto, T., Suzuki, T., Kamoshita, N., Masumura, K., Nohmi, T., and Honma, M. (2013) Evaluation of in vivo genotoxicity induced by N-ethyl-N-nitrosourea, benzo[a]pyrene, and 4-nitroquinoline-1-oxide in the Pig-a and gpt assays. *Environmental and molecular mutagenesis*, **54**, 747-754.

101. Suzuki, T., Itoh, S., Nakajima, M., Hachiya, N., and Hara, T. (1999) Target organ and time-course in the mutagenicity of five carcinogens in MutaMouse: a summary report of the second collaborative study of the transgenic mouse mutation assay by JEMS/MMS. *Mutation research*, **444**, 259-268.

102. NTP (1989) Toxicology and Carcinogenesis Studies of 4-Vinyl-1-cyclohexene Diepoxide (CAS No. 106-87-6) in F344/N Rats and B6C3F1 Mice (Dermal Studies). *Natl Toxicol Program Tech Rep Ser.*, **362**, 1-249.

103. IARC (1994) 4-Vinyl-1-cyclohexene diepoxide. *IARC Working Group on the Evaluation of Carcinogenic Risk to Humans. Some Industrial Chemicals*, <https://monographs.iarc.fr/wp-content/uploads/2018/06/mono60-14.pdf>, pp. 361-372.

104. McGregor, D.B., Brown, A., Cattanach, P., Edwards, I., McBride, D., and Caspary, W.J. (1988) Responses of the L5178Y tk+/tk- mouse lymphoma cell forward mutation assay. II: 18 coded chemicals. *Environmental and molecular mutagenesis*, **11**, 91-118.

105. Turchi, G., Bonatti, S., Citti, L., Gervasi, P.G., Abbondandolo, A., and Presciuttini, S. (1981) Alkylating properties and genetic activity of 4-vinylcyclohexene metabolites and structurally related expoxides. *Mutation Research/Fundamental and Molecular Mechanisms of Mutagenesis*, **83**, 419-430.

106. Prival, M.J., Simmon, V.F., and Mortelmans, K.E. (1991) Bacterial mutagenicity testing of 49 food ingredients gives very few positive results. *Mutation research*, **260**, 321-329.

107. Zeiger, E., Anderson, B., Haworth, S., Lawlor, T., Mortelmans, K., and Speck, W. (1987) Salmonella mutagenicity tests: III. Results from the testing of 255 chemicals. *Environ Mutagen*, **9 Suppl 9**, 1-109.

108. Watanabe, K., Sasaki, T., and Kawakami, K. (1998) Comparisons of chemically-induced mutation among four bacterial strains, Salmonella typhimurium TA102 and TA2638, and Escherichia coli WP2/pKM101 and WP2 uvrA/pKM101: collaborative study III and evaluation of the usefulness of these strains. *Mutation research*, **416**, 169-181.

109. Cariou, O., Laroche-Prigent, N., Ledieu, S., Guizon, I., Paillard, F., and Thybaud, V. (2010) Cytosine arabinoside, vinblastine, 5-fluorouracil and 2-aminoanthracene testing in the in vitro micronucleus assay with L5178Y mouse lymphoma cells at Sanofi Aventis, with different cytotoxicity measurements, in support of the draft OECD Test Guideline on In Vitro Mammalian Cell Micronucleus Test. *Mutation research*, **702**, 148-156.

110. Whitwell, J., Fowler, P., Allars, S., Jenner, K., Lloyd, M., Wood, D., Smith, K., Young, J., Jeffrey, L., and Kirkland, D. (2010) 5-Fluorouracil, colchicine, benzo[a]pyrene and cytosine arabinoside tested in the in vitro mammalian cell micronucleus test (MNvit) in Chinese hamster V79 cells at Covance Laboratories, Harrogate, UK in support of OECD draft Test Guideline 487. *Mutation research*, **702**, 230-236.

111. Kirkland, D. (2010) Evaluation of different cytotoxic and cytostatic measures for the in vitro micronucleus test (MNVit): summary of results in the collaborative trial. *Mutation research*, **702**, 139-147.

112. Lorge, E., Thybaud, V., Aardema, M.J., Oliver, J., Wakata, A., Lorenzon, G., and Marzin, D. (2006) SFTG international collaborative study on in vitro micronucleus test I. General conditions and overall conclusions of the study. *Mutation research*, **607**, 13-36.

113. Oka, H., Ouchida, M., Kondo, T., Morita, F., and Shimizu, K. (2012) Different responses to 5-fluoraouracil in mutagenicity and gene expression between two human lymphoblastoid cell lines with or without TP53 mutation. *Acta Med Okayama*, **66**, 119-129.

114. Sofuni, T., Honma, M., Hayashi, M., Shimada, H., Tanaka, N., Wakuri, S., Awogi, T., Yamamoto, K.I., Nishi, Y., and Nakadate, M. (1996) Detection of in vitro clastogens and spindle poisons by the mouse lymphoma assay using the microwell method: interim report of an international collaborative study. *Mutagenesis*, **11**, 349-355.

115. Zhou, C., Zhang, M., Huang, P., Tu, H., Wang, Z., Dertinger, S.D., Torous, D.K., and Chang, Y. (2014) Assessment of 5-fluorouracil and 4-nitroquinoline-1-oxide in vivo genotoxicity with Pig-a mutation and micronucleus endpoints. *Environmental and molecular mutagenesis*, **55**, 735-740.

116. Wakata, A., Miyamae, Y., Sato, S., Suzuki, T., Morita, T., Asano, N., Awogi, T., Kondo, K., and Hayashi, M. (1998) Evaluation of the rat micronucleus test with bone marrow and peripheral blood: summary of the 9th collaborative study by CSGMT/JEMS. MMS. Collaborative Study Group for the Micronucleus Test. Environmental Mutagen Society of Japan. Mammalian Mutagenicity Study Group. *Environmental and molecular mutagenesis*, **32**, 84-100.

117. Higashikuni, N., and Sutou, S. (1995) An optimal, generalized sampling time of 30 +/- 6 h after double dosing in the mouse peripheral blood micronucleus test. *Mutagenesis*, **10**, 313-319.

118. Ohuchida, A., Furukawa, A., Yoshida, J., Watanabe, M., Aruga, F., Miwa, Y., Shinkawa, K., and Kinae, N. (1992) Micronucleus assays on 5-fluorouracil and 6-mercaptopurine with mouse peripheral blood reticulocytes. *Mutation research*, **278**, 139-143.

119. Plappert-Helbig, U., Junker-Walker, U., and Martus, H.J. (2015) Evaluation of methyl methanesulfonate, 2,6-diaminotoluene and 5-fluorouracil: Part of the Japanese center for the validation of alternative methods (JaCVAM) international validation study of the in vivo rat alkaline comet assay. *Mutat Res Genet Toxicol Environ Mutagen*, **786-788**, 120-124.

120. NTP Testing Status of 8-Hydroxyquinoline 10598-N. National Toxicology Program [web site], <https://ntp.niehs.nih.gov/whatwestudy/testpgm/status/ts-10598-n.html>, (accessed July 1 2019).

121. Hamoud, M.A., Ong, T., Petersen, M., and Nath, J. (1989) Effects of quinoline and 8-hydroxyquinoline on mouse bone marrow erythrocytes as measured by the micronucleus assay. *Teratog Carcinog Mutagen*, **9**, 111-118.

122. PubChem HSDB 8-Hydroxyquinoline. Retrieved from [web site]: <https://pubchem.ncbi.nlm.nih.gov/source/hsdb/4073>.

123. Ashby, J., Mohammed, R., Lefevre, P.A., and Bandara, L. (1989) Quinoline: unscheduled DNA synthesis and mitogenesis data from the rat liver in vivo. *Environmental and molecular mutagenesis*, **14**, 221-228.

124. Galloway, S.M., Armstrong, M.J., Reuben, C., Colman, S., Brown, B., Cannon, C., Bloom, A.D., Nakamura, F., Ahmed, M., Duk, S., and et al. (1987) Chromosome aberrations and sister chromatid exchanges in Chinese hamster ovary cells: evaluations of 108 chemicals. *Environmental and molecular mutagenesis*, **10 Suppl 10**, 1-175.

125. McGregor, D.B., Brown, A.G., Howgate, S., McBride, D., Riach, C., and Caspary, W.J. (1991) Responses of the L5178Y mouse Lymphoma cell forward mutation assay. V: 27 coded chemicals. *Environmental and molecular mutagenesis*, **17**, 196-219.

126. Ress, N.B., Witt, K.L., Xu, J., Haseman, J.K., and Bucher, J.R. (2002) Micronucleus induction in mice exposed to diazoaminobenzene or its metabolites, benzene and aniline: implications for diazoaminobenzene carcinogenicity. *Mutation research*, **521**, 201-208.

127. Witt, K.L., Knapton, A., Wehr, C.M., Hook, G.J., Mirsalis, J., Shelby, M.D., and MacGregor, J.T. (2000) Micronucleated erythrocyte frequency in peripheral blood of B6C3F(1) mice from short-term, prechronic, and chronic studies of the NTP carcinogenesis bioassay program. *Environmental and molecular mutagenesis*, **36**, 163-194.

128. Dunkel, V.C., Zeiger, E., Brusick, D., McCoy, E., McGregor, D., Mortelmans, K., Rosenkranz, H.S., and Simmon, V.F. (1984) Reproducibility of microbial mutagenicity assays: I. Tests with Salmonella typhimurium and Escherichia coli using a standardized protocol. *Environ Mutagen*, **6 Suppl 2**, 1-251.

129. Fowler, P., Whitwell, J., Jeffrey, L., Young, J., Smith, K., and Kirkland, D. (2010) Cadmium chloride, benzo[a]pyrene and cyclophosphamide tested in the in vitro mammalian cell micronucleus test (MNvit) in the human lymphoblastoid cell line TK6 at Covance laboratories, Harrogate UK in support of OECD draft Test Guideline 487. *Mutation research*, **702**, 171-174.

130. NTP Testing Status of Benzo(a)pyrene 10353-C. National Toxicology Program [web site], <https://ntp.niehs.nih.gov/testing/status/agents/ts-10353-c.html>, (accessed July 1 2019).

131. Mitchell, A.D., Rudd, C.J., and Caspary, W.J. (1988) Evaluation of the L5178Y mouse lymphoma cell mutagenesis assay: intralaboratory results for sixty-three coded chemicals tested at SRI International. *Environmental and molecular mutagenesis*, **12 Suppl 13**, 37-101.

132. PubChem National Center for Biotechnology Information. Benzo[a]pyrene, CID=2336, PubChem Online Database <https://pubchem.ncbi.nlm.nih.gov/compound/Benzo_a_pyrene> (accessed on Jan. 13, 2020).

133. Mavournin, K.H., Blakey, D.H., Cimino, M.C., Salamone, M.F., and Heddle, J.A. (1990) The in vivo micronucleus assay in mammalian bone marrow and peripheral blood. A report of the U.S. Environmental Protection Agency Gene-Tox Program. *Mutation research*, **239**, 29-80.

134. Puri, E.C., and Muller, D. (1989) Testing of hydralazine in in vivo-in vitro hepatocyte assays for UDS and stimulation of replicative DNA synthesis. *Mutation research*, **218**, 13-19.

135. Vrzoc, M., and Petras, M.L. (1997) Comparison of alkaline single cell gel (Comet) and peripheral blood micronucleus assays in detecting DNA damage caused by direct and indirect acting mutagens. *Mutation Research/Fundamental and Molecular Mechanisms of Mutagenesis*, **381**, 31-40.

136. Garry, S., Nesslany, F., Aliouat, E., Haguenoer, J.M., and Marzin, D. (2003) Assessment of genotoxic effect of benzo[a]pyrene in endotracheally treated rat using the comet assay. *Mutation research*, **534**, 33-43.

137. Garry, S., Nesslany, F., Aliouat, E., Haguenoer, J.M., and Marzin, D. (2003) Hematite (Fe(2)O(3)) enhances benzo[a]pyrene genotoxicity in endotracheally treated rat, as determined by Comet Assay. *Mutation research*, **538**, 19-29.

138. Gradecka-Meesters, D., Palus, J., Prochazka, G., Segerback, D., Dziubaltowska, E., Kotova, N., Jenssen, D., Arkusz, J., Lundin, C., Vikstrom, E., Rydzynski, K., Nilsson, R., and Stepnik, M. (2011) Assessment of the protective effects of selected dietary anticarcinogens against DNA damage and cytogenetic effects induced by benzo[a]pyrene in C57BL/6J mice. *Food Chem Toxicol*, **49**, 1674-1683.

139. Jin, N.Z., Zhu, Y.P., Zhou, J.W., Mao, L., Zhao, R.C., Fang, T.H., and Wang, X.R. (2006) Preventive effects of quercetin against benzo[a]pyrene-induced DNA damages and pulmonary precancerous pathologic changes in mice. *Basic Clin Pharmacol Toxicol*, **98**, 593-598.

140. Paul, S., Bhattacharyya, S.S., Samaddar, A., Boujedaini, N., and Khuda-Bukhsh, A.R. (2011) Anticancer potentials of root extract of Polygala senega against benzo[a]pyrene-induced lung cancer in mice. *Zhong Xi Yi Jie He Xue Bao*, **9**, 320-327.

141. Yu, R.A., Li, X.Y., Lu, W.Q., Mei, Y.H., Zhu, J.L., Chen, X.N., and Chen, X.M. (2004) [Effects of selenium on benzo[a] pyrene-induce DNA damage in mouse lung cells]. *Zhonghua Lao Dong Wei Sheng Zhi Ye Bing Za Zhi*, **22**, 445-447.

142. Vaghef, H., Wisen, A.C., and Hellman, B. (1996) Demonstration of benzo(a)pyrene-induced DNA damage in mice by alkaline single cell gel electrophoresis: evidence for strand breaks in liver but not in lymphocytes and bone marrow. *Pharmacol Toxicol*, **78**, 37-43.

143. Rothfuss, A., O'Donovan, M., De Boeck, M., Brault, D., Czich, A., Custer, L., Hamada, S., Plappert-Helbig, U., Hayashi, M., Howe, J., Kraynak, A.R., van der Leede, B.J., Nakajima, M., Priestley, C., Thybaud, V., Saigo, K., Sawant, S., Shi, J., Storer, R., Struwe, M., Vock, E., and Galloway, S. (2010) Collaborative study on fifteen compounds in the rat-liver Comet assay integrated into 2- and 4-week repeat-dose studies. *Mutation research*, **702**, 40-69.

144. Beal, M.A., Gagne, R., Williams, A., Marchetti, F., and Yauk, C.L. (2015) Characterizing Benzo[a]pyrene-induced lacZ mutation spectrum in transgenic mice using next-generation sequencing. *BMC Genomics*, **16**, 812.

145. O'Brien, J.M., Beal, M.A., Yauk, C.L., and Marchetti, F. (2016) Benzo(a)pyrene Is Mutagenic in Mouse Spermatogonial Stem Cells and Dividing Spermatogonia. *Toxicol Sci*, **152**, 363-371.

146. de Vries, A., Dolle, M.E., Broekhof, J.L., Muller, J.J., Kroese, E.D., van Kreijl, C.F., Capel, P.J., Vijg, J., and van Steeg, H. (1997) Induction of DNA adducts and mutations in spleen, liver and lung of XPA-deficient/lacZ transgenic mice after oral treatment with benzo[a]pyrene: correlation with tumour development. *Carcinogenesis*, **18**, 2327-2332.

147. IARC (1993) Cadmium and cadmium compounds. *IARC Working Group on the Evaluation of Carcinogenic Risk to Humans. Beryllium, Cadmium, Mercury, and Exposures in the Glass Manufacturing Industry*, <https://monographs.iarc.fr/wp-content/uploads/2018/06/mono58-7.pdf>, pp. 119-237.

148. Meintieres, S., Biola, A., Pallardy, M., and Marzin, D. (2003) Using CTLL-2 and CTLL-2 bcl2 cells to avoid interference by apoptosis in the in vitro micronucleus test. *Environmental and molecular mutagenesis*, **41**, 14-27.

149. National Toxicology Program Testing Status of Curcumin 11612-S.

150. Lederer, J., Collin, J.P., Pottier-Arnould, A.M., and Gondry, E. (1971) [Cytogenetic and teratogenic action of cyclamate and its metabolites]. *Therapeutique*, **47**, 357-363.

151. Collin, J.P. (1971) Cytogenetic effect of sodium cyclamate, cyclohexanone and cyclohexanol. *Diabete*, **19**, 215-221.

152. Dyshlovoi, V.D., Boiko, N.L., Shemetun, A.M., and Kharchenko, T.I. (1981) Cytogenetic action of cyclohexanone. *Gig Sanit*, 76-77.

153. ECHA Cyclohexanone dossier. ECHA database [website] <https://echa.europa.eu/registration-dossier/-/registered-dossier/15388> (accessed 7 July 2019).

154. De Hondt, H.A., Temtamy, S., and Abd-Aziz, K.B. (1983) *Chromosomal studies on laboratory rats (Rattus norvegicus) exposed to an organic solvent (cyclohexanone)*.

155. EFSA (2009) Scientific Opinion on the evaluation of substances as acceptable previous cargoes for edible fats and oils. EFSA Panel on Contaminants in the Food Chain (CONTAM)2Retrieved from [web site]: <https://efsa.onlinelibrary.wiley.com/doi/pdf/10.2903/j.efsa.2009.1391>. *EFSA Journal*, **7**, 1391.

156. IARC (1999) Re-evaluation of Some Organic Chemicals, Hydrazine and Hydrogen Peroxide (Part 1, Part 2, Part 3) IARC Monographs on the Evaluation of Carcinogenic Risks to Humans. <https://monographs.iarc.fr/wp-content/uploads/2018/06/mono71.pdf>, pp. 1359-1364.

157. Zeiger, E., Anderson, B., Haworth, S., Lawlor, T., and Mortelmans, K. (1992) Salmonella mutagenicity tests: V. Results from the testing of 311 chemicals. *Environmental and molecular mutagenesis*, **19 Suppl 21**, 2-141.

158. EMEA (2003) Diclofenac Summary Report EMEA/MRL/885/03-FINAL, September 2003. The European Agengy for the Evaluation of Medicinal Products. Veterinary Medicines and Inspections. [web site] <http://www.ema.europa.eu/docs/en_GB/document_library/Maximum_Residue_Limits_-_Report/2009/11/WC500013751.pdf>.

159. Stoyanov, I.S., Nicolov, I.G., Chernozemsky, I.N., and Stoichev, I. (1987) Assessment for mutagenicity of 10 pharmaceutical products following ames, micronucleus, and sperm morphology testing. *Toxicity Assessment*, **2**, 207-215.

160. Kuboyama, N., and Fujii, A. (1992) Mutagenicity of analgesics, their derivatives, and anti-inflammatory drugs with S-9 mix of several animal species. *J Nihon Univ Sch Dent*, **34**, 183-195.

161. Kadotani, S., Arisawa, M., and Maruyama, H.B. (1984) Mutagenicity examination of several non-steroidal anti-inflammatory drugs in bacterial systems. *Mutation research*, **138**, 133-136.

162. Snyder, R.D., and Green, J.W. (2001) A review of the genotoxicity of marketed pharmaceuticals. *Mutation research*, **488**, 151-169.

163. Food and Drug Administration (FDA) [web site], <http://www.fda.gov/>.

164. Kirkland, D., Kasper, P., Martus, H.J., Muller, L., van Benthem, J., Madia, F., and Corvi, R. (2016) Updated recommended lists of genotoxic and non-genotoxic chemicals for assessment of the performance of new or improved genotoxicity tests. *Mutat Res Genet Toxicol Environ Mutagen*, **795**, 7-30.

165. NTP Testing Status of Ethionamide 11621-W. National Toxicology Program [web site], <https://ntp.niehs.nih.gov/testing/status/agents/ts-11621-w.html>, (accessed July 1 2019).

166. Pant, K., Krsmanovic, L., Bruce, S.W., Kelley, T., Arevalo, M., Atta-Safoh, S., Debelie, F., La Force, M.L., Springer, S., Sly, J., Paranjpe, M., Lawlor, T., and Aardema, M. (2015) Combination comet/micronucleus assay validation performed by BioReliance under the JaCVAM initiative. *Mutat Res Genet Toxicol Environ Mutagen*, **786-788**, 87-97.

167. Matsushima, T., Hayashi, M., Matsuoka, A., Ishidate, M., Jr., Miura, K.F., Shimizu, H., Suzuki, Y., Morimoto, K., Ogura, H., Mure, K., Koshi, K., and Sofuni, T. (1999) Validation study of the in vitro micronucleus test in a Chinese hamster lung cell line (CHL/IU). *Mutagenesis*, **14**, 569-580.

168. Latt, S.A., Allen, J., Bloom, S.E., Carrano, A., Falke, E., Kram, D., Schneider, E., Schreck, R., Tice, R., Whitfield, B., and Wolff, S. (1981) Sister-chromatid exchanges: a report of the GENE-TOX program. *Mutation research*, **87**, 17-62.

169. NTP Testing Status of Ethyl methanesulfonate M20304. National Toxicology Program [web site], <https://ntp.niehs.nih.gov/testing/status/agents/ts-m20304.html>, (accessed July 1 2019).

170. Clive, D. (1977) Linear relationship between tumorigenic potency in vivo and mutagenic potency at the heterozygous thymidine kinase (T+/-) locus of L5178Y mouse lymphoma cells coupled with mammalian metabolism. *Progress in Genetic Toxicology, Amsterdam, Elsevier/North Holland*, 241-247.

171. Ogawa, I., Furukawa, S., Abe, M., Tanaka, Y., Hayashi, S., and Usuda, K. (2009) Multi-endpoint genotoxic assay using L5178Y (Tk(+/-) -3.7.2c) cells. *The Journal of Toxicological Sciences*, **34**, 547-553.

172. Bradley, M.O., Bhuyan, B., Francis, M.C., Langenbach, R., Peterson, A., and Huberman, E. (1981) Mutagenesis by chemical agents in V79 chinese hamster cells: a review and analysis of the literature. A report of the Gene-Tox Program. *Mutation research*, **87**, 81-142.

173. Hsie, A.W., Casciano, D.A., Couch, D.B., Krahn, D.F., O'Neill, J.P., and Whitfield, B.L. (1981) The use of Chinese hamster ovary cells to quantify specific locus mutation and to determine mutagenicity of chemicals. A report of the gene-tox program. *Mutation research*, **86**, 193-214.

174. Gocke, E., Ballantyne, M., Whitwell, J., and Muller, L. (2009) MNT and MutaMouse studies to define the in vivo dose response relations of the genotoxicity of EMS and ENU. *Toxicology letters*, **190**, 286-297.

175. Chiu, S.W., Wang, Z.M., Leung, T.M., and Moore, D. (2000) Nutritional value of ganoderma extract and assessment of its genotoxicity and antigenotoxicity using comet assays of mouse lymphocytes. *Food Chem Toxicol*, **38**, 173-178.

176. Naya, M., Kobayashi, N., Endoh, S., Maru, J., Honda, K., Ema, M., Tanaka, J., Fukumuro, M., Hasegawa, K., Nakajima, M., Hayashi, M., and Nakanishi, J. (2012) In vivo genotoxicity study of single-wall carbon nanotubes using comet assay following intratracheal instillation in rats. *Regul Toxicol Pharmacol*, **64**, 124-129.

177. Recio, L., Hobbs, C., Caspary, W., and Witt, K.L. (2010) Dose-response assessment of four genotoxic chemicals in a combined mouse and rat micronucleus (MN) and Comet assay protocol. *J Toxicol Sci*, **35**, 149-162.

178. Recio, L., Kissling, G.E., Hobbs, C.A., and Witt, K.L. (2012) Comparison of Comet assay dose-response for ethyl methanesulfonate using freshly prepared versus cryopreserved tissues. *Environmental and molecular mutagenesis*, **53**, 101-113.

179. Wada, K., Ohnuma, A., Kojima, S., Yoshida, T., and Matsumoto, K. (2012) A comparison of cell-collecting methods for the Comet assay in urinary bladders of rats. *Mutation research*, **742**, 26-30.

180. Anderson, D., and Richardson, C.R. (1981) Issues relevant to the assessment of chemically induced chromosome damage in vivo and their relationship to chemical mutagenesis. *Mutation research*, **90**, 261-272.

181. IARC (2000) Etoposide. *IARC Working Group on the Evaluation of Carcinogenic Risk to Humans. Some Antiviral and Antineoplastic Drugs, and Other Pharmaceutical Agents*, <https://monographs.iarc.fr/wp-content/uploads/2018/06/mono76-10.pdf>, pp. 177-257.

182. Nakanomyo, H., Hiraoka, M., and Shiraya, M. (1986) [Mutagenicity tests of etoposide and teniposide]. *J Toxicol Sci*, **11 Suppl 1**, 301-310.

183. Gupta, R.S., Bromke, A., Bryant, D.W., Gupta, R., Singh, B., and McCalla, D.R. (1987) Etoposide (VP16) and teniposide (VM26): novel anticancer drugs, strongly mutagenic in mammalian but not prokaryotic test systems. *Mutagenesis*, **2**, 179-186.

184. Ashby, J., Tinwell, H., Glover, P., Poorman-Allen, P., Krehl, R., Callander, R.D., and Clive, D. (1994) Potent clastogenicity of the human carcinogen etoposide to the mouse bone marrow and mouse lymphoma L5178Y cells: comparison to Salmonella responses. *Environmental and molecular mutagenesis*, **24**, 51-60.

185. Bryce, S.M., Bemis, J.C., Avlasevich, S.L., and Dertinger, S.D. (2007) In vitro micronucleus assay scored by flow cytometry provides a comprehensive evaluation of cytogenetic damage and cytotoxicity. *Mutation research*, **630**, 78-91.

186. Boos, G., and Stopper, H. (2000) Genotoxicity of several clinically used topoisomerase II inhibitors. *Toxicology letters*, **116**, 7-16.

187. Garriott, M.L., Phelps, J.B., and Hoffman, W.P. (2002) A protocol for the in vitro micronucleus test. I. Contributions to the development of a protocol suitable for regulatory submissions from an examination of 16 chemicals with different mechanisms of action and different levels of activity. *Mutation research*, **517**, 123-134.

188. Godard, T., Fessard, V., Huet, S., Mourot, A., Deslandes, E., Pottier, D., Hyrien, O., Sichel, F., Gauduchon, P., and Poul, J. (1999) Comparative in vitro and in vivo assessment of genotoxic effects of etoposide and chlorothalonil by the comet assay. *Mutation research*, **444**, 103-116.

189. Sekizawa, J., and Shibamoto, T. (1982) Genotoxicity of safrole-related chemicals in microbial test systems. *Mutation research*, **101**, 127-140.

190. Maralhas, A., Monteiro, A., Martins, C., Kranendonk, M., Laires, A., Rueff, J., and Rodrigues, A.S. (2006) Genotoxicity and endoreduplication inducing activity of the food flavouring eugenol. *Mutagenesis*, **21**, 199-204.

191. Ishidate, M., Jr., Sofuni, T., Yoshikawa, K., Hayashi, M., Nohmi, T., Sawada, M., and Matsuoka, A. (1984) Primary mutagenicity screening of food additives currently used in Japan. *Food Chem Toxicol*, **22**, 623-636.

192. Stich, H.F., Stich, W., and Lam, P.P. (1981) Potentiation of genotoxicity by concurrent application of compounds found in betel quid: arecoline, eugenol, quercetin, chlorogenic acid and Mn2+. *Mutation research*, **90**, 355-363.

193. Woolverton, C.J., Fotos, P.G., Mokas, M.J., and Mermigas, M.E. (1986) Evaluation of eugenol for mutagenicity by the mouse micronucleus test. *J Oral Pathol*, **15**, 450-453.

194. Ellahuene, M.F., Perez-Alzola, L.P., Orellana-Valdebenito, M., Munoz, C., and Lafuente-Indo, N. (1994) Genotoxic evaluation of eugenol using the bone marrow micronucleus assay. *Mutation research*, **320**, 175-180.

195. Shelby, M.D., Erexson, G.L., Hook, G.J., and Tice, R.R. (1993) Evaluation of a three-exposure mouse bone marrow micronucleus protocol: results with 49 chemicals. *Environmental and molecular mutagenesis*, **21**, 160-179.

196. Maura, A., Pino, A., and Ricci, R. (1989) Negative evidence in vivo of DNA-damaging, mutagenic and chromosomal effects of eugenol. *Mutation research*, **227**, 125-129.

197. Allavena, A., Martelli, A., Robbiano, L., and Brambilla, G. (1992) Evaluation in a battery of in vivo assays of four in vitro genotoxins proved to be noncarcinogens in rodents. *Teratog Carcinog Mutagen*, **12**, 31-41.

198. Hayashi, M., Kishi, M., Sofuni, T., and Ishidate, M., Jr. (1988) Micronucleus tests in mice on 39 food additives and eight miscellaneous chemicals. *Food Chem Toxicol*, **26**, 487-500.

199. Rompelberg, C.J., Steenwinkel, M.J., van Asten, J.G., van Delft, J.H., Baan, R.A., and Verhagen, H. (1996) Effect of eugenol on the mutagenicity of benzo[a]pyrene and the formation of benzo[a]pyrene-DNA adducts in the lambda-lacZ-transgenic mouse. *Mutation research*, **369**, 87-96.

200. IARC (2001) Griseofulvin. *IARC Working Group on the Evaluation of Carcinogenic Risk to Humans. Some Thyrotropic Agents*, <https://monographs.iarc.fr/wp-content/uploads/2018/06/mono79-12.pdf>, pp. 291-315.

201. Fujita, H., and Sasaki, M. (1990) Mutagenicity test of food additives with Salmonella typhimurium TA97 and TA102 (V). *Annual Report of the Tokyo Metropolitan Research Laboratory of Public Health*, **41**, 315-322.

202. Gulati, D.K., Witt, K., Anderson, B., Zeiger, E., and Shelby, M.D. (1989) Chromosome aberration and sister chromatid exchange tests in Chinese hamster ovary cells in vitro. III. Results with 27 chemicals. *Environmental and molecular mutagenesis*, **13**, 133-193.

203. Myhr, B.C. (1991) Validation studies with Muta Mouse: a transgenic mouse model for detecting mutations in vivo. *Environmental and molecular mutagenesis*, **18**, 308-315.

204. NTP Testing Status of D-Mannitol 10386-L. National Toxicology Program [web site], <https://ntp.niehs.nih.gov/testing/status/agents/ts-10386-l.html>, (accessed July 1 2019).

205. Shelby, M.D., and Witt, K.L. (1995) Comparison of results from mouse bone marrow chromosome aberration and micronucleus tests. *Environmental and molecular mutagenesis*, **25**, 302-313.

206. Zeiger, E. (1990) Mutagenicity of 42 chemicals in Salmonella. *Environmental and molecular mutagenesis*, **16 Suppl 18**, 32-54.

207. OECD (1997) SISD Initial Assessment Report For SIAM 6 on 1-chlorobuthane, UNEP Publications, 1–41., <https://hpvchemicals.oecd.org/ui/handler.axd?id=D57313CB-BD84-454D-8255-6D78BEC7FFC0>.

208. Sobol, Z., Engel, M.E., Rubitski, E., Ku, W.W., Aubrecht, J., and Schiestl, R.H. (2007) Genotoxicity profiles of common alkyl halides and esters with alkylating activity. *Mutation research*, **633**, 80-94.

209. Oesch, F., Bucker, M., and Glatt, H.R. (1981) Activation of phenanthrene to mutagenic metabolites and evidence for at least two different activation pathways. *Mutation research*, **81**, 1-10.

210. Bucker, M., Glatt, H.R., Platt, K.L., Avnir, D., Ittah, Y., Blum, J., and Oesch, F. (1979) Mutagenicity of phenanthrene and phenanthrene K-region derivatives. *Mutation research*, **66**, 337-348.

211. Sakai, M., Yoshida, D., and Mizusaki, S. (1985) Mutagenicity of polycyclic aromatic hydrocarbons and quinones on Salmonella typhimurium TA97. *Mutation research*, **156**, 61-67.

212. Sasaki, J.C., Arey, J., Eastmond, D.A., Parks, K.K., and Grosovsky, A.J. (1997) Genotoxicity induced in human lymphoblasts by atmospheric reaction products of naphthalene and phenanthrene. *Mutation research*, **393**, 23-35.

213. Crofton-Sleigh, C., Doherty, A., Ellard, S., Parry, E.M., and Venitt, S. (1993) Micronucleus assays using cytochalasin-blocked MCL-5 cells, a proprietary human cell line expressing five human cytochromes P-450 and microsomal epoxide hydrolase. *Mutagenesis*, **8**, 363-372.

214. Durant, J.L., Busby, W.F., Jr., Lafleur, A.L., Penman, B.W., and Crespi, C.L. (1996) Human cell mutagenicity of oxygenated, nitrated and unsubstituted polycyclic aromatic hydrocarbons associated with urban aerosols. *Mutation research*, **371**, 123-157.

215. Abramsson-Zetterberg, L., and Maurer, B.M. (2015) Fluoranthene and phenantrene, two predominant PAHs in heat-prepared food, do not influence the frequency of micronucleated mouse erythrocytes induced by other PAHs. *Toxicol Rep*, **2**, 1057-1063.

216. Zeiger, E., Haworth, S., Mortelmans, K., and Speck, W. (1985) Mutagenicity testing of di(2-ethylhexyl)phthalate and related chemicals in Salmonella. *Environ Mutagen*, **7**, 213-232.

217. NTP Testing Status of Phthalic anhydride 10421-A. National Toxicology Program [web site], <https://ntp.niehs.nih.gov/testing/status/agents/ts-10421-a.html>, (accessed July 1 2019).

218. PubChem National Center for Biotechnology Information. 4-Nitrophenol, CID=980, PubChem Online Database <https://pubchem.ncbi.nlm.nih.gov/compound/4-Nitrophenol> (accessed on July 7, 2019). .

219. Shimizu, M., and Yano, E. (1986) Mutagenicity of mono-nitrobenzene derivatives in the Ames test and rec assay. *Mutation research*, **170**, 11-22.

220. Kawai, A., Goto, S., Matsumoto, Y., and Matsushita, H. (1987) [Mutagenicity of aliphatic and aromatic nitro compounds. Industrial materials and related compounds]. *Sangyo Igaku*, **29**, 34-54.

221. Dellarco, V.L., and Prival, M.J. (1989) Mutagenicity of nitro compounds in Salmonella typhimurium in the presence of flavin mononucleotide in a preincubation assay. *Environmental and molecular mutagenesis*, **13**, 116-127.

222. NTP Testing Status of p-Nitrophenol 10142-E. National Toxicology Program [web site], <https://ntp.niehs.nih.gov/whatwestudy/testpgm/status/ts-10142-e.html>, (accessed July 1 2019).

223. Huang, Q.G., Kong, L.R., Liu, Y.B., and Wang, L.S. (1996) Relationships between molecular structure and chromosomal aberrations in in vitro human lymphocytes induced by substituted nitrobenzenes. *Bull Environ Contam Toxicol*, **57**, 349-353.

224. Eichenbaum, G., Johnson, M., Kirkland, D., O'Neill, P., Stellar, S., Bielawne, J., DeWire, R., Areia, D., Bryant, S., Weiner, S., Desai-Krieger, D., Guzzie-Peck, P., Evans, D.C., and Tonelli, A. (2009) Assessment of the genotoxic and carcinogenic risks of p-nitrophenol when it is present as an impurity in a drug product. *Regul Toxicol Pharmacol*, **55**, 33-42.

225. IARC (1999) Potassium bromate. *IARC Working Group on the Evaluation of Carcinogenic Risk to Humans. Some Chemicals that Cause Tumours of the Kidney or Urinary Bladder in Rodents and Some Other Substances*, <https://monographs.iarc.fr/wp-content/uploads/2018/06/mono73-22.pdf>, pp. 481-496.

226. Speit, G., Haupter, S., Schutz, P., and Kreis, P. (1999) Comparative evaluation of the genotoxic properties of potassium bromate and potassium superoxide in V79 Chinese hamster cells. *Mutation research*, **439**, 213-221.

227. Nakajima, M., Kitazawa, M., Oba, K., Kitagawa, Y., and Toyoda, Y. (1989) Effect of route of administration in the micronucleus test with potassium bromate. *Mutation research*, **223**, 399-402.

228. Bianchi, V., Celotti, L., Lanfranchi, G., Majone, F., Marin, G., Montaldi, A., Sponza, G., Tamino, G., Venier, P., Zantedeschi, A., and Levis, A.G. (1983) Genetic effects of chromium compounds. *Mutation research*, **117**, 279-300.

229. Stella, M., Montaldi, A., Rossi, R., Rossi, G., and Levis, A.G. (1982) Clastogenic effects of chromium on human lymphocytes in vitro and in vivo. *Mutation research*, **101**, 151-164.

230. Uyeki, E.M., and Nishio, A. (1983) Antiproliferative and genotoxic effects of chromium on cultured mammalian cells. *Journal of Toxicology and Environmental Health*, **11**, 227-235.

231. ECHA Potassium dichromate, ECHA database <https://echa.europa.eu/registration-dossier/-/registered-dossier/15102/7/7/3/?documentUUID=7e394601-3ee3-4c7a-a8a1-a49ac36a93d6> (accessed July 1, 2019).

232. EFSA (2014) Scientific Opinion on the re-evaluation of propyl gallate (E 310) as a food additive. *EFSA Journal*, **12**, 3642.

233. Abdo, K.M., Huff, J.E., Haseman, J.K., and Alden, C.J. (1986) No evidence of carcinogenicity of D-mannitol and propyl gallate in F344 rats or B6C3F1 mice. *Food Chem Toxicol*, **24**, 1091-1097.

234. Hakura, A., Tsutsui, Y., Mochida, H., Sugihara, Y., Mikami, T., and Sagami, F. (1996) Mutagenicity of dihydroxybenzenes and dihydroxynaphthalenes for Ames Salmonella tester strains. *Mutation research*, **371**, 293-299.

235. JETOC (1997) Mutagenicity test data of existing chemical substances based on the toxicity investigation system of the industrial safety and health law. Supplement. Japan Chemical Industry Ecology-Toxicology & Information Center (JETOC), Chuo-ku, Tokyo, Japan.

236. Wild, D., King, M.-T., Eckhardt, I.C., and Gocke, E. (1981) Mutagenic activity of aminophenols and diphenols, and relations with chemiccal structure. *Mutat. Res.*, **85**, 456.

237. Jansson, T., Curvall, M., Hedin, A., and Enzell, C.R. (1986) In vitro studies of biological effects of cigarette smoke condensate. II. Induction of sister-chromatid exchanges in human lymphocytes by weakly acidic, semivolatile constituents. *Mutation research*, **169**, 129-139.

238. NTP Testing Status of Resorcinol 10163-Y. National Toxicology Program [web site], <https://ntp.niehs.nih.gov/testing/status/agents/ts-10163-y.html>, (accessed July 1 2019).

239. Darroudi, F., and Natarajan, A.T. (1983) Cytogenetic analysis of human peripheral blood lymphocytes (in vitro) treated with resorcinol. *Mutation research*, **124**, 179-189.

240. Sakano, Y., Tsuyoshi, T., Kobayashi, Y., Andoh, H., and Masamoto, Y. (1985) The role of oxygen freeradicals in the mutagenesis of divalent phenols. *Mutation research*, **147**, 272-273.

241. Stich, H.F., Rosin, M.P., Wu, C.H., and Powrie, W.D. (1981) The action of transition metals on the genotoxicity of simple phenols, phenolic acids and cinnamic acids. *Cancer Lett*, **14**, 251-260.

242. Natarajan, A.T., and Obe, G. (1986) How do in vivo mammalian assays compare to in vitro assays in their ability to detect mutagens? *Mutation research*, **167**, 189-201.

243. Bracher, M., Swistak, J., and Noser, F. (1981) Studies on the potential in vivo induction of sister-chromatid exchanges in rat bone marrow by resorcinol. *Mutation research*, **91**, 363-369.

244. Ma, H., Wang, Z., and Liao, M. (1996) Genetic toxicity of taxol. *Zhongguo Yaolixue Yu Dulixue Zazhi*, **10**, 173-177.

245. Le Fevre, A.C., Boitier, E., Marchandeau, J.P., Sarasin, A., and Thybaud, V. (2007) Characterization of DNA reactive and non-DNA reactive anticancer drugs by gene expression profiling. *Mutation research*, **619**, 16-29.

246. Digue, L., Orsiere, T., De Meo, M., Mattei, M.G., Depetris, D., Duffaud, F., Favre, R., and Botta, A. (1999) Evaluation of the genotoxic activity of paclitaxel by the in vitro micronucleus test in combination with fluorescent in situ hybridization of a DNA centromeric probe and the alkaline single cell gel electrophoresis technique (comet assay) in human T-lymphocytes. *Environmental and molecular mutagenesis*, **34**, 269-278.

247. Steiblen, G., Orsiere, T., Pallen, C., Botta, A., and Marzin, D. (2005) Comparison of the relative sensitivity of human lymphocytes and mouse splenocytes to two spindle poisons. *Mutation research*, **588**, 143-151.

248. Hashimoto, K., Nakajima, Y., Matsumura, S., and Chatani, F. (2010) An in vitro micronucleus assay with size-classified micronucleus counting to discriminate aneugens from clastogens. *Toxicology in vitro : an international journal published in association with BIBRA*, **24**, 208-216.

249. Wang, J., Sawyer, J.R., Chen, L., Chen, T., Honma, M., Mei, N., and Moore, M.M. (2009) The mouse lymphoma assay detects recombination, deletion, and aneuploidy. *Toxicol Sci*, **109**, 96-105.

250. Muehlbauer, P.A., and Schuler, M.J. (2005) Detection of numerical chromosomal aberrations by flow cytometry: a novel process for identifying aneugenic agents. *Mutation research*, **585**, 156-169.

251. Tinwell, H., and Ashby, J. (1994) Genetic toxicity and potential carcinogenicity of taxol. *Carcinogenesis*, **15**, 1499-1501.

252. Mughal, A., Vikram, A., Ramarao, P., and Jena, G.B. (2010) Micronucleus and comet assay in the peripheral blood of juvenile rat: establishment of assay feasibility, time of sampling and the induction of DNA damage. *Mutation research*, **700**, 86-94.

253. Igarashi, M., Setoguchi, M., Takada, S., Itoh, S., and Furuhama, K. (2007) Optimum conditions for detecting hepatic micronuclei caused by numerical chromosome aberration inducers in mice. *Mutation research*, **632**, 89-98.

254. Müller KR, L.B.J. (1983) In vitro genetic activity report: evaluation of mono-tertiary butylhydroquinone in the Ames Salmonella/microsome bacterial mutagenesis test. Unpublished report from Health, Safety and Human Factors Laboratory, Eastman Kodak Co., Rochester, NY, USA. Submitted to WHO by Eastman Kodak Co., Kingsport, TN, USA.

255. Hageman, G.J., Verhagen, H., and Kleinjans, J.C. (1988) Butylated hydroxyanisole, butylated hydroxytoluene and tert.-butylhydroquinone are not mutagenic in the Salmonella/microsome assay using new tester strains. *Mutation research*, **208**, 207-211.

256. Matsuoka, A., Matsui, M., Miyata, N., Sofuni, T., and Ishidate, M., Jr. (1990) Mutagenicity of 3-tert-butyl-4-hydroxyanisole (BHA) and its metabolites in short-term tests in vitro. *Mutation research*, **241**, 125-132.

257. NTP Testing Status of t-Butylhydroquinone 1948330. National Toxicology Program [web site], <https://ntp.niehs.nih.gov/testing/status/agents/ts-1948330.html>, (accessed July 1 2019).

258. NTP Testing Status of Sodium xylenesulfonate 10184-T. National Toxicology Program [web site], <https://ntp.niehs.nih.gov/testing/status/agents/ts-10184-t.html>, (accessed July 1 2019).

259. Barfield, W., and Burlinson, B. (2015) p-Chloroaniline, t-butylhydroquinone, and methyl carbamate: Rat in vivo comet test, JaCVAM trial phase 4.2. *Mutat Res Genet Toxicol Environ Mutagen*, **786-788**, 98-103.

260. IPCS TBHQ (Tert-Butylhydroquinone). International Programme on Chemical Safety (IPCS) [website], <http://www.inchem.org/documents/jecfa/jecmono/v21je04.htm>, (accessed on July 1 2019).
